# Photothermal Superhydrophobic Textiles: An Emerging Paradigm from Passive Water Repellency to Active Thermal Management

**DOI:** 10.1007/s40820-026-02323-4

**Published:** 2026-08-10

**Authors:** Tianyu Sheng, Haoyang Wang, Ke Pei, Chengjin Zhang, Huiyu Jiang, Hongyun Peng, Zhiwen Zhou, Zhiguang Guo

**Affiliations:** 1 Ministry of Education Key Laboratory for the Green Preparation and Application of Functional Materials, School of Materials Science and Engineering, Hubei University, Wuhan, 430062 People’s Republic of China; 2 State Key Laboratory of New Textile Materials and Advanced Processing, Wuhan Textile University, Wuhan, 430200 People’s Republic of China; 3 School of Materials Science and Engineering, Harbin Institute of Technology, Harbin, 150001 People’s Republic of China; 4 State Key Laboratory of Solid Lubrication, Lanzhou Institute of Chemical Physics, Chinese Academy of Sciences, Lanzhou, 730000 People’s Republic of China

**Keywords:** Superhydrophobic surfaces, Photothermal conversion, Intelligent textiles, Synergistic interplay, Lab-to-life translation

## Abstract

Photothermal superhydrophobic textiles represent an emerging paradigm integrating active light-driven functionality with passive liquid repellency. While previous reviews treated superhydrophobicity and photothermal effects in isolation, this work pioneers a systematic analysis of their synergistic interplay—a critical, underexplored mechanism where superhydrophobicity preserves photothermal efficiency by minimizing water-induced heat loss, while photothermal activity prevents surface fouling that compromises non-wetting performance. Through detailed case studies of advanced material systems, this review highlights how this synergy supports significant advancements in adaptive wearable technology, energy-efficient infrastructure, and eco-friendly water treatment. This review presents an original “lab-to-life” roadmap that structurally links material design to durability assessment, environmental impact evaluation, and scalable manufacturing strategies—a holistic framework absent in existing literature. The review emphasizes urgent sustainability priorities including green material substitutions, non-toxic solvent processing methodologies, and circular design principles aligned with global environmental regulations. By connecting fundamental mechanisms to real-world deployment scenarios while outlining transformative future directions such as AI-accelerated material discovery, stimulus-responsive systems, and intelligent manufacturing protocols, this work provides a timely and actionable reference for advancing next-generation textiles toward technological sophistication and practical viability in sustainable development.

## Highlights

- This review proposes a representative framework for photothermal superhydrophobic textiles, highlighting the transition from single-material studies toward integrated, system-level design and intelligent applications.
- This review deciphers the synergistic mechanism between photothermal conversion and superhydrophobicity, linking nano‑/micro‑scale material design to active de‑icing and self‑cleaning performance.
- This review critically analyzes scalable fabrication, real‑world applications, and key challenges, while outlining AI‑guided optimization and stimulus‑responsive systems as future pathways.

## Graphical abstract

**Figure:**
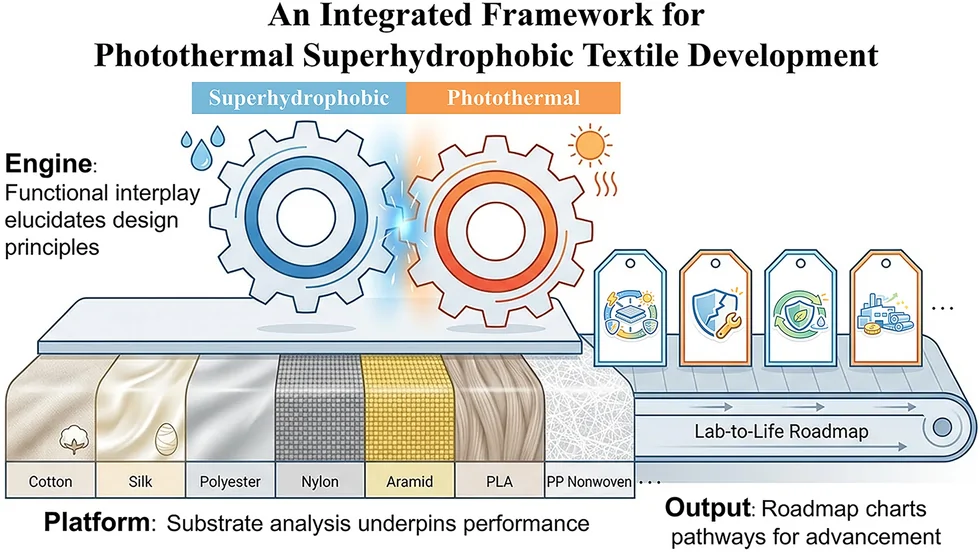

## Introduction

Textiles, one of humanity’s oldest technologies, are undergoing a revolutionary transformation from passive materials providing basic comfort to active, multifunctional interfaces that dynamically interact with their environment [1–5]. At the forefront of this evolution are superhydrophobic textiles, which draw inspiration from natural paradigms like the lotus leaf to achieve exceptional water repellency and self-cleaning capabilities [6–10]. By creating micro-/nanoscale hierarchical structures and modifying surface chemistry, these materials can trap air pockets, leading to the stable Cassie-Baxter state that prevents water penetration [11–15]. This passive resistance to water, stains, and microorganisms has paved the way for advanced applications in protective clothing, anti-fouling fabrics, and fluid management systems [16–20]. However, the inherent passivity of conventional superhydrophobic surfaces presents a significant limitation. Their performance is largely reactive, dependent on the presence of external contaminants or environmental challenges [21, 22]. Moreover, they offer no inherent mechanism to counteract challenges like ice accumulation in frigid environments, where water droplets can transition from a repellent Cassie-Baxter state to a pinned Wenzel state, leading to catastrophic icing [22, 23], or to manage heat in response to changing personal or environmental conditions [24]. The emerging demand for active and responsive smart textiles necessitates a paradigm shift beyond static functionality [21, 25]. It is here that the integration of photothermal functionality with superhydrophobicity unlocks a new dimension of intelligent behavior.

**Fig. 1:**
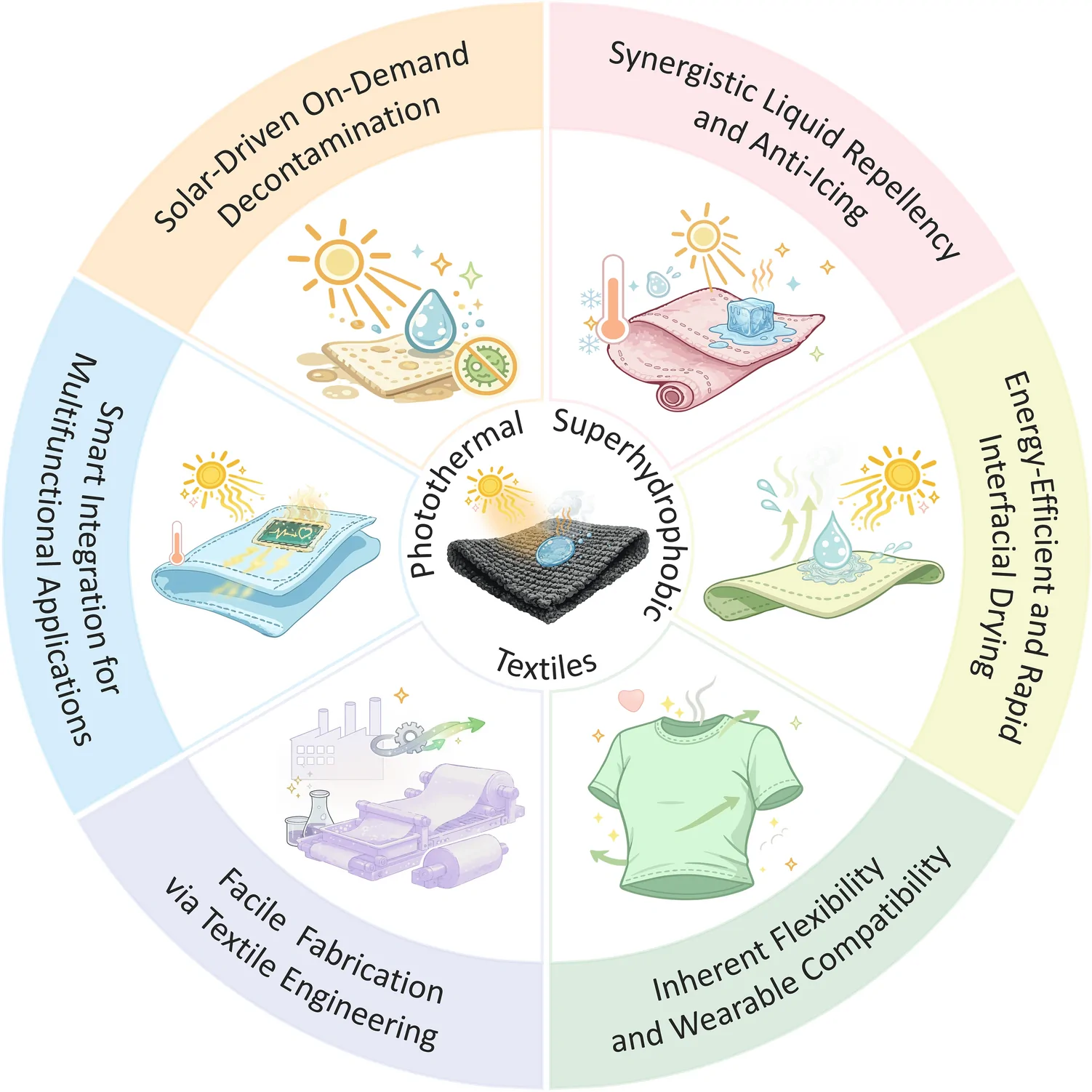
Key advantages and emerging applications of photothermal superhydrophobic textiles

The integration of photothermal functionality with superhydrophobicity marks this paradigm shift, creating textiles that are not merely protective but also adaptive and multi-functional. The cornerstone of this technology is the synergistic interplay between the physical microstructure and the photothermal nanomaterial. When illuminated, the embedded photothermal agents (e.g., plasmonic nanoparticles, carbon-based materials, conjugated polymers) efficiently convert light energy into localized heat [26–28]. This generated heat is not merely an added feature; it actively reinforces and enhances the superhydrophobic state. For instance, upon light exposure, the rapid Joule heating or non-radiative relaxation processes can instantly raise the surface temperature. This thermal energy can prevent condensation formation, a primary failure mechanism of superhydrophobic surfaces in humid environments [29, 30]. More profoundly, when water droplets reside on the surface, the localized heating can induce several active phenomena: it can create a vapor cushion beneath the droplet, leading to a levitating Leidenfrost state that drastically reduces friction and enhances self-propelled motion [31–34]; it can generate internal Marangoni flows within the droplet, prompting it to roll off spontaneously and remove surface contaminants more effectively than passive rolling alone [35, 36]; and critically, it can effectively melt frost or ice at the interface, providing an active anti-/de-icing capability that passive surfaces cannot offer [37, 38]. This transforms the textile from a static barrier into a dynamic interface. The resulting material platform exhibits a unique set of advantages: solar-driven on-demand decontamination through thermal inactivation of pathogens and enhanced self-cleaning [39–41]; synergistic liquid repellency and anti-icing by preventing water condensation and melting ice in real time [42]; energy-efficient and rapid interfacial drying due to the localized heating exactly at the liquid–solid–air interface [43, 44]; all while maintaining the inherent flexibility and wearable compatibility of the textile substrate [37]. This synergy, achievable through facile fabrication via textile engineering, opens the door to smart integration for multifunctional applications far beyond conventional clothing, spanning from adaptive personal thermal management to industrial ice protection systems and solar-driven water purification technologies, as conceptualized in Fig. 1.

While the individual domains of superhydrophobic textiles and photothermal coatings have been reviewed separately, a significant knowledge gap persists at their intersection. Existing literature typically treats either the superhydrophobic texture or the photothermal functionality in isolation, with limited discussion of their dynamic interplay. More critically, the role of the textile substrate itself has been largely overlooked—most reviews approach fabrics as passive scaffolds rather than active components with distinct morphological and capillary properties that significantly influence both droplet behavior and thermal management. Few previous reviews have comprehensively addressed the structure–property–application relationships specific to photothermal superhydrophobic textiles, particularly how fabric architecture, fiber chemistry, and surface topography jointly influence their performance. To clarify the positioning of this review, we compare it with several representative textile-related reviews (Table 1) [45–53]. These works cover superhydrophobic fabrics, photothermal materials, thermal management textiles, and other functional aspects, but most focus on a single functionality or a specific application, and few address the integrated design of photothermal superhydrophobic textiles as a coupled material platform. In contrast, the present review systematically examines the bidirectional coupling between photothermal functionality and superhydrophobicity, the role of textile substrates, substrate-dependent fabrication strategies, application-oriented performance requirements, and scalability challenges. This integrated perspective distinguishes our work from previous reviews and directly addresses the knowledge gap.

**Table 1:** Comparison of the present review with representative textile-related reviews

Refs.	Main focus	Photothermal functionality	Superhydrophobicity	PT-SH coupling	Textile substrate focus	Fabrication strategies	Application scenarios	Lab-to-life translation
[45]	Advanced textiles for personal thermal management and energy	✓	–	–	✓	✓	–	✓
[46]	MXene-based photothermal conversion and antibacterial applications	✓	–	–	–	✓	–	–
[47]	Superhydrophobic and antibacterial cellulose-based fibers/fabrics	–	✓	–	✓	✓	–	✓
[48]	Advanced fabrics for thermal management, health monitoring, and energy harvesting	–	–	–	✓	✓	–	✓
[49]	Thermal management fibers/textiles based on heat transport, storage, and conversion	✓	–	–	✓	✓	–	–
[50]	Bionic intelligent clothing	–	✓	–	✓	✓	–	✓
[51]	Advanced warming textiles based on radiative, insulating, and electrothermal strategies	✓	–	–	✓	✓	–	✓
[52]	Thermoregulating textiles for energy exchange and personal thermal management	✓	–	–	✓	✓	–	✓
[53]	Graphene-based textiles for thermal management and flame retardancy	✓	–	–	✓	✓	–	✓
**This review**	**Photothermal superhydrophobic textiles**	**✓**	**✓**	**✓**	**✓**	**✓**	**✓**	**✓**

PT-SH = photothermal superhydrophobicity

This review therefore addresses several underexplored dimensions that are crucial for both fundamental understanding and practical implementation. A critical observation driving this work is that the majority of current studies on photothermal superhydrophobic textiles remain at the stage of demonstrating collaborative phenomena—for instance, simultaneously reporting photothermal heating and water repellency—without probing the underlying mechanisms that govern their interaction. This phenomenological approach has left fundamental questions unanswered, such as how photothermal effects actively modulate the Cassie-Baxter state stability, or how superhydrophobicity in turn influences photothermal energy dissipation. To fill this gap, one of the core tasks of this review is to establish a structure–activity relationship model for the synergistic effects between superhydrophobicity and photothermal functionality, moving beyond phenomenological observation toward mechanistic understanding. First, we provide a thorough analysis of the Interplay between superhydrophobicity and photothermal functionality, elucidating how photothermal effects can actively maintain the Cassie-Baxter state rather than merely adding heating capability. Second, we introduce a classification system for photothermal superhydrophobic fabric substrates that moves beyond material composition to establish correlations between substrate properties (e.g., wettability, thermal conductivity, mechanical flexibility) and application-specific performance requirements. This framework specifically addresses how natural fibers and chemical fibers enable different application scenarios based on their intrinsic properties. Furthermore, we critically evaluate fabrication approaches specifically optimized for textile substrates, discussing how techniques like spray-coating and dip-padding affect durability and scalability differently than methods used on rigid surfaces.

Beyond technological applications, this review makes significant contributions to the academic community by establishing a unified theoretical framework that bridges interfacial science, photothermal chemistry, and textile engineering. We identify key scientific challenges that remain unresolved, such as the precise thermal threshold for optimal droplet mobility and the long-term stability of photothermal nanomaterials under mechanical deformation. By mapping the fundamental principles governing these multifunctional systems, we provide researchers with predictive design rules for developing next-generation smart textiles. The comprehensive application analysis spans from personal thermal management to environmental remediation, highlighting how photothermal superhydrophobic textiles can enable new research directions in areas like programmable droplet transport and solar-driven microfluidics. Finally, our “lab-to-life” perspective addresses a critical gap in existing literature by systematically evaluating scalability challenges and sustainability considerations. We propose standardized testing protocols specifically tailored for photothermal superhydrophobic textiles to enable meaningful cross-study comparisons. By connecting fundamental mechanisms with practical implementation barriers, this review aims to accelerate both academic innovation and industrial adoption of these multifunctional material systems, positioning photothermal superhydrophobic textiles as a platform technology for sustainable development across multiple disciplines. It is worth noting that photothermal heating and radiative cooling represent opposite thermal management directions; for radiative cooling superhydrophobic textiles, interested readers are referred to recent literature [54] and related comprehensive reviews [55, 56].

## Fundamental Principles of Superhydrophobic Surfaces

The development of photothermal superhydrophobic textiles is grounded in surface wetting principles. However, direct translation of wetting theories from rigid, smooth substrates to textiles is inadequate due to the hierarchical, flexible, and porous nature of fabric structures.

### Water Contact Angle (WCA)

**Fig. 2:**
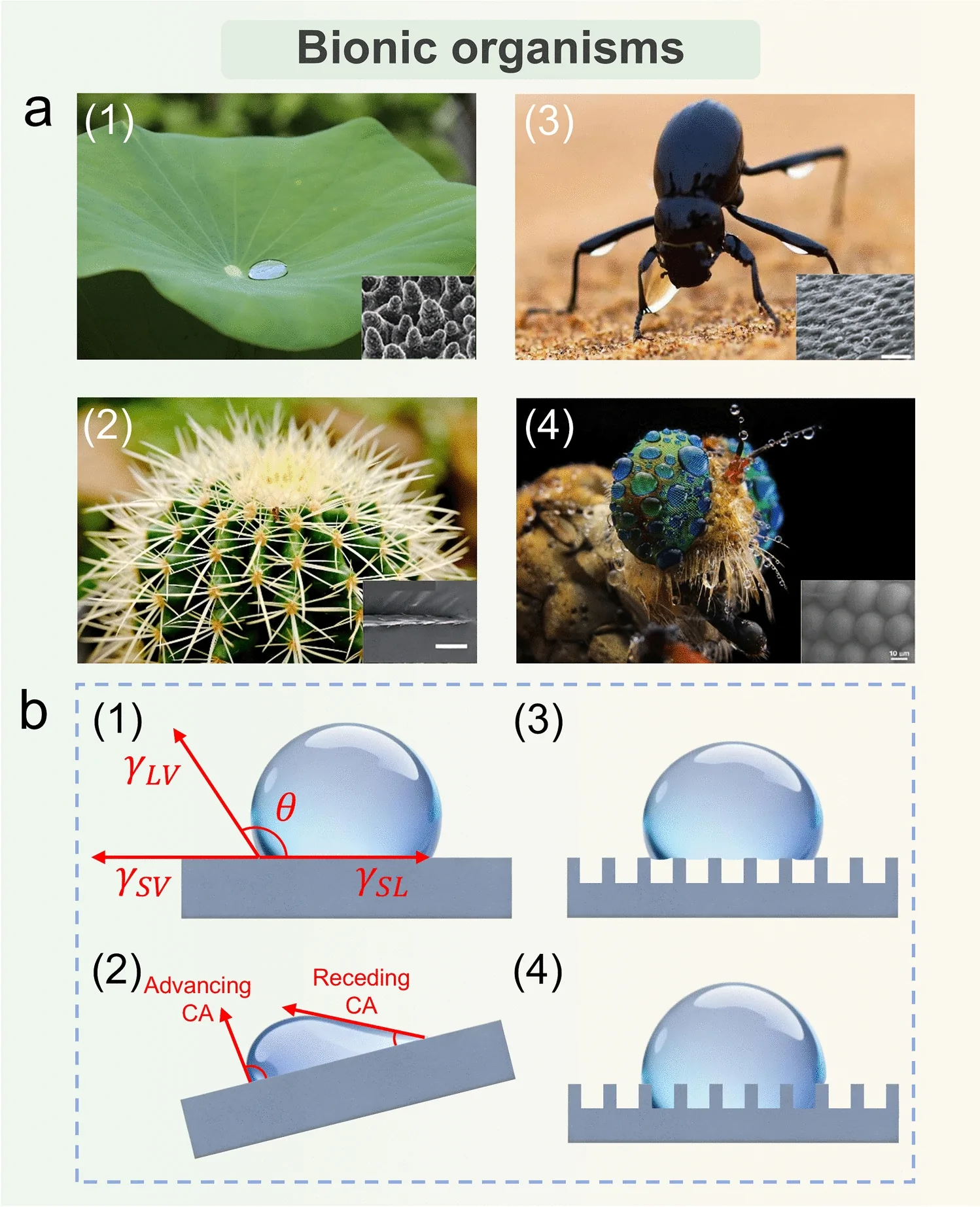
**a** Biological organisms exhibiting superhydrophobic properties in nature. (1) Lotus leaf: Reproduced with permission [60]. Copyright 2008, Wiley. (2) Cactus: Reproduced with permission [61]. Copyright 2020, Wiley. (3) Desert beetle: Reproduced with permission [62]. Copyright 2001, Springer Nature. (4) Mosquito eyes: Reproduced with permission [63]. Copyright 2007, Wiley. **b** Schematic illustration of the contact angles for (1) Young’s, (2) dynamic contact angles, (3) the Wenzel model, (4) Cassie-Baxter model

The water contact angle (WCA) is the primary metric for surface wettability. On an ideal flat surface, the equilibrium contact angle (*θ*_Y_) follows Young’s equation:

1 $$\cos \theta_{Y} = (\gamma_{{{\mathrm{SV}}}} - \gamma_{{{\mathrm{SL}}}} )/\gamma_{{{\mathrm{LV}}}}$$

where *γ*_SV_, *γ*_SL_, and *γ*_LV_ represent solid–vapor, solid–liquid, and liquid–vapor interfacial tensions, respectively [57–59]. Textiles fundamentally deviate from this ideal, being porous fiber assemblies with multi-scale roughness from nanoscale fiber features to micron-scale inter-fiber pores. Consequently, measuring a stable WCA on textiles is complicated by liquid wicking into pores. The apparent contact angle (*θ*) is dominated by surface topography and capillary effects rather than fiber chemistry alone. For textiles to achieve *θ* > 150°, both hydrophobic fiber chemistry (*θ*_*Y*_ > 90°) and a composite solid–air–liquid interface stabilized by hierarchical roughness are required. These design principles are inspired by biological systems, as illustrated in Fig. 2a. The lotus leaf (Fig. 2a(1)) exemplifies the “lotus effect” where hierarchical micropapillae coated with hydrophobic wax crystals create a composite interface with high contact angles and low adhesion [60]. The cactus (Fig. 2a(2)) uses asymmetric spines to repel water and directionally transport droplets for water collection [61]. The desert beetle (Fig. 2a(3)) employs hydrophilic bumps on a hydrophobic background to harvest fog [62], while the mosquito eye (Fig. 2a(4)) features a hexagonal nipple array conferring superhydrophobicity and antifogging properties [63]. These biological blueprints provide critical insights into geometric parameters for engineering robust superhydrophobic textiles on flexible, porous substrates.

### Wettability Models

The Wenzel and Cassie-Baxter models require careful consideration for textile substrates (Fig. 2b). The Wenzel model describes homogeneous wetting where liquid penetrates surface roughness [64]:

2 $$\cos \theta_{W} = \gamma \cos \theta_{Y}$$

where r is the roughness factor (*r* > 1). In textiles, a Wenzel state indicates liquid penetration into inter-fiber spaces, resulting in high adhesion, loss of breathability, and functional failure for protective clothing.

The Cassie–Baxter model describes a composite interface where air is trapped beneath the droplet [64, 65]:

3 $$\cos \theta_{CB} = f_{S} (1 + \cos \theta_{Y} ) - 1$$

where *f*_S_ is the fractional solid–liquid contact area. For textiles, *f*_S_ is dynamic—influenced by fiber density, yarn twist, and fabric structure—and can change under mechanical pressure due to fiber flexibility. The multi-scale hierarchy of textiles, combining nanoscale fiber roughness with microscale fabric pores, provides exceptional stability for the composite interface when properly engineered [66–69].

### Dynamic Contact Angle and Wetting Behavior of Composite Interfaces

Dynamic wetting behavior is characterized by contact angle hysteresis (CAH), defined as the difference between advancing (*θ*_A_) and receding contact angles (*θ*_R_) [70]:

4 $${\mathrm{CAH}} = \theta_{{\mathrm{A}}} - \theta_{{\mathrm{R}}}$$

A low CAH (< 10°) is essential for self-cleaning, but challenging to achieve on textiles due to surface heterogeneity and fiber flexibility. The complex fabric topography provides abundant pinning sites for the three-phase contact line, while fiber deformation under contact line tension further increases CAH, particularly during recession [71, 72]:

5 $$\theta_{{\mathrm{R}}} \propto \frac{1}{{E_{{{\mathrm{fiber}}}} }}$$

where *E*_fiber_ is the effective modulus of the fiber structure [47, 73]. Softer fibers generally exhibit higher CAH due to increased deformation at the contact line [71, 74–76]. Textile porosity introduces additional capillary effects [72, 77]:

6 $$P_{{{\mathrm{capillary}}}} = \frac{{2\gamma_{{{\mathrm{LV}}}} \cos \theta_{{\mathrm{Y}}} }}{{r_{{{\mathrm{pore}}}} }}$$

where *r*_pore_ is the effective pore radius [47, 73]. When *θ*_Y_ < 90°, positive capillary pressure drives liquid penetration; when *θ*_Y_ > 90°, negative pressure inhibits penetration, supporting the Cassie-Baxter state.

For photothermal superhydrophobic textiles, these dynamics are critical. Photothermal heating can reduce liquid surface tension and promote evaporation at the contact line, potentially mitigating the high CAH typical of textile substrates [78, 79].

In summary, wetting on textiles involves complex interactions between chemical hydrophobicity and physical morphology beyond classical models. The following sections detail how photothermal materials address these challenges. For a comprehensive treatment of fundamental wetting theory, readers are directed to specialized reviews [80–82].

## Fundamental Principles of Photothermal Surfaces

**Fig. 3:**
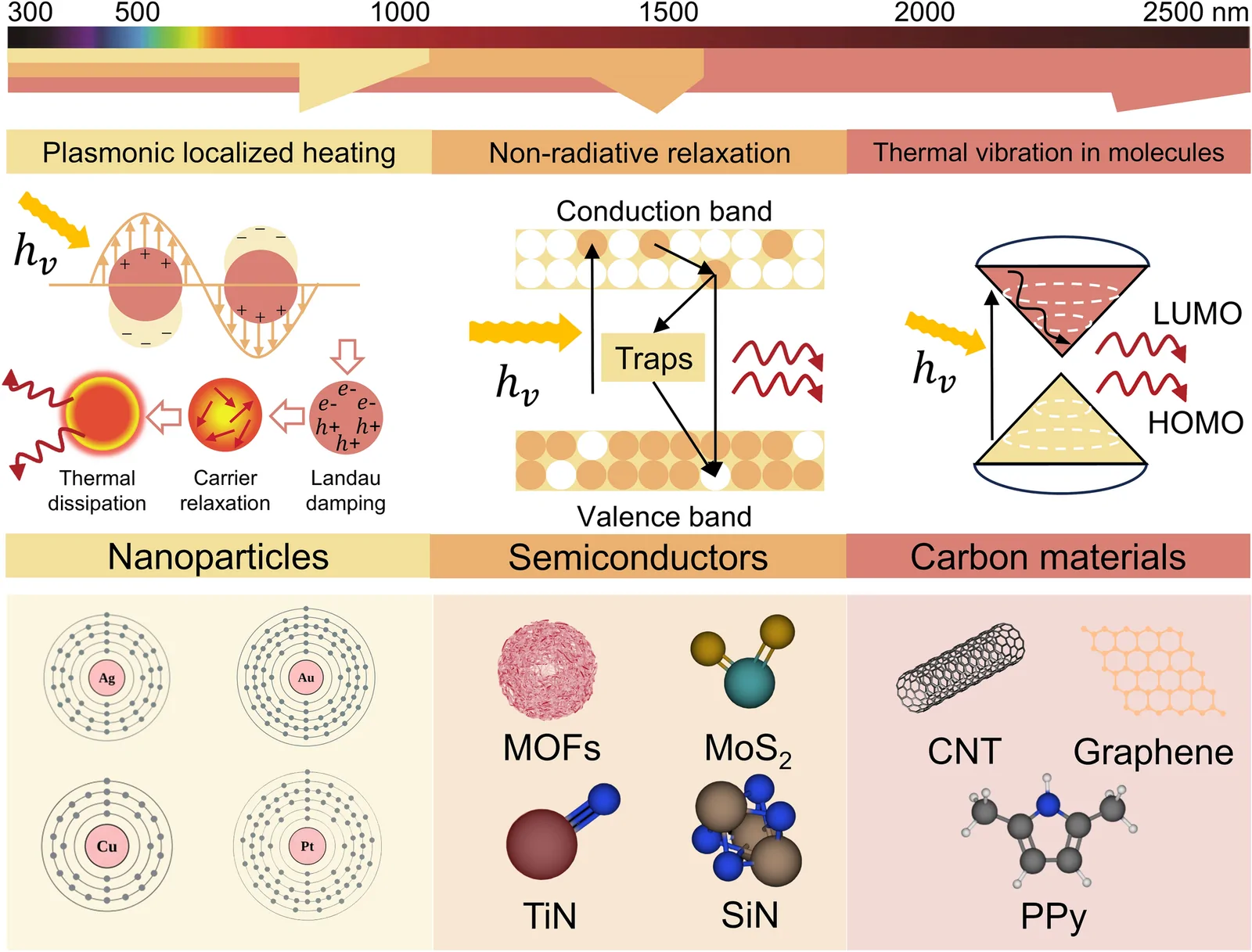
Three fundamental photothermal conversion mechanisms and representative photothermal materials for photothermal superhydrophobic textiles

Photothermal conversion involves transforming absorbed light energy into localized heat, governed by distinct physical mechanisms depending on the material’s electronic structure. Operating primarily within the solar spectrum (300–2500 nm), these mechanisms fall into three dominant types: plasmonic localized heating, non-radiative relaxation, and thermal vibration in molecules, as summarized in Fig. 3. The porous, fibrous structure of textiles influences light scattering and heat distribution, while the air layer trapped in the superhydrophobic microstructure acts as a thermal insulator, enhancing localized heating though potentially introducing light scattering trade-offs [83]. This section delineates these mechanisms within the textile context.

### Plasmonic Localized Heating

Plasmonic heating arises from collective oscillation of free electrons in metallic nanostructures upon light interaction, known as surface plasmon resonance (SPR). When incident photon frequency matches the electron oscillation frequency, strong resonance occurs, leading to intense light absorption and nanoscale field confinement [84, 85]. For spherical nanoparticles, the peak resonance wavelength (λ_max_) is approximated by [86, 87]:

7 $$\lambda_{\max } = \lambda_{{\mathrm{p}}} \sqrt {2\varepsilon_{{\mathrm{m}}} + 1}$$

where *λ*_p_ is the plasma wavelength and *ε*_m_ is the dielectric constant of the surrounding medium. This tunability is advantageous; spherical gold nanoparticles absorb in the visible range (~ 520 nm), while gold nanorods can be engineered for near-infrared absorption (~ 700–1100 nm) by adjusting their aspect ratio [88, 89]. Absorbed energy is rapidly converted to heat via electron–electron and electron–phonon scattering [90–92].

**Fig. 4:**
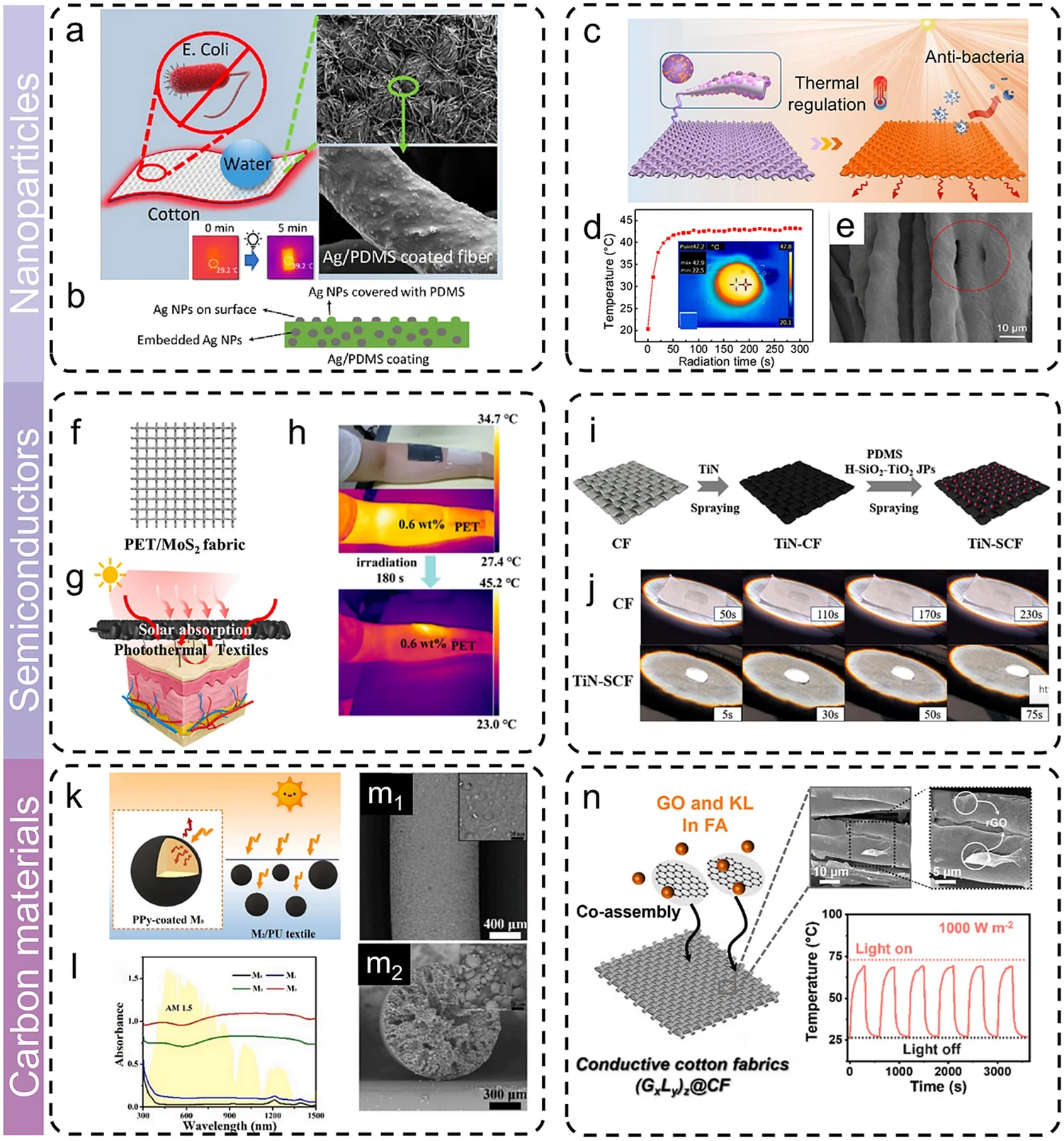
Representative examples of three classes of photothermal materials applied in photothermal superhydrophobic textiles. **a** Schematic diagram and SEM micrographs of the multifunctional Ag/PDMS-coated cotton fabric [93]. **b** Developing rough Ag/PDMS coatings on fibers. Reproduced with permission [93]. Copyright 2023, ACS. **c** Schematic diagram of the structure and functionality of the hybrid P(MA-co-MA_300_-co-EGMA)/Au nanogel coated cotton fabric. [94]. **d** Temperature evolution of CHN-20 with continuous visible light illumination [94]. **e** FE-SEM images of CHN-20. Reproduced with permission [94]. Copyright 2025, Elsevier. **f** Schematic diagram of PET/MoS_2_ hybrid fibers [104]. **g** PET/MoS_2_ fabric’s potential applications in smart textiles for personal thermal management [104]. **h** Digital and IR thermal images of human arms wrapped with pristine PET fabric and 0.6 wt% PET/MoS_2_ fabric, both exposed to IR therapeutic lamp irradiation. Reproduced with permission [104]. Copyright 2025, Elsevier. **i** Fabrication process of the TiN-SCF [105]. **j** Schematic of photothermal deicing of different fabrics. Reproduced with permission [105]. Copyright 2025, ACS. **k** Schematic illustration of the enhancement in photothermal efficiency of M_2_/PU textile [106]. **l** UV–Vis absorption spectra of micro-PCMs and polypyrrole (PPy), and the AM 1.5 solar spectrum is provided as a yellow background for reference [106]. **m**_**1**_, **m**_**2**_ Surface morphology of 70% M_2_/PU fiber. Reproduced with permission [106]. Copyright 2024, Elsevier. **n** SEM images and photothermal performance of (*G*_*x*_*L*_*y*_)_*z*_@CF fabrics. Reproduced with permission [107]. Copyright 2026, Elsevier

This mechanism is effectively leveraged in textiles, as demonstrated in Fig. 4a, b. Ag/PDMS-coated cotton fabric uses the LSPR of Ag nanoparticles for efficient photothermal heating, with the dense nanoparticle packing on fiber surfaces increasing absorption cross section and enabling applications such as UV protection and antibacterial activity [93].

Further extending this concept, Fig. 4c–e shows cotton fabrics cross-linked with hybrid P(MA-co-MA_300_-co-EGMA)/Au nanogels [94]. Under visible light, the fabric (CHN-20) rapidly heats from 20.4 to 43.0 °C within 50 s (Fig. 4d), demonstrating efficient photothermal conversion. The densely packed hybrid nanogels on the cotton surface (Fig. 4e) maximize light absorption while minimizing scattering losses [95, 96].

### Non-radiative Relaxation

Non-radiative relaxation is the dominant photothermal mechanism in semiconductors, including metal–organic frameworks, transition metal dichalcogenides (e.g., MoS_2_, WS_2_), and metallic ceramics such as TiN [97, 98]. When a semiconductor absorbs a photon with energy ≥ its bandgap (*E*_g_), an electron is excited from the valence to the conduction band, generating an electron–hole pair [99, 100]. The bandgap correlates with the cutoff wavelength (*λ*_c_) via [101, 102]:

8 $$E_{{\mathrm{g}}} = \frac{hc}{{\lambda_{{\mathrm{c}}} }}$$

where *h* is Planck’s constant and c is the speed of light. Narrow-bandgap semiconductors like TiN exhibit broad absorption across visible to NIR range. The photogenerated carriers undergo rapid non-radiative recombination, converting excitation energy into lattice vibrations and bulk heating [103].

A representative textile implementation is the PET/MoS_2_ hybrid fiber system (Fig. 4f–h) [104]. MoS_2_ nanosheets uniformly deposited on PET fibers achieve a ~ 10 °C temperature increase under infrared irradiation (Fig. 4h), validating efficient photothermal conversion for personal thermal management. Expanding the material palette, Fig. 4i, j presents a TiN-based multi-layer textile (TiN-SCF) constructed by sequentially spraying TiN nanoparticles, PDMS binder, and hydrophobic SiO_2_–TiO_2_ Janus particles onto cotton [105]. Under 1.0 sun irradiation, ice on TiN-SCF melts completely in ~ 50 s (Fig. 4j), significantly faster than controls. This accelerated deicing arises from synergistic photothermal heating at the ice–fabric interface and the superhydrophobic microstructure minimizing thermal resistance.

### Thermal Vibration in Molecules

This mechanism leverages π-conjugated systems in carbon-based nanomaterials and conjugated polymers (e.g., graphene, CNTs, PPy) [108–110]. Photon absorption excites electrons from HOMO to LUMO [111, 112], followed by ultrafast non-radiative relaxation via electron–phonon coupling, generating heat through increased atomic vibrations [113–115]. Unlike plasmonic heating (requiring specific resonances) or semiconductor bandgap transitions (requiring energy thresholds), molecular vibration heating operates through continuous energy states, enabling broadband solar absorption [108, 109].

Figure 4k–m illustrates a smart textile where PPy-coated micro-PCMs are embedded in polyurethane fibers [107]. The PPy coating provides broad spectral absorption (Fig. 4l), while the PCM core enables thermal energy storage, achieving photothermal storage efficiency *η* = 94.03% and a cooling delay exceeding 1100 s. Figure 4n demonstrates a graphene–lignin composite (*G*_*x*_*L*_*y*_)_*z*_@CF that reaches 70 °C under 1.0 sun irradiation [106]. The co-assembly of graphene oxide and lignin creates a stable conductive network, with synergistic enhancement of photothermal performance and mechanical durability. These examples demonstrate that molecular vibration mechanisms provide a versatile foundation for designing photothermal textiles with tunable thermal regulation [116].

## Interplay Between Superhydrophobicity and Photothermal Functionality

**Fig. 5:**
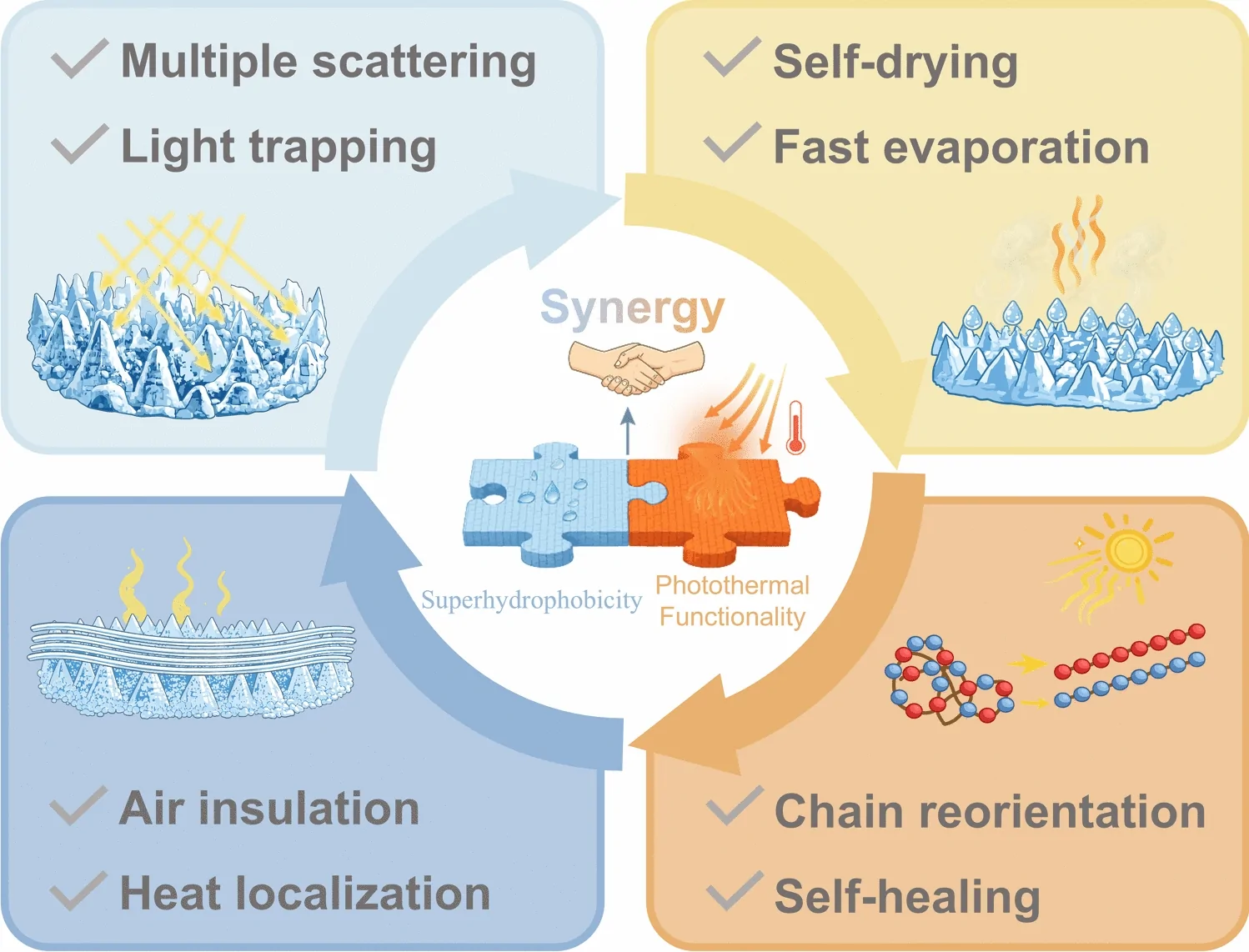
Synergistic co-design of photothermal functionality and superhydrophobicity in textiles. Four synergistic mechanisms are illustrated: (top left) light-trapping hierarchical structures enhancing photothermal absorption; (bottom left) air-pocket-enabled thermal insulation minimizing heat loss; (top right) photothermal heating driving rapid evaporation to sustain dry superhydrophobicity; (bottom right) photothermal heating enabling autonomous chemical self-healing via restoration of low-surface-energy chain orientation

The true innovation in advanced textiles lies in the synergistic interplay between superhydrophobicity and photothermal activity (Fig. 5). This mutual reinforcement creates a system greater than the sum of its parts. The relationship is bidirectional: the micro-/nano-architecture of a superhydrophobic surface enhances photothermal conversion, while the generated heat maintains and restores water repellency. This section deconstructs this symbiotic relationship.

### How Superhydrophobicity Enhances Photothermal Functionality

The hierarchical roughness defining superhydrophobic surfaces is ideally suited for maximizing light absorption and minimizing heat loss, operating through two primary mechanisms: enhanced light trapping and superior thermal insulation.

First, the micro-/nano-scale texture acts as an efficient optical trap. Incident photons undergo repeated reflection, refraction, and scattering within pores and surface asperities, increasing the effective optical path length and providing more opportunities for photothermal agents to harvest light energy [117–121]. This light-trapping effect can be described using a Beer–Lambert-type effective path length model [122, 123]:

9 $$A \approx 1 - e^{{ - \alpha L_{{{\mathrm{eff}}}} }}$$

where *α* is the effective absorption coefficient and *L*_eff_ is the effective optical path length. Compared with a smooth coating, hierarchical surfaces increase *L*_eff_ through multiple scattering events. As *L*_eff_ increases, ${\mathrm{e}}^{-\upalpha {L}_{\mathrm{e}\mathrm{f}\mathrm{f}}}$ decreases, leading to higher light absorption. This explains why photothermal superhydrophobic textiles with micro-/nano-graded structures achieve high solar absorptance (95–98%), even when using similar photothermal materials as flat substrates [124–128]. The same architecture that traps air for water repellency also traps light for enhanced photothermal conversion.

Second, the air pockets responsible for the Cassie–Baxter state provide thermal insulation. Since air has much lower thermal conductivity than solids and liquids, entrapped air within micro-/nanostructures suppresses heat dissipation from the photothermal layer [120, 125, 129–131]. The effective thermal conductivity of this porous composite can be approximated as:

10 $$k_{{{\mathrm{eff}}}} \approx \varphi_{{\mathrm{s}}} k_{{\mathrm{s}}} + \varphi_{{\mathrm{a}}} k_{{{\mathrm{air}}}}$$

where *φ*_s_ and *φ*_a_ are the volume fractions of solid and air, and *k*_s_ and *k*_air_ are their thermal conductivities. Increasing the trapped air fraction decreases *k*_eff_ and weakens conductive heat loss. The heat loss process follows the thermal resistance relation [17]:

11 $$R_{{{\mathrm{th}}}} = \frac{L}{{k_{{{\mathrm{eff}}}} A_{{\mathrm{c}}} }}$$

12 $$q = \frac{\vartriangle T}{{R_{{{\mathrm{th}}}} }}$$

where *R*_th_ is thermal resistance, *L* is the insulating layer thickness, and *A*_c_ is heat transfer area. A lower *k*_eff_ increases *R*_th_, reducing heat loss from the photothermal surface. This “heat-locking” effect has two consequences. First, it allows the photothermal layer to reach higher equilibrium temperature under the same irradiation. Second, when a cold droplet or ice contacts the surface, the air layer reduces interfacial heat transfer, slowing cooling and prolonging freezing time [120, 121, 126, 129, 132]. Thus, superhydrophobicity enhances photothermal functionality by increasing light harvesting through hierarchical roughness and by lowering thermal conductivity through Cassie state air retention.

### How Photothermal Functionality Enhances Superhydrophobicity

Conversely, photothermal functionality provides a dynamic mechanism to sustain and regenerate the superhydrophobic state through evaporation-induced self-drying, thermally assisted droplet depinning, and chemical self-healing. Superhydrophobic surfaces can be compromised by condensed dew or high humidity, inducing a Cassie-to-Wenzel transition. A photothermal textile counteracts this by converting light into localized heat, promoting water evaporation and accelerating droplet removal before penetration into surface textures [119, 124, 133, 135–140].

From an interfacial transport perspective, photothermal heating induces Marangoni convection inside droplets via surface tension gradients [141–143]:

13 $$\tau_{M} = \nabla_{\Gamma } \gamma = \frac{{{\mathrm{d}}\gamma }}{{{\mathrm{d}}T}}\nabla_{\Gamma } T = \gamma ^{\prime}_{T} \nabla_{\Gamma } T$$

The relative importance of this thermocapillary flow is described by the Marangoni number [141, 143, 144]:

14 $${\mathrm{Ma}} = \frac{{\left| {\frac{{{\mathrm{d}}\gamma }}{{{\mathrm{d}}T}}} \right|\Delta {\mathrm{Ta}}}}{{\mu \alpha_{T} }}$$

A larger $\mathrm{d}T$ from photothermal heating increases $\mathrm{M}\mathrm{a}$, indicating stronger convection. For small sessile droplets, Marangoni convection can dominate over buoyancy-driven flow [143, 145, 146]. This internal circulation enhances interfacial heat and mass transfer, expressed by the Nusselt number [143, 144, 147]:

15 $${\mathrm{Nu}} = \frac{ha}{k}$$

A larger Nu indicates stronger convective heat transfer, accelerating droplet evaporation and self-drying.

Droplet removal is governed by contact line pinning and adhesion [143, 146]:

16 $$F_{{{\mathrm{add}}}} = \pi D_{{\mathrm{w}}} \gamma f\cos (\theta_{R} - \theta_{{\mathrm{A}}} )$$

Photothermal heating facilitates droplet removal by reducing residual water bridges and accelerating evaporation near the contact line, preventing moisture from infiltrating surface textures.

Recent studies show that photothermal superhydrophobic coatings can recover high contact angles after prolonged moisture exposure [126, 135, 138, 140, 148]. However, this synergy has boundary conditions. Excessive heating may induce vapor condensation and Cassie-to-Wenzel transition [149]. Thus, photothermal functionality should be understood as a regulated interfacial transport process within an appropriate thermal window.

Beyond physical water removal, photothermal heating restores superhydrophobicity by activating the reorientation of low-surface-energy molecular chains. Washing or contamination can cause hydrophobic chains to curl inward while polar groups become exposed, increasing surface energy and destroying water repellency [135, 140–143, 150, 151]. The temperature dependence of polymer chain mobility follows Arrhenius-type relaxation [152, 153]:

17 $$\tau (T) = \tau_{0} \exp \left( {\frac{{E_{{\mathrm{a}}} }}{RT}} \right)$$

Increasing surface temperature reduces relaxation time, accelerating hydrophobic segment migration toward the air-facing surface. Near the glass transition region, the WLF equation provides a more accurate description [152]:

18 $$\log \left( {\frac{\tau (T)}{{\tau (T_{{\mathrm{g}}} )}}} \right) = - \frac{{C_{1} \left( {T - T_{{\mathrm{g}}} } \right)}}{{C_{2} + \left( {T - T_{{\mathrm{g}}} } \right)}}$$

where τ(T) is the relaxation time at temperature *T*, *τ*(*T*_g_) is the glass transition temperature, an C_1_, C_2_ are material constants. A small temperature increase near *T*_g_ substantially enhances chain mobility.

The conformational recovery of hydrophobic chains can be quantified by end-to-end distance [154, 155]:

19 $$R_{{\mathrm{e}}} = \left| {{\mathbf{r}}_{{{\mathrm{end}}}} - {\mathbf{r}}_{{{\mathrm{start}}}} } \right|$$

where a larger *R*_e_ indicates extended chain conformation. Molecular dynamics simulations of SPS cotton fabric showed alkane-chain end distances decreased from 18–20 to 15–17 Å after washing, confirming chain curling. After photothermal self-healing, the distribution became broader and more homogeneous, indicating chain reorientation [154].

To connect molecular-level recovery with macroscopic wettability, the WCA recovery efficiency is defined as [150, 154, 156]:

20 $$\eta_{\theta } = \frac{{\theta_{{{\mathrm{healed}}}} - \theta_{{{\mathrm{damaged}}}} }}{{\theta_{{{\mathrm{initial}}}} - \theta_{{{\mathrm{damaged}}}} }} \times 100\%$$

where *η*_θ_ is the recovery efficiency, *θ*_initial_ is the initial WCA, *θ*_damaged_ is the WCA after damage, and *θ*_healed_ is the WCA after self-healing. For example, SPS cotton fabric recovered nearly 100% water repellency under 30 mW cm^−2^ irradiation, whereas non-photothermal fabric recovered only ~ 71.2% [154].

Thus, photothermal self-healing is a thermally activated molecular reorientation process. Photothermal conversion raises local temperature into the chain mobility window, shortening relaxation time and driving nonpolar chains to reorient toward the air interface, restoring low surface energy without external heating.

Although several studies have demonstrated photothermal-assisted self-drying or self-healing, their time efficiency and energy input are still rarely reported in a standardized manner. Available results suggest that the recovery time can vary from several minutes to several hours depending on coating chemistry, damage mode, and irradiation conditions. For example, SPS cotton recovered its WCA to approximately 150° within 20 min under 0.3 sun irradiation and became completely dry within about 15 min under outdoor sunlight of approximately 0.42 sun [154]. A fluorine-free photothermal superhydrophobic fabric recovered to WCA = 160.8° after O_2_ plasma treatment and 12 h of room-temperature healing [138], while TiN-coated cotton fabrics were evaluated after 30 min of xenon lamp irradiation for photothermal self-healing [159]. More recently, sunlight-induced self-healing within 5 min has also been reported for plasma- and scratch-damaged superhydrophobic coatings [160]. For rapid self-drying, low-adhesion superhydrophobic surfaces can remove impacting droplets on the millisecond scale, as reflected by a 14.3 ms solid–liquid contact duration for a 10 μL droplet on a robust photothermal superhydrophobic coating [126]. However, droplet bouncing should be distinguished from photothermal evaporation of residual or trapped moisture. Future studies should therefore report light intensity, illuminated area, water volume, drying time, WCA recovery time, recovery efficiency, and energy input more explicitly, so that photothermal self-drying and self-healing can be quantitatively compared across different systems.

### Boundary Conditions and Potential Negative Effects of Photothermal Heating

Despite the beneficial role of photothermal functionality in maintaining and restoring superhydrophobicity, this effect should not be regarded as unlimited positive feedback. Instead, photothermal regulation operates within a distinct temperature window. When the surface temperature is below the chain mobility threshold of the low-surface-energy coating, molecular segments cannot effectively rearrange, leading to incomplete wettability recovery. For example, in the SPS cotton fabric, the PA coating has a *T*_g_ of approximately 39 °C. Under 0.1 and 0.2 sun irradiation, the surface temperatures reached only 32 and 38 °C, respectively, which were insufficient to fully activate molecular chain reorientation. In contrast, under 0.3 sun irradiation, the surface temperature exceeded this threshold, enabling the WCA to recover to approximately 150° [154]. Similar photothermal-triggered self-healing behavior has also been reported in coatings using PDA, TiN, MXene, or carbon-based absorbers [138, 157–160].

However, higher temperature is not always beneficial. Excessive localized heating or overly intense evaporation may promote vapor condensation within surface textures, disturb the Cassie-Baxter state, and even trigger a Cassie-to-Wenzel transition, thereby increasing liquid adhesion and weakening superhydrophobicity [149]. Moreover, long-term overheating can accelerate polymer photo-oxidation, thermal aging, coating softening, or migration of low-surface-energy components, ultimately destabilize the hierarchical surface texture, and reduce coating durability [152, 153, 161]. Therefore, the design of photothermal superhydrophobic textiles should not simply pursue maximum light absorption or the highest surface temperature. An ideal system should generate sufficient heat to activate droplet removal and molecular chain reorientation while remaining below the range where interfacial condensation, polymer softening, or coating degradation becomes significant.

Overall, the interplay between superhydrophobicity and photothermal functionality is a regulated coupling, not a simple positive feedback loop. Hierarchical roughness and trapped air enhance photothermal performance by extending optical path length and reducing heat loss. Conversely, photothermal heating sustains superhydrophobicity by accelerating evaporation, promoting droplet depinning, and activating molecular reorientation. However, these effects operate within boundary conditions: excessive heating may induce Cassie-to-Wenzel transition, while insufficient heating leads to incomplete recovery. Therefore, rational design should optimize optical trapping, heat retention, droplet dynamics, and molecular chain mobility within an appropriate working window.

## Photothermal Superhydrophobic Textiles: Development and Classification

**Fig. 6:**
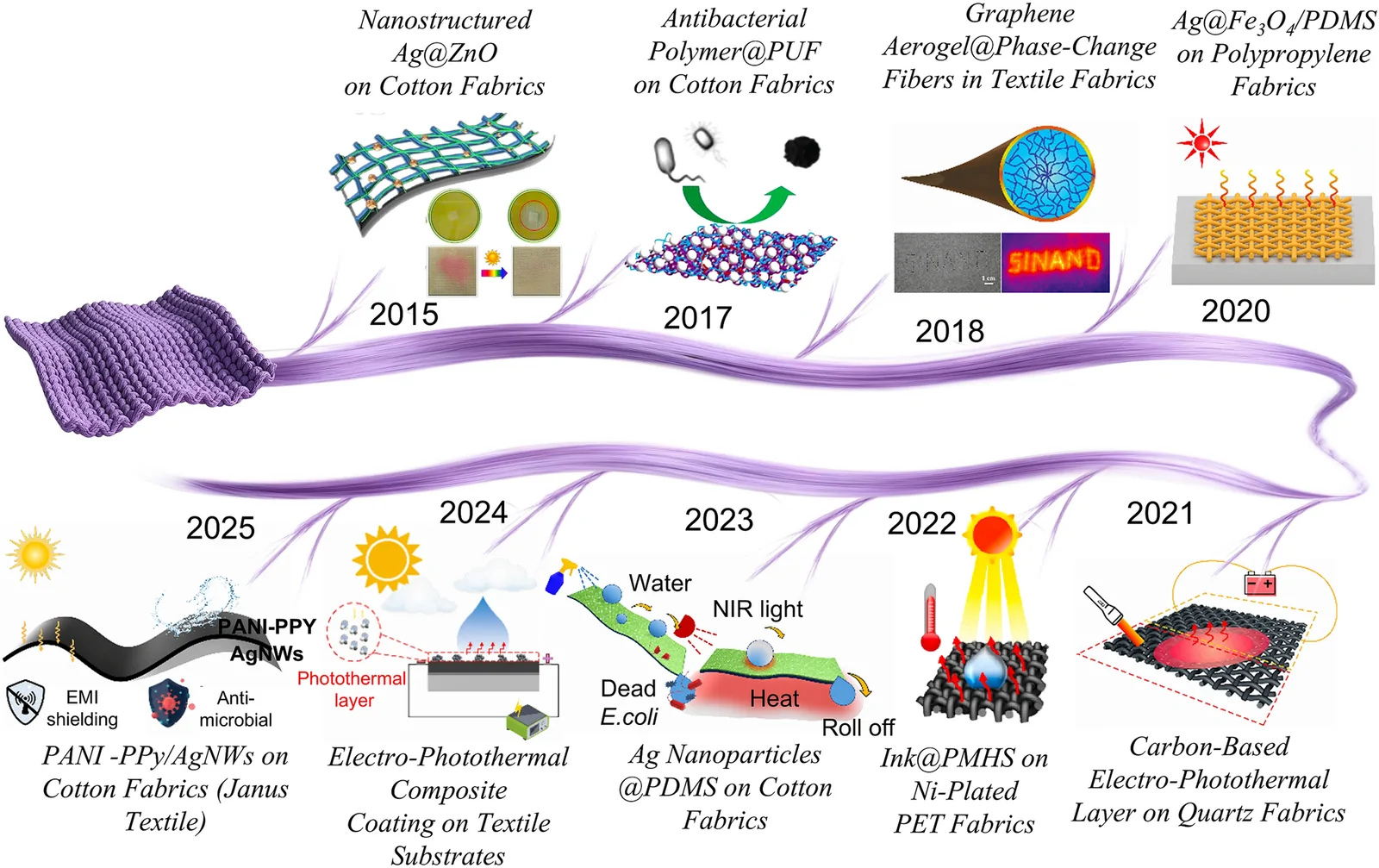
A decade of progress in photothermal superhydrophobic textiles (2015–2025). Ag@ZnO/cotton. Reproduced with permission [162]. Copyright 2015, ACS. pH-responsive copolymer and PUF NPs/cotton. Reproduced with permission [163]. Copyright 2017, ACS. Graphene-aerogel/phase-change fiber. Reproduced with permission [164]. Copyright 2018, Wiley. Ag@Fe_3_O_4_/PDMS/PP. Reproduced with permission [169]. Copyright 2020, Elsevier. C/quartz fabric. Reproduced with permission [166]. Copyright 2021, Elsevier. Ni–P/Chinese ink/PET. Reproduced with permission [165]. Copyright 2022, Elsevier. Ag/PDMS/cotton. Reproduced with permission [93]. Copyright 2023, ACS. Biochar/SiO_2_/SEBS-nylon. Reproduced with permission [167]. Copyright 2024, Wiley. PANI (polyaniline)-PPy/AgNWs/cotton. Reproduced with permission. Reproduced with permission [168]. Copyright 2025, ACS

The past decade (2015–2025) has witnessed a remarkable evolution in photothermal superhydrophobic textiles, transforming them from laboratory curiosities into sophisticated multi-functional platforms. As chronologically summarized in Fig. 6, this progression reveals clear paradigm shifts: from passive water repellency to actively responsive systems for thermal management, energy conversion, and environmental adaptation. This integrated section provides a concise milestone-oriented overview and then transitions to a substrate-based classification, avoiding the repetition inherent in separate chronological and categorical discussions.

### Development Milestones

The inaugural phase (2015) integrated photocatalytic and plasmonic properties for self-cleaning surfaces, exemplified by Ag@ZnO-coated cotton fabrics via bio-inspired mineralization [162]. By 2017, stimuli responsiveness emerged through antibacterial polymer@PUF on cotton, enabling pH-triggered wettability switching for oil–water separation [163]. A pivotal leap occurred in 2018 with graphene aerogel@phase-change fibers, introducing energy management by combining broadband light absorption with latent heat storage (93.5 J g^−1^) [164].

The 2020–2022 period focused on harsh environment performance. The Ag@PDMS on polypropylene fabrics (S-PPF) combined superhydrophobicity with Joule heating, photothermal effects, and EMI shielding [165]. Carbon-based electro-photothermal layers on quartz fabrics (QF-CF) reached 49.5 °C under solar simulation and 87.2 °C at 3.0 V [166]. The ink@PMHS on Ni-plated PET fabrics (PMHS-Ink/Ni–P/PET) achieved delayed icing of 507 s at − 14 °C using cost-effective Chinese ink146 [165]. In 2024, the electro-photothermal composite coating (EPS) demonstrated robust all-weather reliability with dual-mode heating (51.8 °C under 1.0 sun; 83.2 °C at 3.0 V) [167]. Most recently, integrated multifunctionality has been emphasized through systems such as Ag@PDMS-coated cotton and PANI-PPy/AgNWs/cotton textiles, combining photothermal conversion, antibacterial protection, UV shielding, and electromagnetic attenuation [93, 168].

Collectively, the trajectory mapped in Fig. 6 reveals distinct phases: foundational multifunctionality (2015), stimuli responsiveness (2017), energy management (2018), harsh environment robustness (2020–2022), all-weather reliability (2024), and integrated smart functionality (2023–2025). To avoid repetition, detailed material compositions, fabrication strategies, and application-specific mechanisms are discussed in the following substrate-based classification and subsequent application sections.

### Classification by Substrate Type

The design of photothermal superhydrophobic textiles is strongly governed by the intrinsic properties of the textile substrate. Unlike rigid substrates, fabrics are flexible, porous, deformable, and chemically heterogeneous assemblies of fibers and yarns. Their fiber chemistry, surface functional groups, pore structure, thermal stability, mechanical strength, moisture behavior, comfort, and environmental footprint all influence the construction of superhydrophobic micro-/nanostructures and the integration of photothermal components. Therefore, substrate selection should not be regarded as a passive background factor, but as a key design variable that determines modification strategy, durability, functionality, and application suitability.

**Fig. 7:**
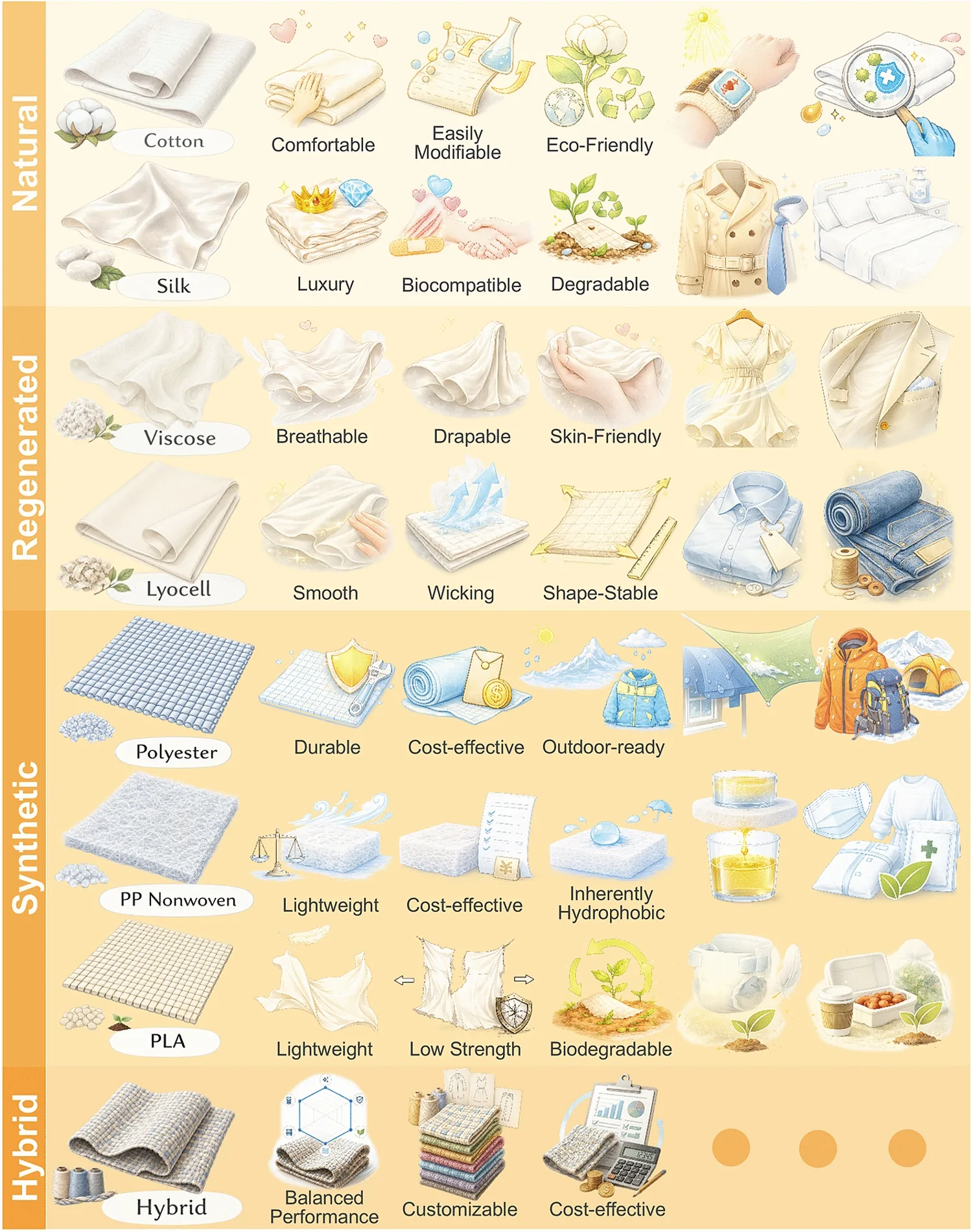
Textile substrates for photothermal superhydrophobic modification: intrinsic properties and representative application scenarios. PLA: polylactic acid‌. PP: polypropylene

As illustrated in Fig. 7, textile substrates for photothermal superhydrophobic modification can be broadly categorized into four groups: natural fibers, regenerated fibers, synthetic fibers, and hybrid fabrics. Natural fibers, such as cotton and silk, provide comfort, biocompatibility, and abundant surface functional groups. Regenerated fibers, represented by viscose and lyocell, combine cellulose-based chemistry with industrially controlled fiber formation, offering softness, drapability, and skin contact comfort. Synthetic fibers, including polyester, polypropylene, PLA, and related polymeric fibers, provide tunable mechanical properties, low cost, durability, and scalable manufacturing. Hybrid fabrics, including blended fibers, composite yarns, laminated textiles, and multi-component fabrics, are widely used in practical textile products because they can balance comfort, strength, elasticity, cost, and durability. This classification provides a more realistic framework for connecting substrate characteristics with photothermal superhydrophobic design.

#### Natural Fibers

Natural fibers remain the most widely studied substrates for photothermal superhydrophobic textiles due to their wearing comfort, breathability, biocompatibility, and environmental benefits. More importantly, their abundant surface functional groups facilitate the deposition of photothermal materials and the construction of low-surface-energy coatings.

**Fig. 8:**
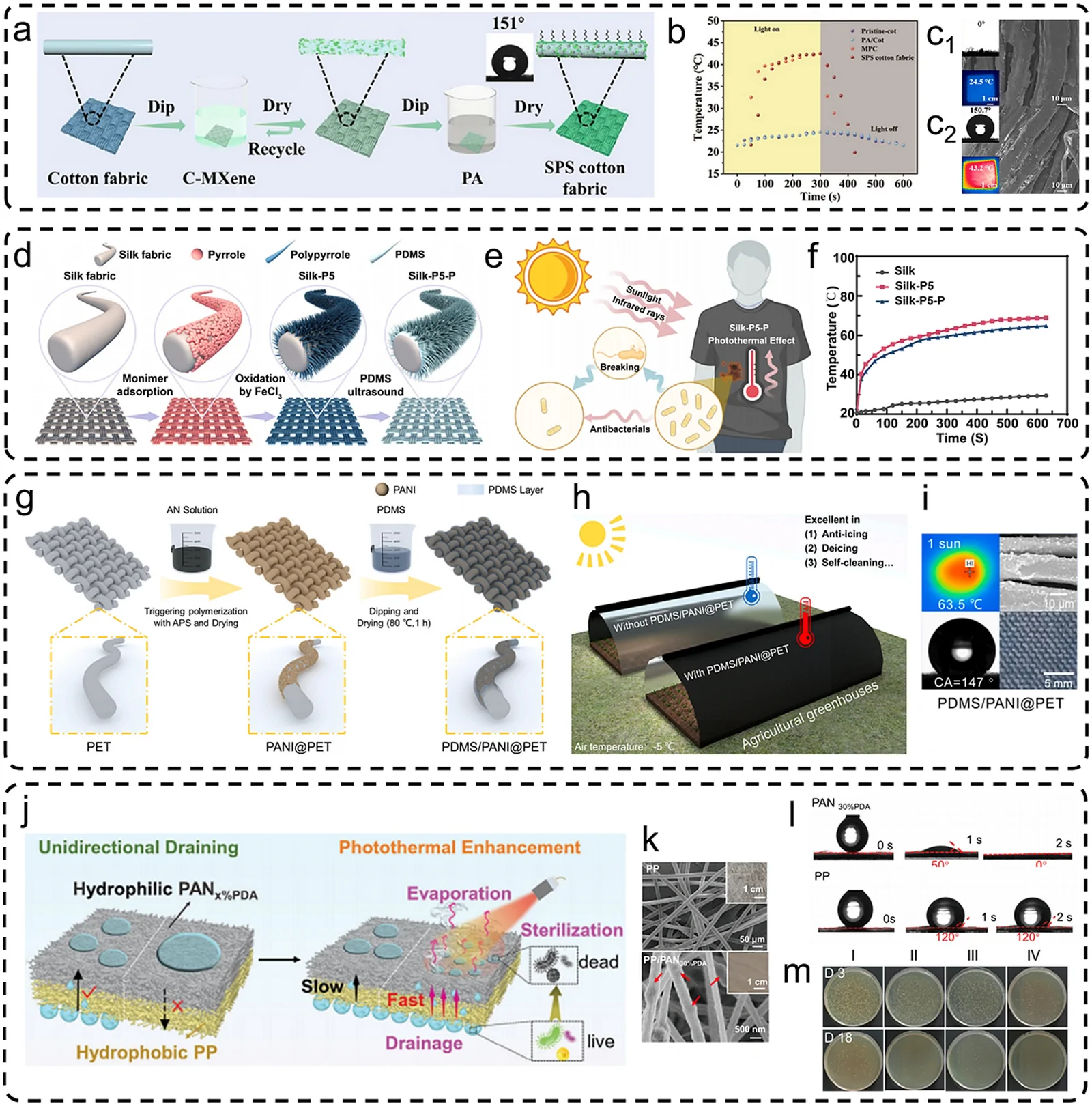
Representative research examples of photothermal superhydrophobic textiles based on different fabric substrates. **a** Fabrication process of the SPS cotton fabric; the insets indicate the corresponding water droplet image [154]. **b** Variation of surface temperature for cotton, PA/cotton, MXene/PA/cotton (MPC), and SPS cotton fabrics [154]. **c1** SEM image of the washed SPS cotton fabric in the condition of air drying; the insets indicate the corresponding water droplet image (left top) and surface temperature (left bottom), respectively [154]. **c2** SEM image of the washed SPS cotton fabric under 0.3 sun irradiation for 20 min; the insets indicate the corresponding water droplet image (left top) and surface temperature (left bottom), respectively. Reproduced with permission [154]. Copyright 2025, ACS. **d** Method for formulating Silk-PPy-PDMS of different components [171]. **e** Schematic of the photothermal antibacterial mechanism [171]. **f** Comparative temperature profiles of pristine silk, Silk-P5, and Silk-P5-P over the 10-min irradiation period at 1.0 W cm^−2^ (808 nm) in wet environment. Reproduced with permission [171]. Copyright 2026, Springer Nature. **g** Schematic diagram for the process of preparation of PDMS/PANI@PET superhydrophobic photothermal fabrics [172]. **h** Application diagram of PDMS/PANI@PET in agriculture [172]. **i** Surface temperature distribution, SEM image, water contact angle, and TEM image of PDMS/PANI@PET. Reproduced with permission [172]. Copyright 2025, Elsevier. **j** Schematic illustration of Janus PP/PAN_X%PDA_ fibrous membrane [173]. **k** SEM images of PP and PP/PAN_30%PDA_. **l** Water contact angle of PP and PP/PAN_30%PDA_ [173]. **m** Corresponding pictures of colonies of S. aureus derived from wound tissues at days 3 and 18. (I, Control; II, Bandage; III, PP/PAN_30%PDA_; IV, PP/PAN_30%PDA_ and laser; *p < 0.05, **p < 0.01, ***p < 0.001; *n* = 3). Reproduced with permission [173]. Copyright 2024, Wiley

Cotton is one of the most representative natural fiber substrates. Its cellulose backbone contains abundant hydroxyl groups, enabling surface modification through hydrogen bonding, silane chemistry, polydopamine adhesion, polymer coating, and nanoparticle deposition. This makes cotton highly suitable for constructing hierarchical roughness and anchoring photothermal components. As shown in Fig. 8a–c, a superhydrophobic photothermal self-healing cotton fabric was fabricated by sequentially depositing a cellulose nanocrystal-MXene photothermal layer and a polyacrylate hydrophobic layer [154]. In this system, the cotton substrate provides a flexible and hydrophilic scaffold for coating assembly, while C-MXene contributes light-harvesting capability and the polyacrylate layer provides water repellency. After laundering, the swollen cotton fibers and rearranged hydrophobic chains may cause temporary loss of superhydrophobicity. Under mild solar irradiation, photothermal heating promotes moisture removal and the reorientation of nonpolar alkane chains in the polyacrylate coating, restoring surface water repellency with nearly 100% efficiency [154].

Silk is another important natural fiber, valued for its smooth texture, high wearing comfort, biocompatibility, and premium tactile properties [170]. As a protein fiber, silk differs from cellulose fibers in both chemical composition and surface characteristics, providing a distinct platform for wearable and biomedical textile applications. A representative example is Silk-PPy-PDMS, where PPy forms hierarchical nanostructures on silk fibers and serves as the photothermal and conductive component, while PDMS provides low surface energy and water repellency [171]. As shown in Fig. 8d–f, this design transforms silk into a multifunctional textile integrating superhydrophobic self-cleaning, photothermal antibacterial activity, and electromagnetic interference shielding. Such systems are particularly suitable for skin contact protective textiles, medical fabrics, and smart wearable devices.

Despite these advantages, natural fibers also face durability challenges. Their hydrophilicity and swelling behavior may weaken coating stability under washing, high humidity, or repeated wetting/drying cycles. In addition, the environmental benefit of natural fibers may be reduced when coated with fluorinated modifiers, synthetic polymers, or metallic nanoparticles. Therefore, future natural fiber-based photothermal superhydrophobic textiles should emphasize fluorine-free surface chemistry, strong interfacial anchoring, reduced particle shedding, and environmentally compatible end-of-life treatment.

#### Regenerated Fibers

Regenerated fibers, such as viscose and lyocell, are man-made cellulose fibers produced from natural cellulose resources. They occupy an intermediate position between natural and synthetic fibers: their backbone is cellulose-based, but fiber formation relies on industrial regeneration. Compared with cotton, regenerated fibers generally show smoother surfaces, soft hand feel, good drapability, and favorable moisture management behavior. Lyocell, in particular, is valued for its dimensional stability and cleaner solvent-spinning process relative to conventional viscose production.

From the perspective of photothermal superhydrophobic design, regenerated cellulose fibers are promising substrates because they retain abundant hydroxyl groups, which support surface anchoring of functional coatings. Their softness, breathability, and skin contact comfort make them attractive for wearable photothermal textiles, including apparel, linings, bedding, and medical or hygiene-related fabrics. Their cellulose-based composition also offers opportunities for developing more sustainable superhydrophobic textile platforms.

However, regenerated fibers also introduce specific challenges. Their moisture absorption and swelling behavior may disturb the Cassie state and weaken coating–fiber interfaces under humid or washing conditions. The smoother surface of lyocell or viscose may also require additional roughness construction to achieve stable superhydrophobicity. Therefore, robust interfacial engineering—such as covalent anchoring, hydrogen-bonded networks, or bioinspired adhesive layers—is necessary to maintain coating stability during bending, laundering, and long-term service.

At present, direct research examples of regenerated fibers in photothermal superhydrophobic textiles remain limited. This contrasts with their wide use in real textile products and highlights an important research gap. Considering their comfort, cellulose-based chemistry, and increasing relevance in sustainable textile manufacturing, regenerated fibers deserve more attention as next-generation substrates for breathable, skin-friendly, and environmentally conscious photothermal superhydrophobic fabrics.

#### Synthetic Fibers

Synthetic fibers are widely used in functional textiles due to their low cost, tunable mechanical properties, dimensional stability, durability, and compatibility with large-scale manufacturing. Compared with natural and regenerated fibers, synthetic fibers generally exhibit lower moisture absorption and better resistance to deformation, microbial degradation, and outdoor environments, making them attractive for protective covers, outdoor fabrics, filtration materials, and industrial textiles.

Polyester is one of the most important synthetic substrates for photothermal superhydrophobic textiles. As shown in Fig. 8g–i, PDMS/PANI@PET fabric was prepared by in situ polymerization of PANI on PET followed by PDMS coating [172]. PANI provides photothermal conversion and nanoscale roughness, while PDMS contributes low surface energy and water repellency. The resulting fabric maintains the flexibility and mechanical stability of PET while gaining photothermal and superhydrophobic functions, making it suitable for outdoor protective textiles such as agricultural greenhouse covers.

Polypropylene nonwoven fabrics are another important synthetic substrate, especially for disposable medical, filtration, and protective products. Their intrinsic hydrophobicity, low density, and low cost are advantageous for large-scale fabrication. As shown in Fig. 8j–m, a Janus PP/PANx%PDA membrane was constructed by electrospinning a PDA-containing PAN nanofiber layer onto a PP nonwoven substrate [173]. The asymmetric structure enables unidirectional liquid transport, while PDA provides photothermal responsiveness and antibacterial function.

PLA represents a special synthetic fiber because it is bio-based and biodegradable. It combines polymer processability with improved sustainability potential, making it attractive for disposable and environmentally friendly textile products. However, PLA generally has lower heat resistance and weaker mechanical robustness than many petroleum-based synthetic fibers, which may limit its stability under high photothermal temperatures or repeated mechanical stress.

Overall, synthetic fibers are well suited for applications requiring mechanical robustness, low cost, outdoor durability, and scalable production. Their main limitation lies in sustainability. Petroleum-based fibers such as PET and PP raise concerns regarding microplastic release and end-of-life treatment. Future research should therefore combine the durability of synthetic fibers with recyclable coating designs, bio-based polymers, safer photothermal fillers, and reduced environmental release.

#### Hybrid Fabrics

Hybrid fabrics, including blended fibers, composite yarns, laminated textiles, and fabrics constructed from multiple fiber types, are extremely common in real textile products. In practical clothing and technical textiles, blending is widely used to balance comfort, strength, elasticity, cost, breathability, and appearance. For example, cotton/polyester blends combine the comfort of cotton with the dimensional stability and durability of polyester, while elastane-containing blends provide stretchability for sportswear and wearable devices.

For photothermal superhydrophobic textiles, hybrid fabrics offer a promising route to balance multiple performance requirements at the substrate level. Single-fiber substrates often have clear strengths and weaknesses: cotton is comfortable and easy to modify but absorbs moisture; polyester is durable but less reactive toward coating adhesion; PLA is more sustainable but mechanically weaker; silk is comfortable but costly. Hybridization can integrate complementary properties within one textile platform, where one fiber component provides comfort, another contributes strength, and the functional coating adds superhydrophobicity and photothermal conversion.

However, hybrid fabrics also introduce additional design complexity. Different fibers in a blended fabric may have different surface chemistries, swelling behavior, mechanical modulus, and coating affinity. A coating strategy optimized for one fiber type may not uniformly adhere to all components in a hybrid substrate. During washing, bending, abrasion, and thermal cycling, such interfacial mismatch may accelerate coating cracking or uneven performance degradation. Therefore, hybrid substrates require more universal interfacial strategies, such as adhesive primer layers, polymer crosslinking, or mussel-inspired coatings.

Despite their importance in commercial textiles, hybrid fabrics remain rarely explored in photothermal superhydrophobic research. This mismatch between real-world textile use and current research focus deserves attention. Future studies should move beyond idealized single-fiber substrates and consider commercially relevant blended fabrics, because practical clothing, outdoor textiles, and protective fabrics often rely on hybrid structures. Research in this direction would help bridge laboratory demonstrations and real textile applications.

### Perspective on Substrate Selection

The four substrate categories described above demonstrate that fiber selection strongly influences the design logic and application potential of photothermal superhydrophobic textiles. Natural fibers provide comfort, biocompatibility, and accessible surface chemistry; regenerated fibers offer cellulose-based sustainability, softness, and drapability; synthetic fibers contribute durability, cost-effectiveness, and scalable processing; hybrid fabrics enable balanced and customizable textile performance. Each category offers distinct opportunities and faces specific limitations.

Current research remains concentrated mainly on natural fibers such as cotton and silk and synthetic fibers such as PET and PP, as reflected by the representative systems summarized in Fig. 8 [154, 171–173]. In contrast, regenerated and hybrid fibers are widely used in commercial textiles but remain underrepresented in photothermal superhydrophobic studies. Expanding substrate selection toward regenerated cellulose fibers, bio-based synthetic polymers, and commercially relevant blended fabrics will be important for developing smart textiles that are not only highly functional, but also comfortable, durable, scalable, and environmentally responsible.

## Fabrication Approaches for Photothermal Superhydrophobic Textile

**Fig. 9:**
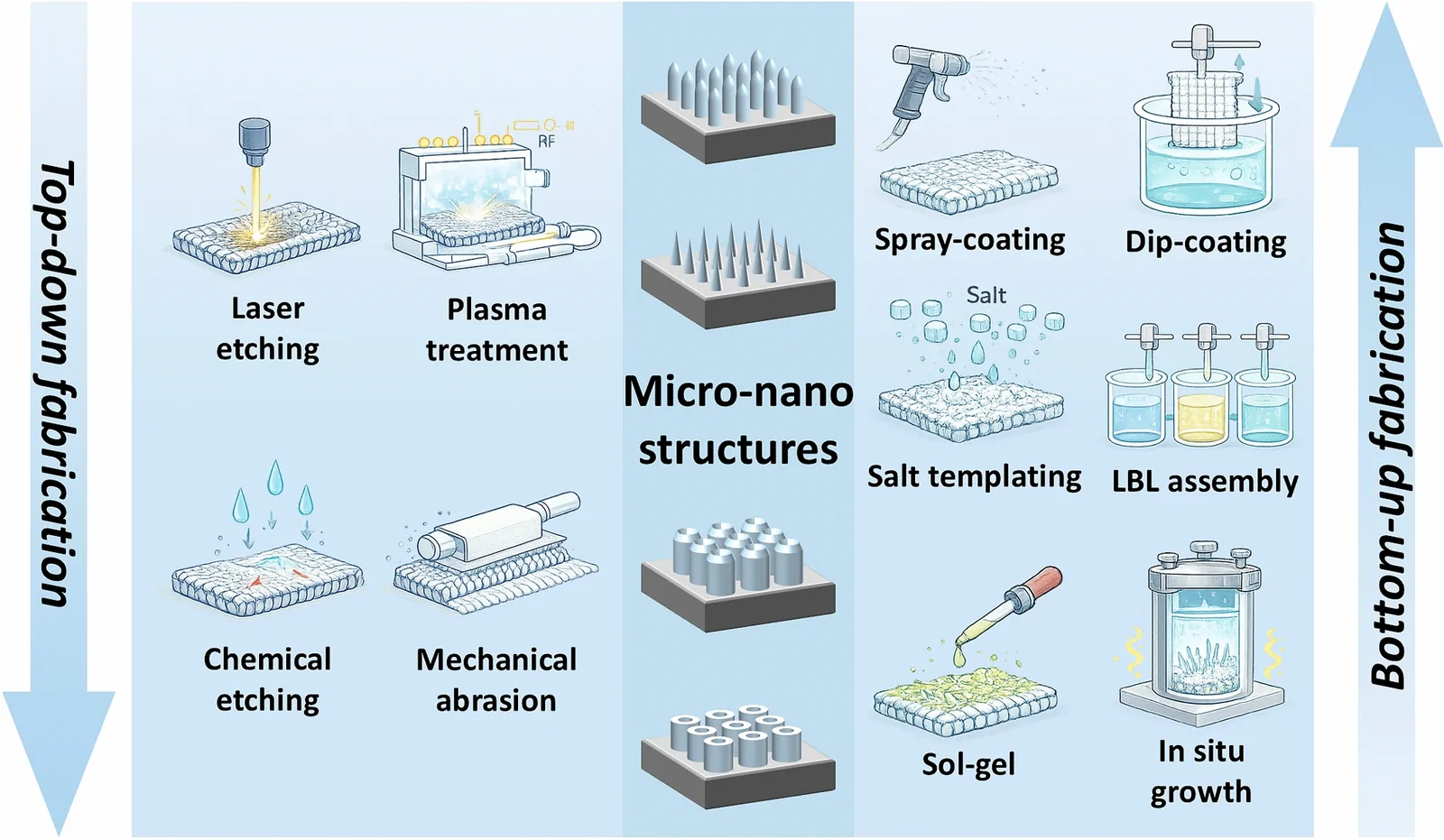
Fabrication strategies for photothermal superhydrophobic textiles: top-down and bottom-up approaches

The successful translation of conceptual designs into functional photothermal superhydrophobic textiles depends on the strategic selection of fabrication methods. These processes must balance creating durable hierarchical surface topography for the Cassie-Baxter state while effectively integrating photothermal nanomaterials. As illustrated in Fig. 9, fabrication routes bifurcate into two complementary paradigms: top-down and bottom-up approaches.

### Top-Down Approaches

Top-down strategies modify existing textile substrates to create micro- and nanoscale surface features, often yielding enhanced durability due to the absence of a discrete coating–substrate interface. However, efficacy varies significantly based on substrate properties [138, 174–176].

**Fig. 10:**
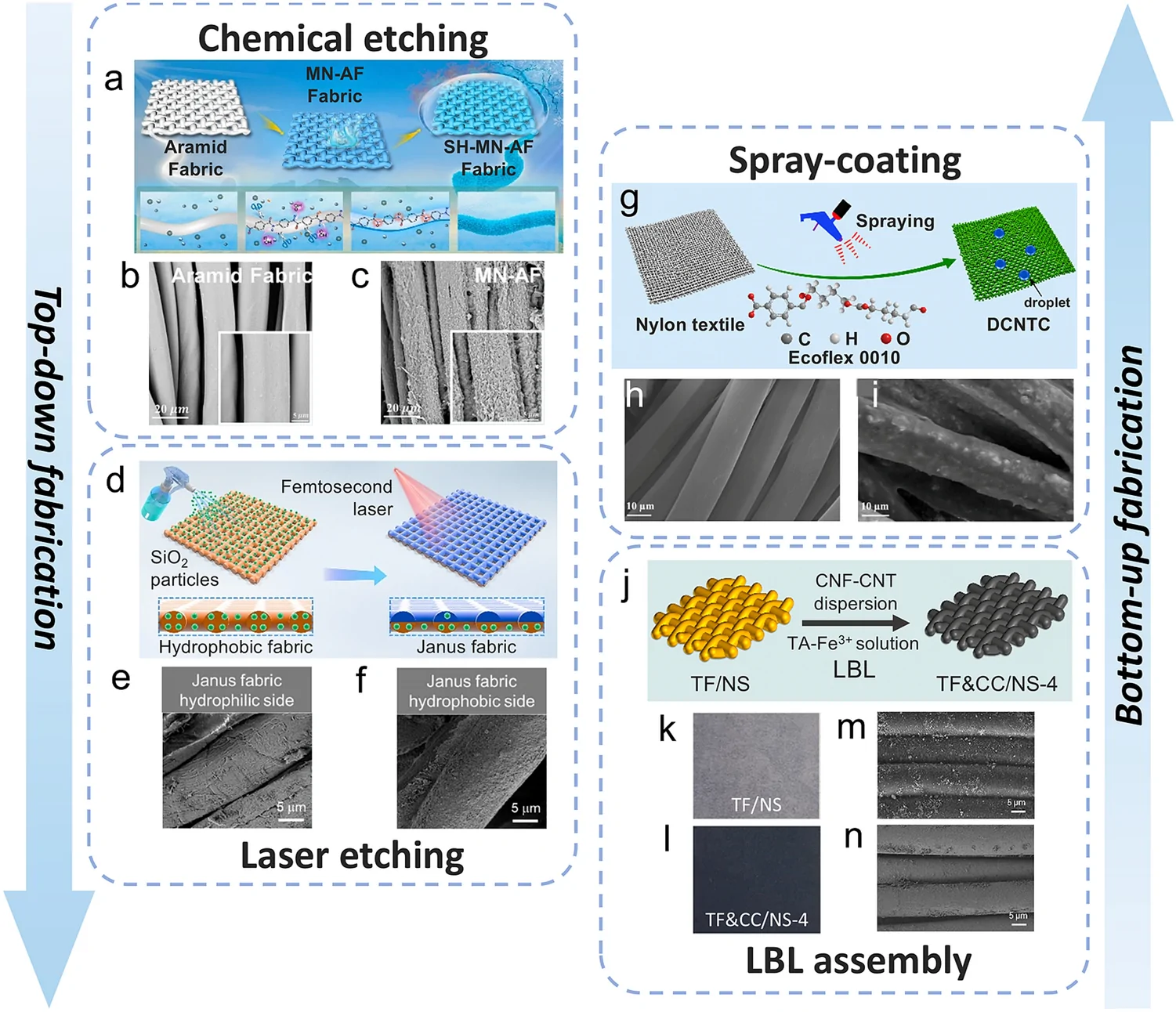
Representative examples of top-down and bottom-up fabrication routes for photothermal superhydrophobic textiles. **a** Schematic illustration of the fabrication process of SH-MN-AF fabric [23]. SEM images of **b** pristine aramid fabric and **c** MN-AF fabric. Reproduced with permission [23]. Copyright 2024, Elsevier. **d** Schematic illustration of the fabrication process of Janus fabrics [177]. Surface morphology characterizations of **e** Janus fabric hydrophilic side and **f** Janus fabric hydrophobic side. Reproduced with permission [177]. Copyright 2025, Elsevier. **g** Schematic illustration of the fabrication [168]. SEM images of **h** the original nylon textile and **i** the dual conductive network textile-based composite (DCNTC). Reproduced with permission [187]. Copyright 2025, Elsevier. **j** Illustration of the assembly process of OTCNS via the LBL assembly approach and super-hydrophobic treatment [188]. Digital photographs of **k** TF/NS and **l** TF and CC/NS-4. SEM images for the surface of **m** TF/NS, **n** TF and CC/NS-4. Reproduced with permission [188]. Copyright 2025, Elsevier

Chemical etching enables functionalization of challenging substrates. For instance, a superhydrophobic anti-icing aramid fabric (SH-MN-AF) was developed via a proton donor-assisted deprotonation-induced roughening (PAIR) method (Fig. 10a) [23]. Using a KOH/DMSO solvent system, this approach created densely packed acicular nanostructures on the fiber surface without compromising core integrity (Fig. 10b, c). The resulting “rough-surface-on-intact-core” architecture achieved an icing delay time of 1920 s—1150% that of pristine aramid fabric.

Laser etching offers unparalleled precision for creating defined surface architectures. Janus fabrics with asymmetric wettability were fabricated by creating a uniformly hydrophobic cotton fabric via SiO_2_ nanoparticle spraying, followed by selective femtosecond laser scanning on one side to remove particles and restore hydrophilicity (Fig. 10d) [177]. The laser-treated side showed exposed smooth fibers (Fig. 10e), while the untreated side-maintained nanoparticle coverage and hydrophobicity (Fig. 10f). This enabled unidirectional liquid transport, effective antifouling, and enhanced thermal comfort.

Plasma treatment occupies a middle ground between chemical and physical approaches, offering gentle yet effective surface activation [178–181]. It creates nanoscale roughness while simultaneously introducing reactive functional groups as anchoring sites for subsequent functionalization [182]. This is particularly valuable for modifying inert polymers like polypropylene, as demonstrated in the successful adhesion of PDMS/Ag nanoparticle layers to plasma-activated PP fabrics [183].

Mechanical abrasion represents the simplest but least controlled method, often causing unacceptable damage to fiber integrity. These examples collectively highlight that top-down methods excel when the substrate itself can be transformed into the functional material, creating systems where surface and bulk properties are intrinsically linked for enhanced durability and performance.

### Bottom-Up Approaches

In contrast to top-down methods, bottom-up approaches construct functionality through the systematic assembly of nanoscale components onto textile substrates, enabling precise engineering of photothermal and superhydrophobic properties [184–186].

Spray-coating offers rapid processing and scalability. For instance, a dual conductive network textile-based composite (DCNTC) for underwater strain sensing was developed by combining continuous spraying with step-wise curing of Ecoflex elastomer (Fig. 10g) [187]. MXene and fluorinated carbon nanotubes formed synergistic conductive pathways embedded within a flexible polymer matrix. SEM comparisons (Fig. 10h, i) show the transformation from smooth nylon fibers to a roughened conductive surface. This sensor maintained superhydrophobicity after 5000 stretching cycles, sandpaper abrasion, and water impact.

Building upon the simplicity of spray-coating, layer-by-layer (LBL) assembly enables molecular-level precision. A superhydrophobic conductive NS fabric (OTCNS) was created through mussel-inspired LBL assembly using a metal-phenolic network as an adhesive platform for sequentially depositing cellulose nanofiber-dispersed carbon nanotubes onto nylon/spandex fabric (Fig. 10j) [188]. The color transition from white to blue-violet confirmed successful TA-Fe^3+^ complex deposition (Fig. 10k, l), while SEM analyses (Fig. 10m, n) revealed controlled nanoscale roughness. This textile maintained superhydrophobicity under 100% strain and achieved efficient photothermal conversion (84.7 °C at 450 mW cm^−2^).

Beyond deposition-based methods, in situ growth offers unparalleled adhesion through direct nucleation of photothermal nanoparticles from molecular precursors on fiber surfaces, forming chemical bonds that anchor the functional layer. Examples include polyaniline growth on polyester for Joule heating [189] and bio-inspired mineralization for robust multifunctional coatings [190, 191]. The key advantage is creating conformal coatings that preserve fabric breathability.

Further expanding the fabrication toolbox, dip-coating and templating methods provide alternative pathways. Dip-coating ensures uniform coverage on complex woven structures, as demonstrated in self-healing cotton fabrics via sequential deposition of MXene and polyacrylate layers [154]. Sol–gel and salt templating create three-dimensional porous structures enhancing surface roughness and thermal insulation [129, 192, 193].

Collectively, the progression from spray-coating to LBL assembly (Fig. 10g–n) illustrates a fundamental evolution from simple surface coverage to sophisticated interfacial engineering, enabling researchers to select the optimal approach based on requirements for durability, precision, and scalability.

### Strategic Integration: Toward Hybrid Fabrication Paradigms

The distinction between top-down and bottom-up approaches fundamentally shapes the resulting material’s properties and performance characteristics. As visualized in Fig. 10, each paradigm offers complementary advantages: top-down methods create intrinsic durability on compatible substrates, while bottom-up approaches provide unmatched versatility in material combinations. The most advanced fabrication strategies now recognize that these approaches are not mutually exclusive but rather complementary.

A critical issue during scale-up is that coating adhesion measured on small laboratory samples may not be maintained during continuous textile manufacturing. In roll-to-roll or pad-dry-cure processes, uneven liquid uptake, tension-induced fiber deformation, rapid drying, and repeated bending over rollers can generate spatial variations in coating thickness and interfacial bonding strength. Coatings constructed mainly through physical adsorption, van der Waals interactions, or electrostatic interactions are particularly vulnerable to particle shedding during washing and wearing. This limitation has also been noted in scalable MOF-textile fabrication, where conventional deposition, dip-coating, hot-pressing, and spraying methods often suffer from poor washing and wearing durability because of weak non-covalent interactions. In contrast, diazonium chemistry can form aryl–surface covalent bonds with bond energies above 50 kcal mol^−1^, providing a stronger anchoring route for functional coatings on cellulose fibers [194].

Even for chemically anchored or in situ grown systems, however, scale-up still involves measurable performance degradation. For example, scalable ZIF-67-cotton textiles fabricated at a size of 30 m × 0.25 m retained most of the MOF coating after ISO 105 C10 laundering, but SEM still revealed partial MOF particle loss at some locations; meanwhile, the tensile strength decreased from 31.43 to 25.98 MPa and air permeability decreased from 252.7 to 203.5 mm s^−1^ after modification [194]. Similarly, in a CuBTC@cotton system prepared via layer-by-layer in situ growth, 15 laundering cycles caused slight CuBTC particle loss, with WCA decreasing to 156.6° ± 1.8° and WSA increasing to 4.3° ± 1.1°. After 500 mechanical abrasion cycles, the fiber structure was seriously damaged, and WCA decreased from 168.4° ± 1.6° to 152.6° ± 2.4°, while WSA increased from 2.3° ± 0.3° to 16.9° ± 0.8° [195]. These results indicate that industrial scalability should be evaluated not only by sample size or production throughput, but also by adhesion retention after washing, abrasion, bending, and continuous-processing deformation. Therefore, future fabrication studies should report coating mass loss, WCA/SA retention, photothermal retention, tensile strength loss, air permeability change, and spatial uniformity along the processed fabric length.

The future of photothermal superhydrophobic textile fabrication lies in intelligent hybrid strategies that synergize the strengths of both paradigms. For instance, combining plasma pre-treatment (top-down) with subsequent in situ growth (bottom-up) creates systems with optimized interface strength and functional performance. Such integrated approaches represent the next frontier in textile engineering, promising materials with unprecedented durability, functionality, and adaptability for demanding applications.

## Comprehensive Evaluation Index System for Photothermal Superhydrophobic Fabrics

The performance evaluation of photothermal superhydrophobic fabrics requires a multidimensional approach that integrates wettability, photothermal conversion capability, and substrate-specific properties. Establishing a standardized evaluation system is critical for quantifying material efficiency, facilitating comparative analysis, and guiding industrial scalability. This section systematically outlines key performance indicators, their theoretical foundations, measurement methodologies, and relevance to real-world applications.

### Superhydrophobic Performance Indicators

#### WCA and SA

As established in Sect. 2, WCA and SA serve as fundamental metrics for evaluating superhydrophobic properties, with particular significance for textile substrates due to their complex surface topography. While WCA quantifies static liquid repellency, SA provides crucial information about dynamic droplet behavior, which is especially relevant for self-cleaning applications where efficient droplet roll-off is essential. The measurement of these parameters on fabric surfaces requires careful consideration of their fibrous and porous nature, as standardized in ASTM D7334 for contact angle measurement on inclined surfaces and ASTM D724 specifically adapted for textile substrates [196–200]. These protocols specify controlled conditions including droplet volume (typically 5–10 μL), temperature (20 ± 2 °C), and relative humidity (65% ± 5%) to ensure reproducible results across different laboratories.

For textile applications, the interpretation of WCA and SA values must account for fabric structure variations. Woven fabrics with tight weaves generally exhibit more consistent measurements compared to nonwoven or knitted structures, where surface heterogeneity can lead to wider measurement distributions. Furthermore, the directional dependence of these measurements along warp and weft directions in woven fabrics necessitates multidirectional assessment for comprehensive characterization. A high WCA (> 150°) combined with a low SA (< 10°) indicates robust superhydrophobicity, but additional considerations such as droplet penetration resistance and capillary effects in fabric interstices provide supplementary insights for practical applications [181–184]. The durability of these wetting properties under mechanical stress and environmental exposure ultimately determines the real-world viability of photothermal superhydrophobic textiles.

#### Mechanical Stability (Durability)

The mechanical durability of superhydrophobic coatings on textile substrates represents a critical performance indicator that directly determines practical applicability and service life. Taber abrasion testing according to ASTM D4060 provides standardized evaluation by simulating long-term wear through rotating abrasive wheels under controlled pressure conditions [126, 201–203]. This method quantitatively assesses coating durability by measuring WCA retention and mass loss after specified abrasion cycles, with fabrics maintaining WCA > 150° after 1,000 cycles under 250-g load considered mechanically robust for most applications. Complementary assessment methods include Martindale abrasion (ISO 12947) for simulating fabric-to-fabric friction encountered in wearable applications and sandpaper abrasion testing for evaluating extreme wear resistance under harsh conditions.

Additionally, laundering durability evaluated through AATCC 61 or ISO 6330 protocols assesses coating stability against detergent chemicals and mechanical agitation during washing cycles [204–207]. The degradation pattern of WCA after multiple washing cycles provides insights into coating adhesion quality and chemical resistance. For photothermal superhydrophobic fabrics, maintaining functionality after repeated laundering is particularly challenging due to combined chemical and mechanical stresses that can compromise both superhydrophobicity and photothermal activity.

The mechanical stability of these multifunctional textiles is primarily governed by interfacial bonding strength between functional coatings and textile substrates. Advanced strategies such as covalent grafting through plasma pretreatment, self-healing polymer networks, and multilayer composite structures significantly enhance abrasion resistance while preserving photothermal functionality [126, 138, 169, 208]. For instance, fabrics incorporating polydopamine adhesion layers or elastic polymers like PDMS demonstrate superior WCA retention (> 90%) after extensive abrasion, ensuring long-term performance in demanding applications such as protective workwear and outdoor equipment. These durability enhancements, coupled with appropriate testing methodologies, enable the development of photothermal superhydrophobic textiles that balance advanced functionality with practical robustness.

### Photothermal Performance Indicators

#### Light-Induced Temperature Rise

The light-induced temperature rise serves as a fundamental and directly observable metric for evaluating the photothermal efficacy of functional textiles. This parameter is particularly significant for fabric substrates, as their porous, flexible nature introduces unique thermal behaviors distinct from rigid materials. When assessing photothermal superhydrophobic fabrics, temperature elevation profiles are typically captured using infrared thermography or embedded micro-thermocouples under controlled illumination conditions, most commonly with solar simulators calibrated to 1 kW m^−2^ (AM 1.5 G spectrum) or specific wavelength lasers [209–211]. The transient temperature profile, characterized by the heating rate, maximum equilibrium temperature (*T*_max_), and cooling dynamics, provides critical insights into the material’s practical capabilities.

For textile applications, several factors specific to fabric substrates significantly influence temperature performance. The porous structure of fabrics promotes convective heat loss, which often results in lower equilibrium temperatures compared to solid substrates under identical illumination. Consequently, achieving rapid temperature increases (e.g., > 40 °C within 60 s) requires optimized light absorption and minimized thermal mass. Different applications demand distinct temperature profiles: deicing applications typically require moderate temperatures (40–60 °C) sustained over large areas, while antibacterial functionality may need higher temperatures (> 70 °C) localized at contamination sites [119, 139, 212, 213]. The fabric’s weave structure and fiber composition further modulate thermal response, with tighter weaves generally providing better heat retention but potentially compromising flexibility.

Standardized testing protocols must account for these fabric-specific characteristics. Samples should be tested under realistic conditions, including varying airflow velocities (0–2 m s^−1^) to simulate wearable scenarios, and different backing materials (e.g., skin simulants) to replicate actual use environments. The thermal imaging should capture spatial temperature distribution across the textile surface, identifying potential hotspots or inefficient heating zones that might affect comfort or functionality [139, 214–216]. This comprehensive assessment ensures that photothermal textiles meet both performance requirements and practical usability standards for their intended applications.

#### Spectral Absorptance (Solar Absorptivity)

Spectral absorptance (*α*) represents a crucial intrinsic property determining a material’s capability to convert incident light into thermal energy. For photothermal textiles, accurate measurement and optimization of this parameter require special considerations due to the complex topography and multi-scale structures characteristic of fabric substrates. The absorptance is quantitatively defined as *α*(*λ*) = 1 − *R*(*λ*) − *T*(*λ*), where *R*(*λ*) is reflectance and *T*(*λ*) is transmittance at specific wavelengths, typically measured across the solar spectrum (300–2500 nm) using UV–Vis–NIR spectroscopy with integrating spheres [217–219].

Textile substrates present unique challenges for absorptance characterization due to their surface roughness, fiber orientation, and porous architecture. Unlike smooth surfaces, fabrics exhibit significant light scattering, necessitating specialized measurement configurations that account for diffuse reflectance and transmittance [217, 219]. For accurate results, samples should be measured at multiple locations and orientations, with averaging to accommodate structural variations. High-performance photothermal textiles typically achieve broadband absorptance exceeding 90% through strategic material selection and structural engineering. Carbon-based nanomaterials (e.g., graphene, carbon nanotubes) provide excellent broadband absorption, while plasmonic nanoparticles (e.g., Au, Ag) offer tunable resonance peaks in specific wavelength ranges [87, 109, 117, 213, 220].

Microstructural engineering plays a pivotal role in enhancing light absorption on fabric substrates. Hierarchical structures combining micro-scale fiber topography with nano-scale surface features enable multiple light scattering and trapping effects, significantly prolonging the optical path length and increasing absorption probability [112, 119, 212, 217, 221]. Additionally, the textile’s inherent porosity can be leveraged to create light-trapping cavities that further enhance absorption. However, excessive material loading to boost absorption must be balanced against maintaining fabric flexibility, breathability, and comfort. Recent advances include the development of wavelength-selective absorbers that maximize solar absorption while minimizing thermal radiation losses, particularly valuable for outdoor applications where radiative cooling can compromise efficiency [222–224].

#### Photothermal Conversion Efficiency (*η*)

Photothermal conversion efficiency (*η*) is the key figure of merit for photothermal textiles, describing how effectively incident optical energy is converted into useful heat. In practice, *η* is not a single “absolute” number unless the definition and test boundary conditions are clearly specified. Two closely related metrics are therefore recommended: (i) an internal (absorption-normalized) efficiency that evaluates heat generation per absorbed optical power, and (ii) an external (incident-normalized) efficiency that evaluates the net heat delivered to the environment per incident optical power [225, 226]. For textile substrates—where strong scattering, porosity-driven convection, and variable contact to the surroundings are inherent—reporting both metrics (together with the test setup) greatly improves cross-study comparability.

A general transient energy balance for a photothermally heated specimen can be expressed as [226, 227]:

21 $$m_{{{\mathrm{eff}}}} C_{{{\mathrm{eff}}}} \frac{{{\mathrm{d}}T}}{{{\mathrm{d}}t}} = Q_{{{\mathrm{in}}}} - Q_{{{\mathrm{out}}}}$$

Here, *m*_eff_ *C*_eff_ is the effective heat capacity of the measured system (photothermal textile, holder, and any backing layer included in the thermal mass), *Q*_in_ is the light-derived heat generation, and *Q*_out_ is the total heat dissipation to the surroundings (conduction, convection, and thermal radiation). In most photothermal efficiency protocols, these dissipation channels are lumped into an effective heat-transfer term* hS*(*T* − *T*_env_), where h is an effective heat-transfer coefficient and *S* is the effective heat-loss area under the given mounting and airflow conditions [225, 226]. In essence, Eq. (21) treats the textile as a thermal system: its temperature rises when photothermal heat gain exceeds heat loss, and equilibrium is reached when the two balance. Therefore, the measured temperature–time curve reflects not only the coating’s intrinsic photothermal ability but also the thermal mass and heat dissipation environment of the entire test setup.

Under monochromatic irradiation (e.g., a laser at wavelength *λ*), the absorbed optical power is commonly written as *I*·(1 − 10^−*Aλ*^) [225, 226], where I is the incident laser power and *Aλ* is the absorbance at *λ*. The internal photothermal conversion efficiency is then obtained at steady state (d*T*/d*t* = 0) as [227, 228]:

22 $$\eta_{{\mathrm{int}}} = \frac{{hS(T_{\max } - T_{{{\mathrm{env}}}} ) - Q_{{{\mathrm{dis}}}} }}{{I(1 - 10^{ - A\lambda } )}}$$

In essence, *η* quantifies how efficiently absorbed monochromatic light is converted to usable heat, with background absorption subtracted. Its value is meaningful only when heat-loss conditions are measured consistently. In this formulation, *T*_max_ is the steady-state maximum temperature under illumination, *T*_env_ is the ambient (surrounding) temperature, and *Q*_dis_ (or *Q*_0_ in some reports) accounts for baseline heat input arising from light absorption by the blank substrate/holder/solvent, measured independently under identical illumination. This Roper-type framework is widely adopted across photothermal systems, including NIR (e.g., 1064 nm) excitation, where *Aλ* is measured at the laser wavelength in supporting information protocols [225, 226].

For broadband solar (or solar-simulator) illumination, it is often more meaningful for wearable textiles to report an external solar–thermal efficiency referenced to incident power density (*I*_sun_, W m^−2^) and illuminated area (*A*, m^2^) [227–229]:

23 $$\eta_{{{\mathrm{ext}}}} = \frac{{hS(T_{\max } - T_{{{\mathrm{env}}}} ) - Q_{{{\mathrm{dis}}}} }}{{I_{{{\mathrm{sun}}}} A}}$$

This external efficiency reflects the practical solar–thermal output under broadband irradiation, directly relevant to wearable textiles evaluated under sunlight or solar-simulator conditions. If the goal is to isolate the “material-intrinsic” conversion capability from optical collection effects, the absorbed fraction should be included explicitly. For scattering textiles, a solar-weighted absorptance ($\overline{\alpha}_{{{\mathrm{solar}}}}$) is preferably obtained from integrating-sphere reflectance/transmittance spectra as [218, 230]:

24 $$\overline{\alpha}_{{{\mathrm{solar}}}} = \frac{{\int_{{\lambda_{1} }}^{{\lambda_{2} }} {\left[ {1 - R\left( \lambda \right) - T\left( \lambda \right)} \right]E_{{{\mathrm{sun}}}} \left( \lambda \right){\mathrm{d}}\lambda } }}{{\int_{{\lambda_{1} }}^{{\lambda_{2} }} {E_{{{\mathrm{sun}}}} \left( \lambda \right){\mathrm{d}}\lambda } }}$$

This equation converts wavelength-dependent absorption into a single solar-weighted value by accounting for the solar spectrum's intensity distribution. In essence, it answers: how much of real sunlight—rather than monochromatic light—the textile can absorb. The absorption-normalized efficiency can be written as *η*_int, solar_ = *η*_ext_/$\overline{\alpha }$_solar_. This distinction is important because many high-performing photothermal fabrics achieve excellent external efficiency primarily by maximizing broadband absorption while also managing heat losses through structure and mounting.

For porous textile substrates, the heat-transfer coefficient should not be treated as a universal constant because intrinsic porosity and air permeability can introduce additional convective heat loss through inter-fiber voids and through-thickness air exchange. Therefore, when applying cooling curve-based photothermal efficiency equations, *h*S should be regarded as an effective or apparent heat-loss term, h_eff_ S_eff_, rather than a purely material-independent convective coefficient.

Practically, *h*_eff_ *S*_eff_ should be experimentally determined for each textile specimen under the same mounting configuration, airflow condition, backing layer, exposed area, ambient temperature, and humidity using the cooling curve after the light source is switched off [204–207]:

25 $$\theta = T - T_{{{\mathrm{env}}}} /T_{\max } - T_{{{\mathrm{env}}}}$$

26 $$\ln \left( \theta \right) = - t/\tau_{{\mathrm{s}}}$$

27 $$h_{{{\mathrm{eff}}}} S_{{{\mathrm{eff}}}} = m_{{{\mathrm{eff}}}} C_{{{\mathrm{eff}}}} /\tau_{{\mathrm{S}}}$$

In this way, porosity-induced convection is incorporated into the measured effective heat-loss coefficient. For cross-study comparison, fabric porosity, thickness, air permeability, projected/exposed area, clamping method, backing or insulation condition, ambient airflow, humidity, and environmental temperature should be reported together with *η*. If samples with different porosities are compared, *h*_eff_ can be normalized using the same projected heat-loss area and measured under identical airflow conditions, rather than adopting a fixed *h* value from dense films, solution systems, or rigid substrates.

It should be noted that a universal correction formula that relates *h* only to fabric porosity, such as *h* = *f*(*ε*), is not generally applicable, because convective heat loss in textiles is also affected by pore-size distribution, fabric thickness, weave or knit architecture, surface roughness, sample orientation, and airflow velocity. Therefore, sample-specific cooling curve determination under standardized boundary conditions is currently a more reliable route for comparing photothermal conversion efficiency among porous textile systems.

Measurement standardization is essential for meaningful efficiency comparisons across different photothermal textiles. While ASTM E1980 provides guidance for solar absorptance evaluation, robust *η* assessment should additionally control airflow, humidity, and background temperature and should specify sample clamping/mounting in a way that reflects real use scenarios [230]. For fabric substrates, it is often informative to test both free-standing configurations and backed configurations (e.g., insulation layers or skin simulants), because the same photothermal coating can yield substantially different *η*_ext_ depending on heat-loss pathways.

Advanced photothermal textiles incorporating carbon nanomaterials, MXene/2D absorbers, or optimized plasmonic architectures have reported very high conversion efficiencies when evaluated under well-controlled conditions [21, 25, 30, 33, 39]; however, the reported value depends on whether the metric is internal vs external, monochromatic vs broadband illumination, and how *Q*_dis_ and *hS* are determined. For wearable applications, high practical efficiency must be achieved without sacrificing drape, breathability, softness, and safety. Strategies such as localized coating deposition, gradient distributions, and multilayer (absorber/insulator/emitter) designs can improve *η*_ext_ by simultaneously increasing ᾱ_solar_ and suppressing convective/conductive losses [2, 48]. Finally, because textiles experience repeated bending, abrasion, and laundering, reporting η retention after mechanical deformation and washing cycles is crucial to demonstrate that laboratory-measured performance translates to real-world utility.

### Fabric Substrate Performance Indicators

#### Mechanical Integrity Under Multifunctional Stress

The mechanical integrity of fabric substrates under combined stress conditions is paramount for ensuring the practical viability and longevity of photothermal superhydrophobic textiles. This evaluation must encompass tensile, tear, and bending properties, which collectively determine how the material withstands mechanical challenges in real-world applications. Tensile strength testing according to ASTM D5035 provides critical data on the maximum load-bearing capacity of fabrics, with performance retention exceeding 85% after functionalization indicating minimal structural compromise [184, 231, 232]. Concurrently, tear resistance evaluation following ASTM D5587 assesses the fabric’s ability to resist crack propagation, particularly crucial for protective gear and technical textiles where accidental damage may occur. The preservation of tear strength after coating application often depends on uniform dispersion of photothermal nanomaterials and strong interfacial adhesion to prevent stress concentration.

Flexural rigidity measurement via ASTM D1388 (cantilever test) quantitatively evaluates fabric stiffness, a key determinant of wearability and comfort. Coatings causing more than 15% increase in bending length typically result in unacceptable hand feel, necessitating optimization through flexible polymer matrices or patterned deposition techniques. Advanced characterization methods including scanning electron microscopy of stressed regions and cyclic fatigue testing (over 10,000 cycles at 10%–20% strain) provide insights into coating-substrate interfacial durability under repetitive deformation [33, 187]. These comprehensive mechanical assessments ensure that photothermal superhydrophobic textiles maintain structural integrity while delivering advanced functionality, enabling applications from smart apparel to industrial textiles where mechanical robustness is essential for long-term performance.

#### Thermo-Physiological Comfort

Thermo-physiological comfort represents a critical performance dimension that directly influences wearer acceptance and practical utility of photothermal superhydrophobic textiles. This multifaceted attribute encompasses air permeability, moisture management, and thermal properties that collectively govern human thermoregulation and subjective comfort. Air permeability assessment following ASTM D737 quantifies the ease of air passage through fabric, with maintained permeability above 100 cm^3^/cm^2^/s being essential for preventing heat and moisture accumulation in wearable applications [139, 233]. Photothermal superhydrophobic coatings must be engineered with porous architectures or selective deposition patterns to preserve adequate airflow while maintaining functional performance.

Moisture vapor transmission rate (MVTR) evaluation according to ASTM E96 measures water vapor permeability, a critical factor in maintaining dry comfort during physical activity. Optimal functionalization should limit MVTR reduction to less than 20% compared to untreated fabrics, achieved through nanoscale coating designs that allow vapor diffusion while blocking liquid penetration [233]. Complementary liquid moisture management testing (AATCC 195) provides additional insights into dynamic wetting behavior and directional transport capabilities. Thermal conductivity assessment via ASTM D7984 combined with infrared thermography analysis enables comprehensive characterization of heat transfer properties and surface temperature distribution, identifying potential hot spots that may cause discomfort during extended wear.

The integration of comfort-oriented design strategies, such as breathable coating patterns, moisture-wicking finishes, and flexible nanocomposites, ensures optimal balance between advanced functionality and wearability. Subjective evaluation through standardized sensory testing (e.g., ASTM D6667) further validates comfort performance, with maintained fabric softness (bending length < 3 cm) and minimal stiffness increase being critical for user acceptance. These comprehensive comfort assessments guarantee that photothermal superhydrophobic textiles meet practical requirements for everyday wear while delivering enhanced functionality.

#### Environmental and Chemical Resilience

Environmental and chemical resilience constitutes a vital performance category that determines the long-term stability and application scope of photothermal superhydrophobic textiles under various exposure conditions. UV stability assessment following AATCC 16 evaluates photocatalytic degradation resistance, with high-performance fabrics maintaining over 80% of original superhydrophobicity after 500 h of accelerated weathering [119, 184, 231]. The incorporation of UV-stabilizing additives or inherently stable photothermal materials significantly enhances resistance to solar degradation, ensuring durability in outdoor applications.

Chemical resistance testing encompasses comprehensive exposure to acids, alkalis, oils, and solvents according to standards such as AATCC 130 for stain resistance and ISO 105-X12 for rubbing fastness. Textiles demonstrating stable performance across broad pH ranges (typically 3–11) enable applications in harsh environments including chemical processing, marine operations, and medical sterilization [139, 234]. Quantitative evaluation involves measuring WCA retention, photothermal efficiency, and mechanical properties after chemical exposure, providing crucial data for material selection based on specific application requirements.

Thermal stability assessment through thermogravimetric analysis (TGA) and practical thermal cycling tests (− 20 to 100 °C) determines performance maintenance under temperature extremes encountered during processing and use. Additionally, comprehensive weathering resistance evaluation following ASTM G155 provides accelerated aging data that predicts long-term outdoor performance by simulating combined environmental factors including UV radiation, moisture, and temperature variations. The development of self-healing coatings and robust nanostructures further enhances environmental resilience, enabling extended service life in demanding applications from architectural textiles to protective equipment, where sustained performance under challenging conditions is essential for practical utility.

Following the assessment of environmental and chemical resilience, it becomes evident that a holistic and standardized evaluation framework is indispensable for advancing photothermal superhydrophobic textiles from laboratory prototypes to commercially viable products. To provide such a framework, Table 2 summarizes representative photothermal superhydrophobic fabric systems according to the key indicators discussed in Sects. 7.1–7.3, including superhydrophobic performance, mechanical durability, photothermal response, solar absorptivity, photothermal conversion efficiency, substrate mechanical integrity, physiological comfort, and environmental resilience.

**Table 2:** Comprehensive performance evaluation indices for photothermal superhydrophobic fabrics (under 1.0 sun irradiation unless otherwise noted)

Fabric system (reference)	WCA and SA	Mechanical Durability	*T*_max_ (°C)	Solar absorptivity	*Η*	Physiological comfort	Refs.
PDA–MTMS @cotton	WCA ≈ 163° SA ≈ 6°	No WCA loss after abrasion and tape tests	93	High	NA	NA	[119]
TiN/HPDMS/TEOS@cotton	WCA = 162°	Self-heals after scratch; durable through washing cycles	89.95	NA	~ 91%	Air permeability decreased by only ~ 5.9%, with good softness retained	[159]
CNC–MXene/PA @cotton	WCA = 151°	Washable (> 99.9% WCA retention after 10 laundry cycles)	83.1	96%	NA	NA	[154]
PDMS/PP y@cotton	WCA = 160°	Exceptional durability (no WCA drop after repeated icing/deicing cycles)	107.2	> 95%	NA	NA	[241]
PDA/PEI/GA/Ag/PDMS @cotton	WCA = 159.6°	Maintains SH under high temperatures, corrosion, washing and abrasion	70.4	NA	NA	NA	[242]
PDMS/CuS /PTA@Cotton	WCA ≈ 153°	Durable through abrasion and washing cycles	80.2	High	NA	Breathability was tested, but no value was reported	[184]
Ag/PDMS @cotton	WCA = 171.3° SA = 56.4°	Survives numerous wash cycles and abrasions with minimal WCA drop	46.1	NA	NA	Low PDMS loading caused only slight stiffness increase, while excess PDMS led to a rubber-like hand feel	[93]
MXene/HDTMS/SiO₂@cotton	WCA ≈ 152° SA ≈ 5.2°	Maintains SH under high temperatures, underwater ultrasonic, washing and abrasion	45.1	NA	NA	NA	[139]
CS/ACNT/ODA@cotton	WCA = 160.3°	Good durability (no WCA drop after abrasion; laundry-stable)	70	NA	NA	Fabric mass increased from 1.283 to 1.398 g, while air permeability decreased by only 13.21 mm/s	[243]
Lignin–TA–MTMS@cotton	WCA = 164.3° SA = 8.5°	Durable after abrasion, tape, washing	54.2	NA	NA	No direct comfort data; fabric thickness increased after modification	[14]
FAS/AgMXene@PLA	WCA = 151.5°	Durable after acid–base corrosion	71.2	NA	87.4%	NA	[244]
PDMS/Fe_3_O_4_/PPy@PET	WCA = 152°	Maintains SH after abrasion, multiple icing/deicing cycles and UV irradiation	74.3	> 90%	NA	NA	[25]
ZIF-8/PP@ cotton	WCA = 167° SA = 2°	Durable after abrasion, washing, high and low temperatures, and exposure to acids and alkalis	80	90%–95%	NA	Breathability was mentioned, but no quantitative data were reported	[33]
PDMS/PPy @Silk	WCA = 155.5° SA = 4.2°	Maintains SH after abrasion, tape-peeling, UV irradiation tests	63.6	NA	NA	NA	[171]
Mx@SPF (asymmetric Janus PET)	WCA = 160°	Durable after water flow impact, finger pressing, sandpaper abrasion and acid–base corrosion	65	NA	NA	Breathability was mentioned, but no quantitative data were reported	[245]

It should be noted that the data summarized in Table 2 are not intended for a simple direct ranking, because different studies adopted different testing conditions, such as irradiation intensity, droplet volume, sample size, abrasion/washing protocols, coating thickness, and environmental conditions. Instead, the table serves as a structured basis for identifying representative performance ranges, best-in-class values, and current reporting gaps. In particular, many studies still lack quantitative solar absorptivity or photothermal conversion efficiency (η), which limits rigorous cross-comparison of photothermal performance [235–238]. Based on this table, the following section further analyzes the reported results from the three dimensions of superhydrophobic performance, photothermal performance, and substrate-related performance.

### Quantitative Benchmarking of Photothermal Superhydrophobic Textiles

To provide a quantitative evaluation beyond the literature summary in Table 2, representative photothermal superhydrophobic textiles were compared across three performance dimensions: superhydrophobicity, photothermal capability, and substrate-related performance. Given variations in droplet volume, irradiation intensity, sample size, and testing protocols, direct ranking is inappropriate; instead, performance ranges and best-in-class values are extracted.

For superhydrophobic performance, most systems achieve WCA > 150°, with values in Table 2 ranging from 151° to 171.3°. Ag/PDMS@cotton shows the highest WCA of 171.3° but a high SA of 56.4° [93]. In contrast, ZIF-8/PP@cotton combines WCA = 167° with SA = 2°, representing a more balanced low-adhesion state [33]. PDA–MTMS@cotton (163°/6°), Lignin–TA–MTMS@cotton (164.3°/8.5°), and PDMS/PPy@Silk (155.5°/4.2°) also show favorable combinations [14, 119, 171]. These comparisons indicate that WCA alone is insufficient; low SA and post-durability retention are equally important. Systems maintaining WCA > 150° and SA < 10° after mechanical or chemical damage are more practically meaningful [14, 33, 119, 171]. Regarding durability, PDA–MTMS@cotton retains its WCA after abrasion [119], CNC–MXene/PA@cotton retains > 99.9% after 10 laundering cycles [154], and MXene/HDTMS/SiO_2_@cotton remains superhydrophobic after high-temperature treatment and abrasion [139]. PDMS/Fe_3_O_4_/PPy@PET and PDMS/PPy@Silk also retain superhydrophobicity after abrasion and UV exposure [25, 171], highlighting the importance of elastic polymer protection.

For photothermal performance, *T*_max_ under 1.0 sun ranges from 45.1 to 107.2 °C. PDMS/PPy@cotton reaches 107.2 °C with absorptivity > 95% [122]. PDA–MTMS@cotton and TiN/HPDMS/TEOS@cotton reach 93 °C and 89.95 °C, respectively [119, 159]. Biomass-derived Lignin–TA–MTMS@cotton reaches 54.2 °C [14], while MXene/HDTMS/SiO_2_@cotton reaches 45.1 °C [139], offering milder output but better comfort or sustainability. Among reported efficiencies, TiN/HPDMS/TEOS@cotton achieves ~ 91% and FAS/AgMXene@PLA reaches 87.4% [159, 244]. However, many systems report only *T*_max_ rather than standardized η, limiting cross-comparison. For spectral absorptance, CNC–MXene/PA@cotton reports 96% [154], PDMS/PPy@cotton > 95% [122], PDMS/Fe_3_O_4_/PPy@PET > 90% [25], and ZIF-8/PP@cotton 90–95% [33], confirming MXene and PPy as effective absorbers. Nonetheless, high absorptance may reduce breathability or flexibility, so photothermal evaluation should combine *T*_max_, heating rate, *η*, cyclic stability, and retention after washing or abrasion.

Regarding substrates, cotton remains dominant due to low cost, breathability, and ease of modification [14, 33, 119, 122, 139, 154, 184, 242, 243]. However, diversification toward PLA, PET, silk, and Janus PET is evident [25, 171, 244, 245]. FAS/AgMXene@PLA offers biodegradability with WCA = 151.5°, *T*_max_ = 71.2 °C, and *η* = 87.4% [244]. PDMS/Fe_3_O_4_/PPy@PET combines WCA = 152°, *T*_max_ = 74.3 °C, and absorptivity > 90% with good stability [25]. PDMS/PPy@Silk provides a softer platform with WCA = 155.5°, SA = 4.2°, and *T*_max_ = 63.6 °C [171]. These examples confirm substrate selection as a performance-determining factor.

Overall, current systems have achieved WCA > 160°, SA as low as 2°, *T*_max_ > 100 °C, absorptivity > 95%, and *η* around 90% [33, 93, 122, 159, 171]. However, these best-in-class values are from different systems under non-identical conditions. The key challenge is balancing superhydrophobicity, adhesion, durability, photothermal efficiency, breathability, comfort, and sustainability.

## Versatile Applications of Photothermal Superhydrophobic Textile

**Fig. 11:**
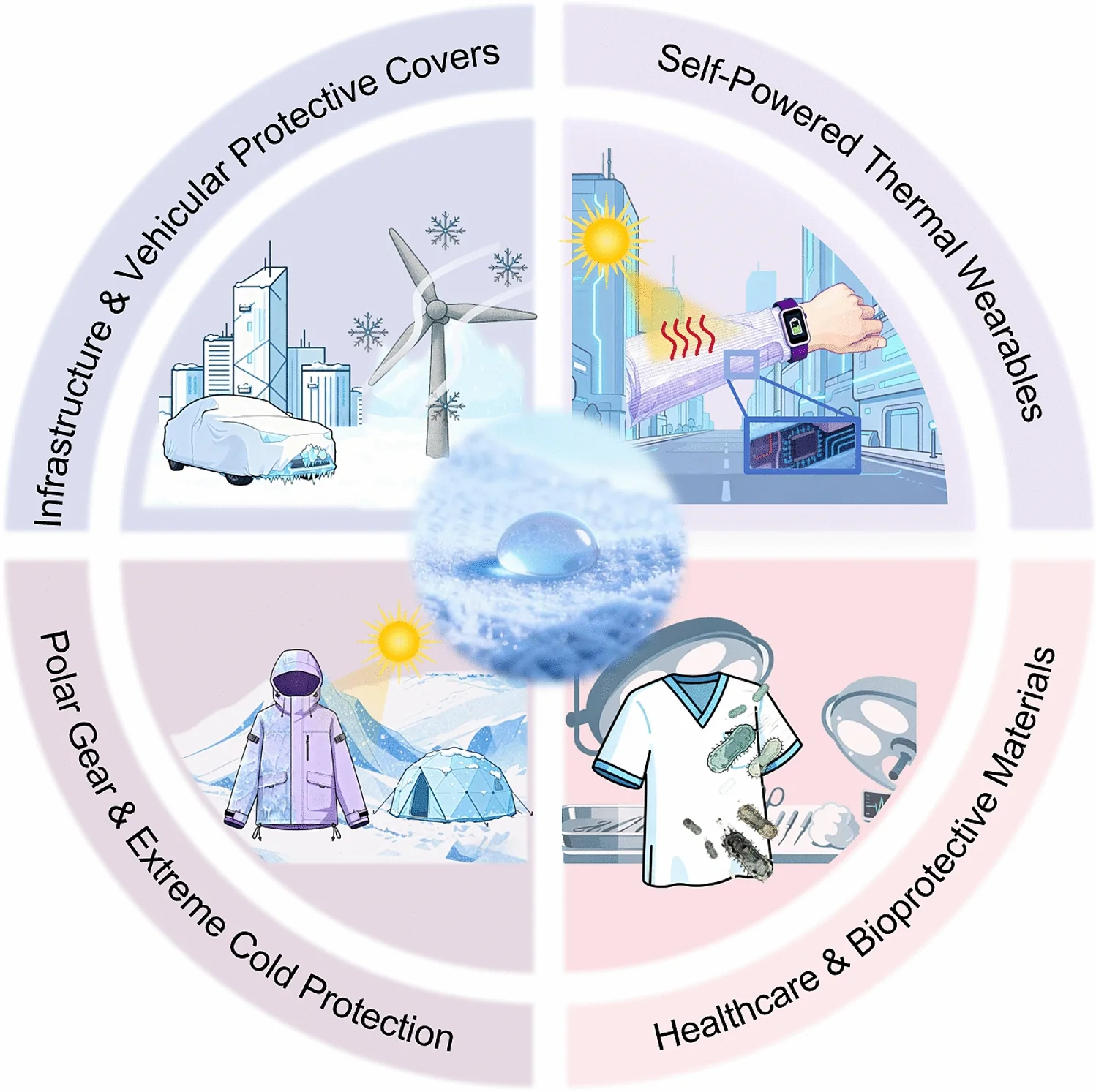
Application scenarios of photothermal superhydrophobic textile materials

The convergence of superhydrophobicity and photothermal functionality in textiles has unlocked a new paradigm of smart materials capable of actively interacting with their environment. These fabrics transcend their traditional passive roles, evolving into multi-functional systems that provide protection, comfort, and even therapeutic benefits [25, 132, 138, 139, 172, 184, 246, 247]. The ability to repel water and simultaneously generate heat upon light irradiation addresses critical challenges across a remarkably diverse range of fields, from safeguarding large-scale infrastructure to enhancing personal healthcare [129, 130, 136, 248–254]. As illustrated in Fig. 11, the application landscape for photothermal superhydrophobic textiles is vast, encompassing scenarios where reliability under harsh conditions, energy efficiency, and biological safety are paramount. This section delves into the specific applications of these advanced materials, exploring the underlying mechanisms and demonstrated benefits in each domain.

### Infrastructure and Vehicular Protective Covers

The operational integrity and longevity of critical infrastructure and vehicles are perpetually challenged by environmental adversities, particularly ice accumulation, moisture ingress, and extreme temperature fluctuations. Traditional protective materials often function passively, whereas photothermal superhydrophobic textiles introduce an active, responsive dimension to protection, leveraging environmental energy for self-maintenance [134, 255–263]. These advanced fabrics are engineered to not only repel water and resist ice formation but also to generate heat upon demand, offering a sustainable and efficient solution for large-scale applications [127, 135, 264–267]. As depicted in Fig. 11, their use cases span from wind turbine blades to architectural canopies and automotive covers, where reliability and minimal maintenance are critical. Recent research has yielded sophisticated multi-modal systems that significantly enhance this protective capability, moving beyond single-functionality toward all-weather operational readiness.

**Fig. 12:**
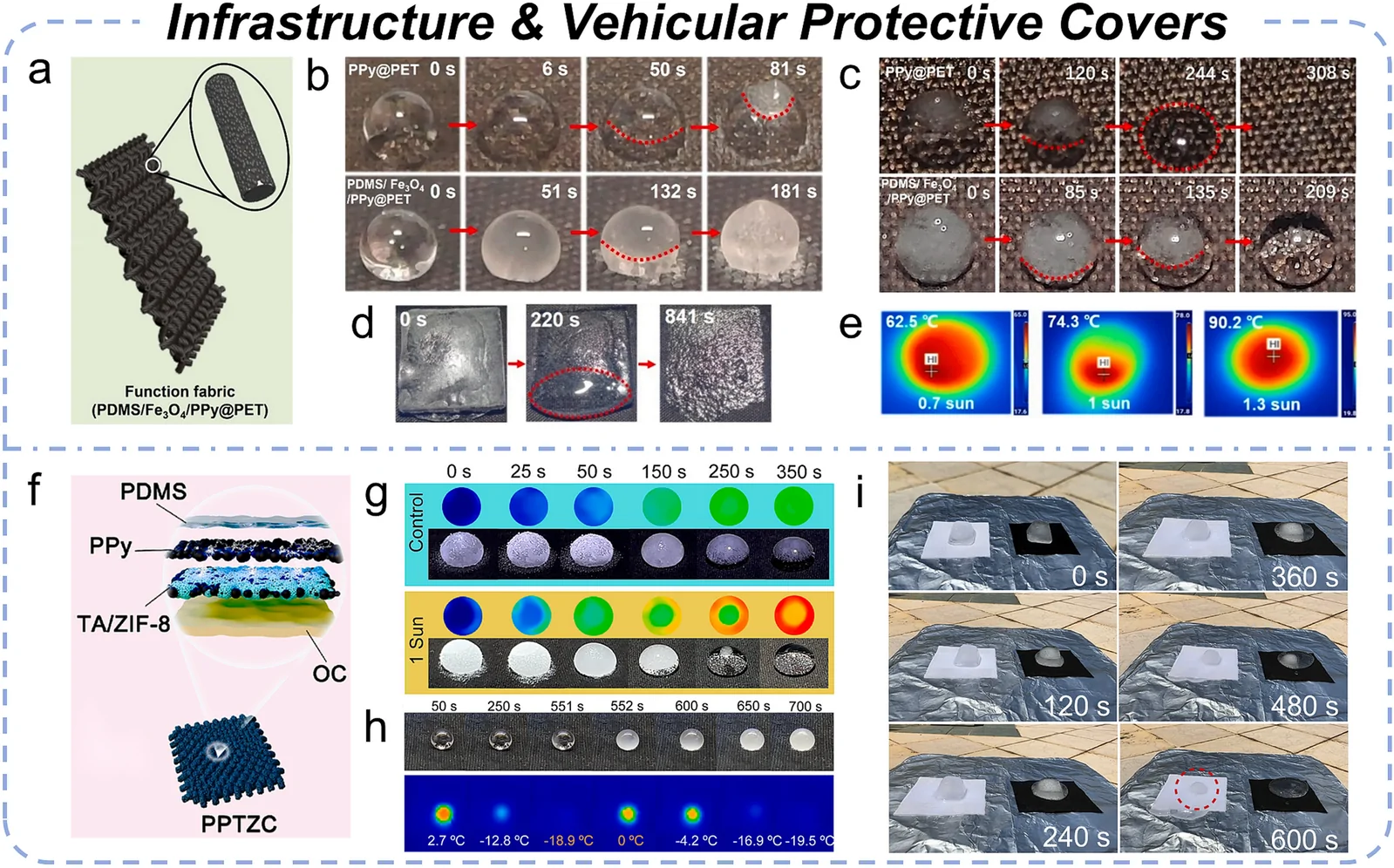
Representative research examples of infrastructure and vehicular protective Covers. **a** Schematic diagram of functional fabric PDMS/Fe_3_O_4_/PPy@PET [25]. **b** Freezing process and time of a droplet (10 μL) on the surface of PPy@PET and PDMS/Fe_3_O_4_/PPy@PET [25]. **c** Photothermal deicing process images on the surface of PPy@PET and PDMS/Fe_3_O_4_/PPy@PET under 1.0 sun irradiation [25]. **d** Melting process and time of ice layer on the PDMS/Fe_3_O_4_/PPy@PET surface [25]. **e** Equilibrium temperature image of PDMS/Fe_3_O_4_/PPy@PET under different light intensities. Reproduced with permission [25]. Copyright 2024, Elsevier. **f** Schematic diagram of PPTZC [33]. **g** Melting process and temperature changes of ice on PPTZC under different irradiation conditions [33]. **h** Actual and infrared images of the process of water droplet freezing on PPTZC [33]. **i** Melting process of ice cubes on the surface of OC and PPTZC in outdoor environments. Reproduced with permission [33]. Copyright 2025, Elsevier

A notable advancement in this domain is the development of a multi-responsive fabric, PDMS/Fe_3_O_4_/PPy@PET, which exemplifies the evolution from simple photothermal systems to integrated, all-weather solutions [25]. As schematically illustrated in Fig. 12a, this functional textile is engineered through a sequential process of polymerizing photothermal/electrothermal PPy onto polyester, incorporating Fe_3_O_4_ nanoparticles to enhance roughness and enable magnetic induction heating, and finally coating with PDMS to achieve superhydrophobicity. This clever design results in a single fabric capable of triple energy conversion: photothermal, electrothermal, and electromagnetic induction thermal. The superhydrophobic character is paramount, providing the first line of defense by significantly delaying ice formation. Figure 12b vividly demonstrates this, showing that a water droplet freezes in just 81 s on a merely hydrophobic surface (PPy@PET) but is delayed for 181 s on the superhydrophobic PDMS/Fe_3_O_4_/PPy@PET fabric at − 10 °C, due to the stable air layer trapped in its micro-/nano-roughness.

The active deicing capabilities are where this fabric truly excels. Its photothermal performance is robust, with the surface equilibrium temperature rising proportionally with light intensity, reaching 74.3 °C under 1 sun irradiation (Fig. 12e). This efficient solar energy harvesting enables practical deicing, as shown in Fig. 12c, d, where ice particles and a continuous ice layer are melted within hundreds of seconds, with meltwater easily shedding from the surface. For scenarios lacking sunlight or requiring rapid response, the fabric’s alternative heating modes are indispensable. Notably, the electromagnetic induction thermal mode demonstrated superior performance, melting an ice layer in merely 444 s, significantly faster than the electrothermal mode (1436 s) [25]. This highlights electromagnetic induction as a highly efficient, scalable, and rapid heating solution for large-area applications like deicing aircraft wings on a tarmac or bridge cables, where speed and energy efficiency are critical.

Further expanding the application paradigm, the PPTZC (polypyrrole/TA/ZIF-8/cotton) textile introduces a hierarchical structure designed for enhanced wearability and sensitivity, integrating sensing capabilities with anti-/de-icing functions [33]. Figure 12f outlines its fabrication, where a hollow core–shell structure derived from ZIF-8 etching provides flexibility and improves the conductive network for sensing. This material exhibits exceptional passive anti-icing performance, delaying ice formation for an impressive 1291.4 s at − 10 °C (Fig. 12h). The infrared images capture the phase-change process of a water droplet, underscoring the effectiveness of the superhydrophobic surface in maintaining a supercooled state. Its active photothermal deicing capability is equally remarkable. Figure 12g shows that under 1.0 sun irradiation, an ice block is almost completely melted within 250 s, far surpassing the minimal melting observed without light. Most importantly, Fig. 12i provides compelling validation in a real-world outdoor winter test. The black PPTZC fabric rapidly melts an ice cube within 600 s, with meltwater sliding off its surface, while an ordinary white cloth retains the ice for a much longer period. This demonstration under natural solar irradiation confirms the practical viability of such textiles for protecting solar panels, communication equipment, or temporary shelters in cold environments.

Overall, the integration of robust superhydrophobicity with versatile, multi-modal heating mechanisms and even embedded sensing, as evidenced by the systems in Fig. 12, marks a significant leap forward for protective covers. These textiles transition from being simple barriers to active, intelligent systems that ensure the reliability, safety, and efficiency of infrastructure and vehicles under the most challenging environmental conditions, paving the way for smarter, more resilient cities and transportation networks.

### Polar Gear and Extreme Cold Protection

Whereas Sect. 8.1 focuses on infrastructure-scale protective covers where durability, coverage area, and long-term outdoor reliability dominate, this section shifts to human-centered cold protection. For polar gear and extreme environments, photothermal superhydrophobic textiles must not only delay icing and remove frost, but also reduce body heat loss, maintain flexibility, preserve comfort, and operate under limited sunlight, strong wind, snow contamination, and prolonged low-temperature exposure. Therefore, the following discussion concentrates on polar clothing, tents, and field equipment, where anti-/de-icing is coupled with thermal insulation, lightweight design, and all-day survival reliability.

Operating in polar regions and other extreme cold environments presents a formidable challenge to both human survival and equipment functionality. Conventional textiles often fail under such conditions, as ice accumulation can compromise insulation, increase weight, and lead to catastrophic failure. Photothermal superhydrophobic textiles have emerged as a transformative solution, offering a multi-faceted defense strategy that combines passive resistance to ice formation with active, on-demand heating capabilities [184, 267–270]. These advanced materials are engineered not merely to insulate but to actively manage the thermal environment, making them indispensable for the next generation of polar exploration gear, protective clothing, and shelters [137, 140, 271–275]. As visualized in Fig. 11, their applications range from expedition tents to high-performance outerwear, where maintaining flexibility, warmth, and dryness is a matter of safety and operational success. Recent innovations have focused on integrating multiple thermal management modes and enhancing durability to withstand the planet’s harshest conditions.

**Fig. 13:**
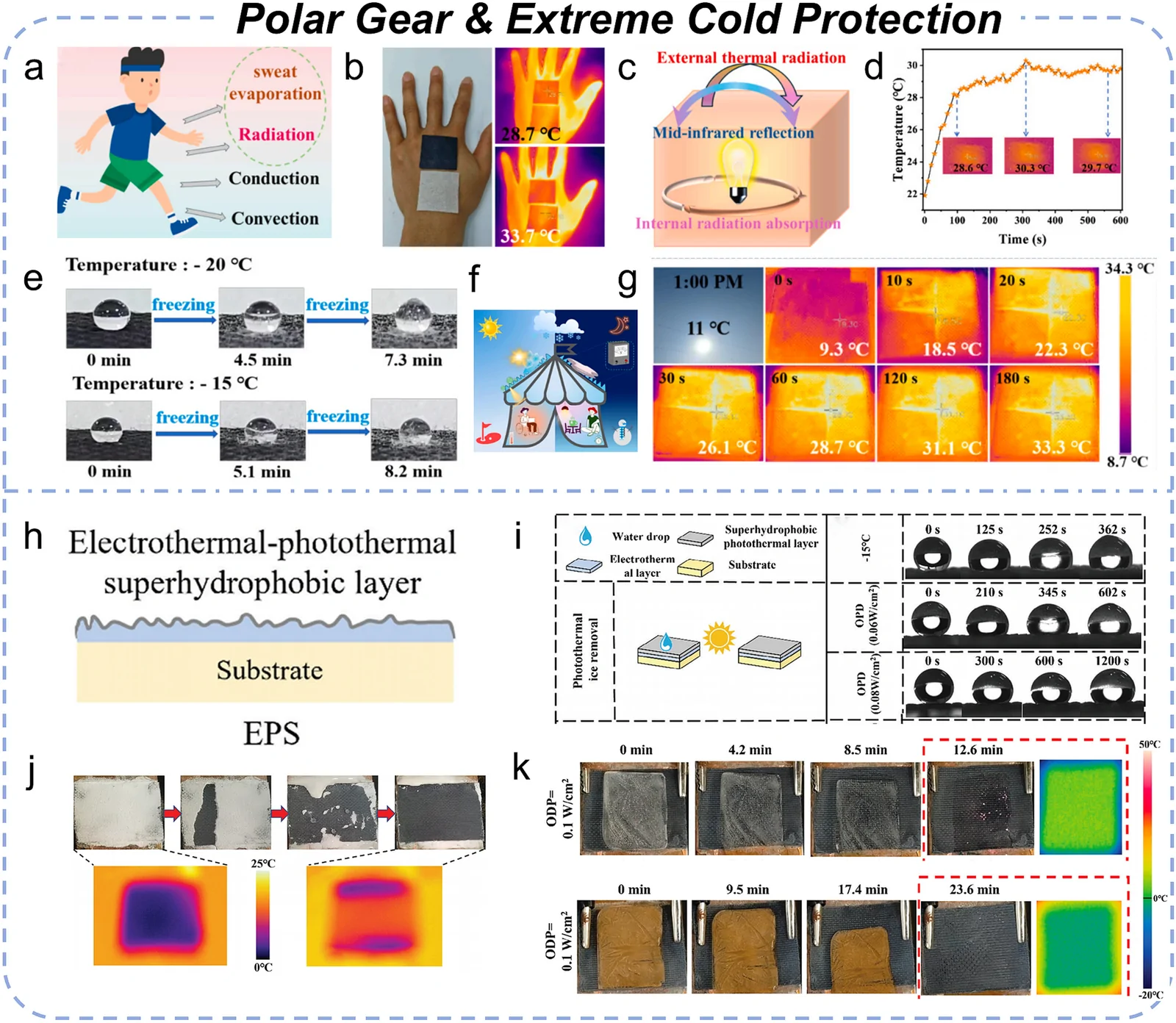
Representative research examples of polar gear and extreme cold protection. **a** The way the human body dissipates heat [244]. **b** Infrared images of different fabrics covering the back of the hand [244]. **c** Simulate the internal heating mechanism of the tent [244]. **d** FAMP simulates the internal heating capacity of tent [244]. **e** Photographs of the freezing process at different temperatures [244]. **f** Schematic diagram of polar tent. **g** Infrared images taken by FAMP in winter sunlight. Reproduced with permission [244]. Copyright 2025, Elsevier. **h** Schematic diagram of EPS coating [167]. **i** Icing process of water droplets on EPS surfaces under different solar heating powers [167]. **j** Schematic of the self-cleaning process; infrared camera images of the EPS coating under 0.1 W cm^−2^ OPD before and after self-cleaning [167]. **k** Process of ice and muddy ice melt by photothermal. Reproduced with permission [167]. Copyright 2024, Wiley

A groundbreaking example is the FAMP textile (comprising PLA nonwoven fabric modified with Ag NPs, MXene, and FAS), designed as a versatile material for polar tents and apparel [244]. This system holistically addresses thermal management by leveraging the human body’s heat dissipation mechanisms, which include radiation, conduction, convection, and evaporation, as depicted in Fig. 13a. A key feature of the FAMP textile is its excellent radiative insulation. Figure 13b demonstrates this effectively; when placed over a hand, the FAMP fabric exhibits a surface temperature of 28.7 °C, significantly lower than the 33.7 °C of the original PLA fabric. This indicates a high infrared reflectivity, effectively trapping body heat and preventing its loss to the environment, a critical factor in reducing the metabolic energy required to stay warm.

The fabric’s utility extends to shelter systems. Figure 13c, d simulates its application in a tent. The fabric’s ability to absorb internal heat radiation (e.g., from a person or a light source) while reflecting mid-infrared rays results in a substantial internal temperature increase of about 8 °C, demonstrating excellent thermal retention capabilities vital for survival shelters in polar nights. Furthermore, the FAMP textile exhibits outstanding passive anti-icing properties. Figure 13e shows that it can delay the freezing of a water droplet for an impressive 90.1 min at − 5 °C and still for 7.3 min at an extreme − 20 °C. This is complemented by robust active deicing: under natural winter sunlight (Fig. 13g), the dry fabric’s temperature rose from 11.0 to 33.3 °C in 180 s, while a wet fabric achieved self-drying through photothermal heating. This dual-mode operation—photothermal deicing during the day and electrothermal deicing at night, as conceptualized in the polar tent design of Fig. 13f—ensures continuous protection.

Complementing the textile-integrated approach, the electro-photothermal superhydrophobic (EPS) nanocomposite coating, illustrated in Fig. 13h, offers a durable surface solution for equipment and rigid shelters [167]. The design philosophy of the EPS coating emphasizes resilience and all-weather operability. Its effectiveness is first evident in its superior anti-icing performance. Figure 13i provides a detailed analysis of water droplet freezing on the EPS surface under different electro-photothermal power densities (EPD/OPD) at − 15 °C. Without active heating, a droplet freezes completely in 362 s. However, when a combined EPD and OPD of 0.06 W cm^−2^ is applied, the surface temperature rises to 16 °C, preventing freezing entirely. This demonstrates a key energy-saving strategy: using solar energy as the primary heat source and supplementing with minimal electrical power only when necessary, a crucial feature for remote polar operations where energy conservation is paramount.

The EPS coating’s practical utility is further underscored by its self-cleaning capability, a critical feature in muddy or contaminated polar environments. Figure 13j illustrates this process vividly. When the coating surface is contaminated with opaque Al_2_O_3_ nanoparticles (simulating dust and grit), its photothermal efficiency is compromised, with the surface temperature reaching only 8 °C under 0.1 W cm^−2^ OPD. However, when water droplets are introduced, they roll off the superhydrophobic surface, carrying away the contaminants. After this self-cleaning action, the surface temperature uniformly rises to 18 °C under the same light intensity, fully restoring its photothermal conversion efficiency. This autonomous maintenance capability ensures long-term operational reliability without manual intervention.

For active deicing, the EPS coating demonstrates remarkable synergy between photothermal and electrothermal mechanisms. Figure 13k captures the deicing process of a 2.5 g ice layer at an ambient temperature of − 20 °C. Using only sunlight (0.1 W cm^−2^ OPD), deicing takes 12.6 min. Using only electricity (0.08 W cm^−2^ EPD) reduces this to 10.6 min. However, when both energy sources are combined, the ice layer melts and slides off in just 2.4 min due to a synergistic temperature rise to 34 °C. More importantly, Fig. 13k also shows the coating’s effectiveness against a practical challenge: soiled ice. Opaque, contaminated ice severely limits photothermal efficiency, taking 17.6 min to melt with sunlight alone. Electrothermal deicing remains effective (15.1 min), but the combined approach again proves superior, clearing the soiled ice in 3.8 min. The meltwater simultaneously cleanses the surface, showcasing a robust, all-weather deicing and self-maintenance capability.

In summary, the advancements represented by the FAMP textile and EPS coating, as detailed in Fig. 13, highlight a strategic evolution in polar gear. The focus has shifted from simple insulation to intelligent, multi-modal systems that provide passive radiative insulation, superior anti-icing, and adaptable active heating. The EPS coating, in particular, with its detailed performance metrics under synergistic heating and self-cleaning actions, demonstrates a critical pathway toward ensuring that explorers, scientists, and their equipment can operate safely and effectively in the most demanding cold environments on Earth, marking a significant leap in our ability to endure and explore the polar frontiers.

### Self-Powered Thermal Wearables

The rapid advancement of wearable technology has created an urgent demand for energy-autonomous systems that can sustainably power electronic devices while ensuring user comfort. Photothermal superhydrophobic textiles are emerging as a transformative platform for self-powered thermal wearables, seamlessly integrating energy harvesting, thermal management, and sensing functionalities into everyday clothing [276–284]. These intelligent textiles leverage sunlight as a ubiquitous energy source to generate electricity through thermoelectric effects while maintaining dryness and breathability through their superhydrophobic properties [285–288]. As illustrated in Fig. 11, applications range from health monitoring sensors to adaptive clothing systems that dynamically regulate personal thermal comfort. Recent breakthroughs have demonstrated sophisticated material architectures that simultaneously optimize photothermal conversion, thermoelectric performance, and wearability, pushing the boundaries of what’s possible in autonomous wearable technology.

**Fig. 14:**
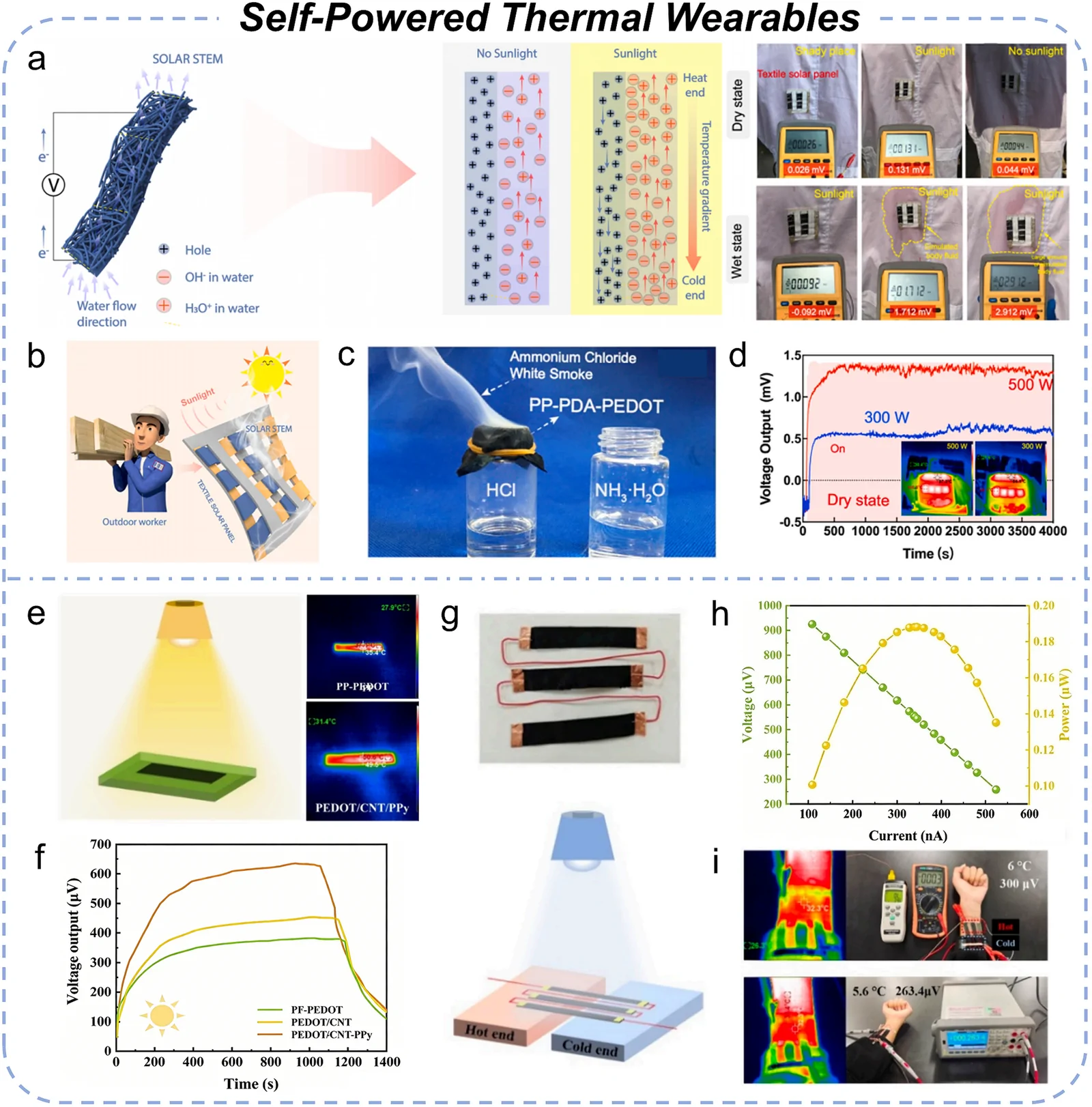
Representative research examples of self-powered thermal wearables. **a** Mechanism of electricity generation in the PP-PDA4-PEDOT and physical photograph of on-body application of dry and wet textile solar panel under sunlight [289]. **b** Protentional application of the textile solar panel [289]. **c** PP-PDA-PEDOT exhibited excellent breathability [289]. **d** Stability test of voltage output of the textile solar panels under 300 W and 500 W illumination. Reproduced with permission [289]. Copyright 2021, Elsevier. **e** Temperature increase of the fabric under simulated solar light and thermal images of PP − PEDOT, PEDOT/CNT/PPy under xenon lamp irradiation [290]. **f** Output voltage of PP − PEDOT, PEDOT/CNT, and PEDOT/CNT/PPy under simulated solar illumination [290]. **g** Schematic illustration of the assembled PEDOT/CNT/PPy thermoelectric textile device [290]. **h** Relationships between output voltage, power, and current for the PEDOT/CNT/PPy thermoelectric fabrics at Δ*T* = 30 K [290]. **i** Practical voltage measurement of the thermoelectric device attached to a human forearm under indoor ambient conditions. Reproduced with permission [290]. Copyright 2025, ACS

A pioneering development in this domain is the poly 3,4-ethylene dioxythiophene (PEDOT)-based photo-thermoelectric textile fabricated using a polydopamine (PDA)-assisted strategy [289]. This system exemplifies the concept of human body microenvironment-adaptive power generation through its innovative hierarchical structure. Figure 14a illustrates the mechanism where the textile’s bottom surface acts as a photothermal layer, reaching approximately 80 °C under infrared illumination, while the top surface maintains excellent thermoelectric properties. This creates a natural temperature gradient for electricity generation via the Seebeck effect. The textile demonstrates remarkable versatility, achieving 51.87% solar conversion efficiency under 3 sun illumination. More impressively, when the textile transitions from dry to wet state, it leverages a synergistic photo-thermoelectric and hydroelectric effect, dramatically increasing voltage output from 1.37 to 12.5 mV under 500 W illumination (Fig. 14a). This dual-functionality enables simultaneous solar energy harvesting and sweat management, as demonstrated by the textile’s ability to generate approximately 3 g of solar steam within one hour while producing usable electrical power.

The practical implementation of this technology is showcased through textile solar panels integrated into wearable formats. Figure 14b illustrates potential applications where these panels can be strategically incorporated into clothing for body humidity management. The material’s breathability, a critical factor for wearability, is convincingly demonstrated in Fig. 14c, where white smoke passes through the fabric, indicating excellent air permeability. Stability tests under intermittent illumination (Fig. 14d) confirm the reliability of these systems for real-world applications, maintaining consistent voltage output over multiple cycles. The transition between dry and wet states creates particularly promising applications for athletic wear, where increased sweating during exercise would actually enhance the electrical output rather than compromising functionality.

Complementing this approach, research on PEDOT/CNT/PPy thermoelectric fabrics demonstrates another sophisticated material architecture for wearable energy harvesting [290]. This system employs a layer-by-layer assembly to create a core–shell structure that integrates the superior photothermal properties of polypyrrole with the excellent electrical characteristics of carbon nanotubes and PEDOT. The performance comparison in Fig. 14e reveals the substantial enhancement in photothermal conversion achieved through this material design, with PEDOT/CNT/PPy reaching 50.1 °C under xenon lamp irradiation compared to 35.4 °C for PP-PEDOT alone. This efficient light-to-heat conversion directly translates to improved electrical output, as shown in Fig. 14f, where the PEDOT/CNT/PPy fabric generates a stabilized voltage of approximately 620 μV, significantly outperforming other configurations under simulated solar illumination.

The transition from material development to practical device integration is clearly demonstrated in Fig. 14g, which shows three strips of PEDOT/CNT/PPy fabric connected in series to form a functional thermoelectric generator (TEG). This device configuration represents a crucial step toward real-world applications. The power generation characteristics were systematically evaluated under a controlled temperature difference (ΔT) of 30 K, as presented in Fig. 14. The results show a classic trend where output voltage decreases with increasing load current, while output power follows a parabolic relationship. Most significantly, the device achieved a maximum output power of 0.188 nW when the external load resistance reached approximately 16 kΩ, matching the generator’s internal resistance—a fundamental principle of impedance matching for optimal power transfer in energy harvesting systems [290].

Most importantly, practical validation was conducted by attaching the 3-pair PEDOT/CNT/PPy device to a human forearm, as shown in Fig. 14i. Utilizing the natural temperature difference between skin contact (hot side) and ambient air (cold side), the device generated measurable output voltages of 300 and 263 μV under temperature differences of 6 and 5.6 °C, respectively. This real-world demonstration under indoor ambient conditions highlights the feasibility of harnessing human body heat gradients for continuous energy harvesting, eliminating the need for external power sources for low-power wearable electronics.

In summary, the systems represented in Fig. 14 demonstrate the remarkable progress in photothermal superhydrophobic textiles for self-powered wearables. The detailed device characterization in Fig. 14g, h provides crucial engineering insights into optimal operating conditions and power output capabilities. By intelligently designing material architectures that synergistically combine photothermal conversion with thermoelectric effects, these technologies can harvest energy from both sunlight and body heat while maintaining the comfort and practicality required for everyday use. The ability to generate power from environmental sources while providing thermal management and sensing capabilities positions these textiles as foundational technologies for the next generation of autonomous wearable systems in healthcare, sports, and personal electronics.

### Healthcare and Bioprotective Materials

Pathogenic microorganism colonization on protective textiles poses a significant threat to personal health and public hygiene, particularly in clinical settings. Photothermal superhydrophobic textiles offer an innovative solution for healthcare applications by integrating physical barrier functions with active sterilization capabilities. These materials operate on a synergistic “repel-and-kill” mechanism: superhydrophobic surfaces initially prevent bacterial attachment, while photothermal effects can be activated on demand to eliminate any microorganisms that breach the physical barrier. As shown in Fig. 11, these advanced materials find critical applications in surgical gowns, medical nonwovens, and protective equipment, significantly reducing the risk of healthcare-associated infections.

**Fig. 15:**
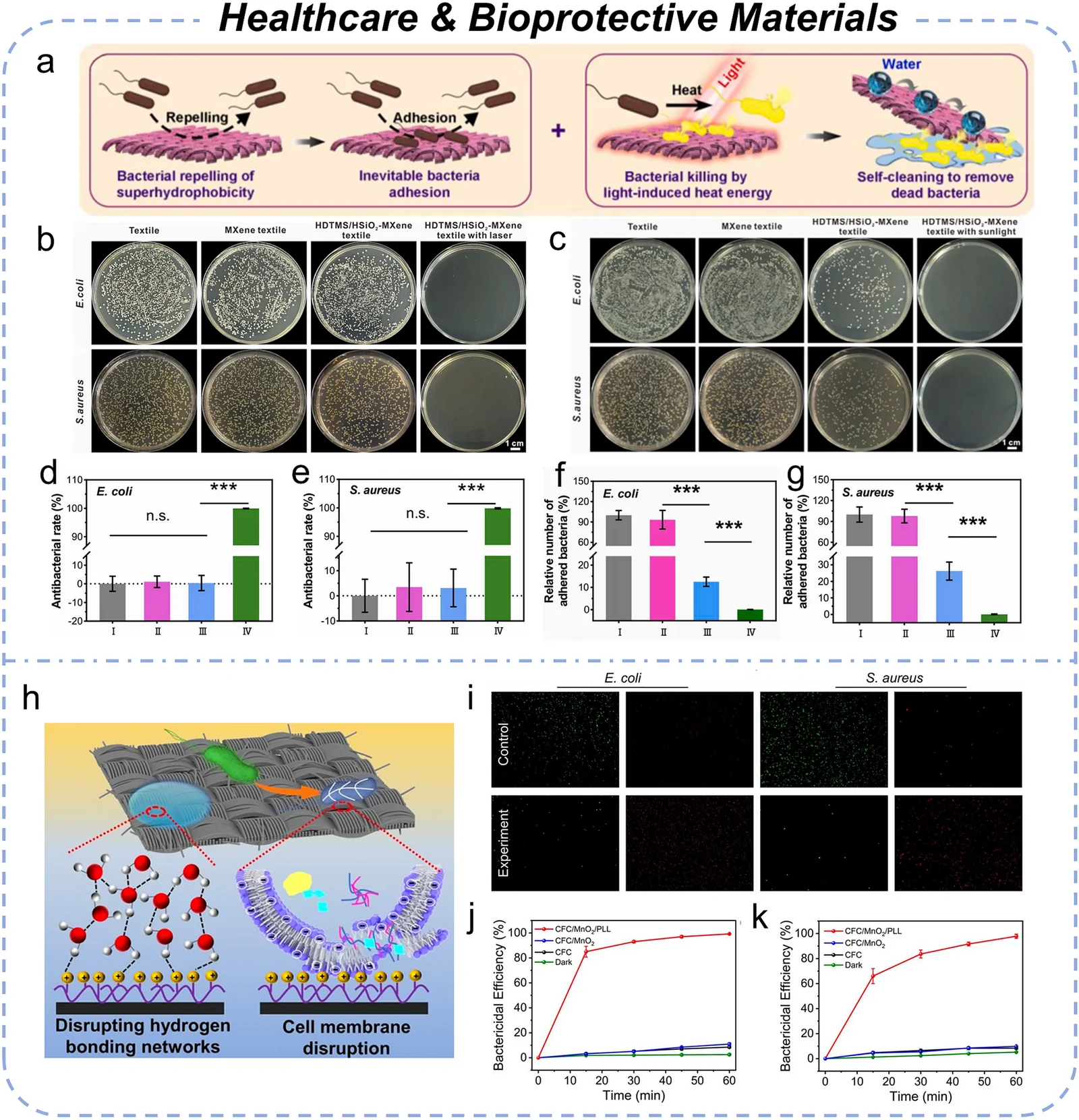
Representative research examples of healthcare and bioprotective materials. **a** Schematic illustration for antibacterial mechanism of the HDTMS/HSiO_2_-MXene textile [139]. **b** Representative photographs of the re-cultivated *E. coli* and *S. aureus* colonies on LB agar plates [139]. **c** Representative photographs of the re-cultivated *E. coli* and *S. aureus* colonies on LB agar plates [139]. **d**, **e** Antibacterial efficiency of different textiles for *E. coli* and *S. aureus*: **i** textile, (II) MXene textile, (III) HDTMS/HSiO_2_-MXene textile, and (IV) HDTMS/HSiO_2_-MXene textile with laser [139]. **f**, **g** Relative number of *E. coli* and *S. aureus* adhered to different textiles: **i** textile, (II) MXene textile, (III) HDTMS/HSiO_2_-MXene textile, and (IV) HDTMS/HSiO_2_-MXene textile with laser. Reproduced with permission [139]. Copyright 2025, Elsevier. **h** Interfacial enthalpy reduction and antibacterial mechanism of manganese oxide/poly-L-lysine co-decorated carbon-fiber cloth (CFC/MnO_2_/PLL) [291]. **i** Fluorescent live/dead stain images of *E. coli* and *S. aureus* with or without CFC/MnO_2_/PLL [291]. **j** *E. coli* and **k** *S. aureus*. Antibacterial efficiency from CFC, CFC/MnO_2_, and CFC/MnO_2_/PLL. Reproduced with permission [291]. Copyright 2024, Springer Nature

A representative example is the hexadecyltrimethoxysilane (HDTMS)/HSiO_2_-MXene textile, which demonstrates a dual-functional antibacterial approach [139]. Figure 15a illustrates the cooperative antimicrobial mechanism: the superhydrophobic surface creates a physical barrier through trapped air layers in the Cassie-Baxter state, significantly reducing direct microbe-textile contact. When microorganisms eventually penetrate this barrier over prolonged exposure, the MXene layer enables photothermal sterilization, generating localized temperatures exceeding 110 °C under near-infrared irradiation to destroy bacterial cellular structures.

The antibacterial efficacy is validated through standard colony culture experiments. Figure 15b, c shows that pristine textile, MXene textile, and HDTMS/HSiO_2_-MXene textile exhibit minimal inherent antibacterial activity. However, when the HDTMS/HSiO_2_-MXene textile receives 15 min of 808-nm NIR irradiation, antibacterial efficiency exceeds 99.8% against both *E. coli* and *S. aureus* (Fig. 15d, e). Quantitative analysis further confirms massive protein leakage from thermally disrupted bacterial membranes.

The anti-adhesion capability of the superhydrophobic surface is quantified in Fig. 15f, g, showing a 75% reduction in bacterial attachment compared to untreated textiles. This initial protection effectively prevents biofilm formation during routine use. Notably, even after 72 h of co-culture when some bacteria penetrate the hydrophobic barrier, exposure to simulated solar irradiation completely eliminates residual bacteria, ensuring long-term biological cleanliness.

Complementing this approach, the carbon-fiber cloth (CFC)/MnO_2_/PLL system demonstrates an alternative strategy using superhydrophilic design for advanced applications [291]. Figure 15h reveals its unique interfacial antibacterial mechanism, where the combination of manganese oxide nanosheets and poly-l-lysine reduces water evaporation enthalpy to 2132.34 kJ kg⁻^1^ while enabling efficient bacterial inactivation through electrostatic interactions with cell membranes. This design transforms hydrophobic carbon-fiber cloth into a superhydrophilic material with 97.8% broadband light absorption, making it particularly suitable for applications requiring rapid water transport.

The antibacterial performance of CFC/MnO_2_/PLL is vividly demonstrated through fluorescence live/dead staining in Fig. 15i, where most bacteria show red fluorescence (dead) compared to predominantly green fluorescence (live) in control groups. Quantitative antibacterial assays in Fig. 15j–k further confirm that CFC/MnO_2_/PLL achieves exceptional antibacterial efficiencies of 99.1% against *E. coli* and 98.2% against *S. aureus* within 60 min in darkness, significantly outperforming CFC alone or CFC/MnO_2_ composites.

These complementary systems highlight the versatility of photothermal textiles in healthcare applications. The HDTMS/HSiO_2_-MXene approach provides optimal protection for dry environments through its superior anti-adhesion properties, while the CFC/MnO_2_/PLL system offers advantages in applications requiring fluid management through its superhydrophilic characteristics. Both systems demonstrate how intelligent material design can create adaptive protective textiles that maintain sterility through different mechanistic pathways, advancing the development of next-generation biomedical materials for infection control.

### Other Applications

**Fig. 16:**
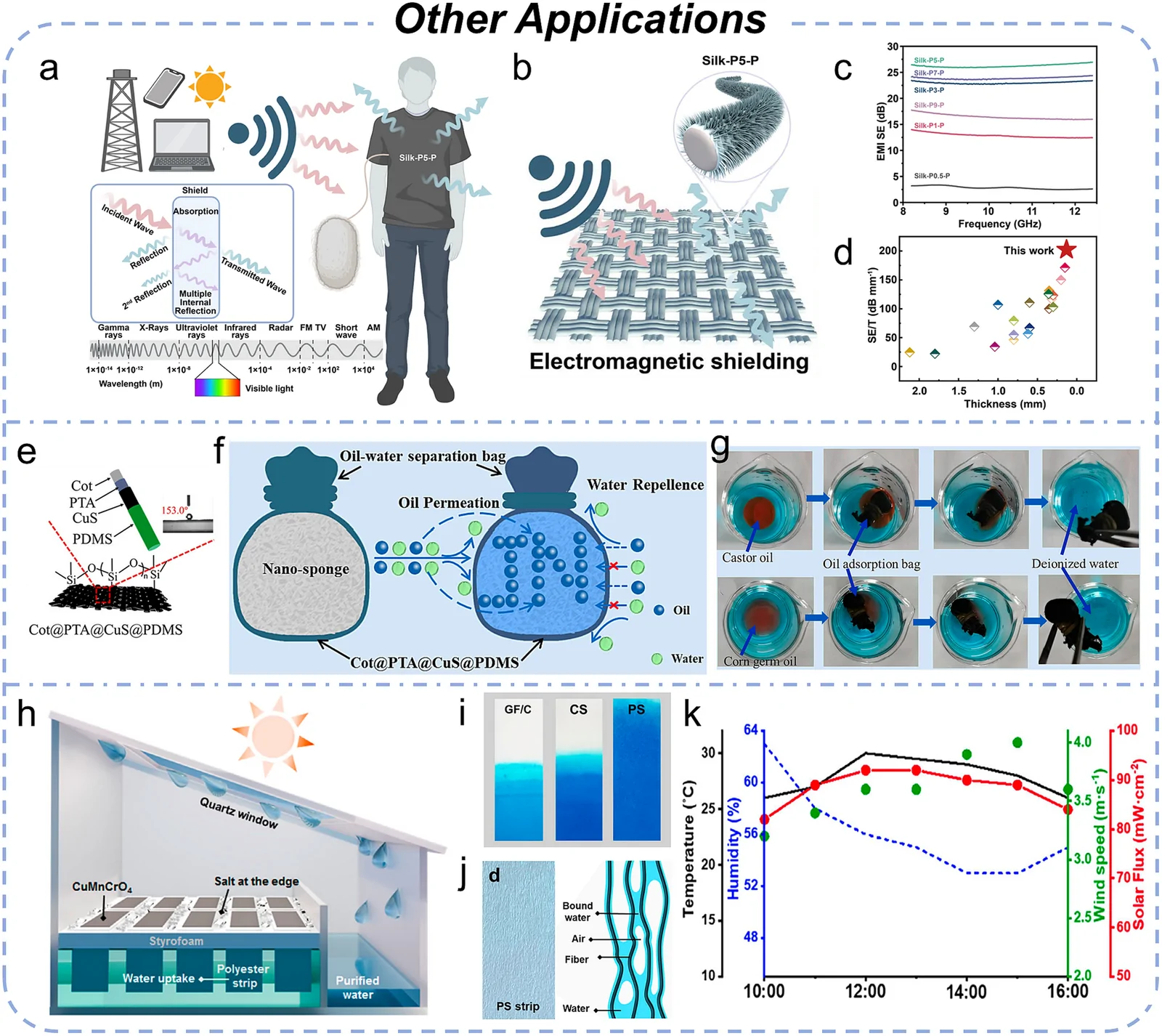
Representative research examples of other applications. **a** Mechanisms for effective electromagnetic shielding by Silk-P5-P [171]. **b** Schematic diagram of the structure of Silk-P5-P fabric [171]. **c** Comparison of EMI-shielding effect of Silk-P (0.5–9.5)-P with different py ratios for X-band (8.2–12.4 GHz) [171]. **d** Comparison of Silk-P5-P with recently published studies on EMI-shielding fabrics in terms of thickness and EMI SE per unit thickness. Reproduced with permission [171]. Copyright 2026, Springer Nature. **e** Schematic diagram of photothermal, superhydrophobic, conductive, and UV-resistant composite coating on cotton fabric [166]. **f** Simulated diagram of the oil–water separation bag in the separation of oil–water mixture [166]. **g** Castor oil and corn germ oil. The castor oil and corn germ oil were dyed by Sudan red G. The deionized water was dyed by Reactive Blue 19 (20 mg L^−1^). Reproduced with permission [166]. Copyright 2024, Elsevier. **h** Schematic description of solar evaporation system [301]. **i** Comparison of the water wicking abilities of GF/C filter paper, cotton strip (CS), and polyester strip (PS) [301]. **j** Schematic description of the water wicking process of PS [301]. **k** Comparison of the water evaporation rate of CMCO with reported photothermal materials. Reproduced with permission [301]. Copyright 2025, Wiley

Beyond the primary domains of thermal management, anti/de-icing, and biomedical protection, photothermal superhydrophobic textiles also show potential in EMI shielding (Fig. 16a–d), oil–water separation, solar desalination, and other emerging scenarios [14, 169, 171, 247, 269, 292–300]. In these applications, multifunctionality is usually achieved by coupling the water-repellent textile surface with an additional functional network. For EMI shielding, conductive components such as PPy, Ag nanowires, MXene, or carbon-based materials attenuate electromagnetic waves through reflection, absorption, and multiple internal scattering, while the superhydrophobic coating protects the conductive network against water-induced degradation. For oil–water separation and solar desalination, the low-adhesion surface controls liquid transport and fouling resistance, whereas photothermal components provide localized heating for viscosity reduction, evaporation, or interfacial energy conversion. Therefore, these “other applications” should be understood not as independent functions, but as extensions of the same photothermal superhydrophobic design logic.

In the realm of oil–water separation, photothermal superhydrophobic textiles offer an efficient and sustainable solution for addressing water contamination from oil spills. A green fabrication approach is employed, where laccase-enzymatic polymerization of tannic acid facilitates the in situ deposition of CuS nanoflowers onto cotton fibers (Fig. 16e), followed by PDMS modification to achieve robust superhydrophobicity [185]. The resulting fabric (cotton@PTA@CuS@PDMS) is engineered into an innovative oil–water separation bag by packaging a nano-sponge core. As depicted in Fig. 16f, the superhydrophobic cotton shell selectively repels water while allowing oil permeation, driven by the low surface energy of PDMS methyl groups. When deployed in oil–water mixtures (Fig. 16g), the bag efficiently separates oils with remarkable efficiency (98.5%–98.7%), demonstrating excellent practical applicability.

Solar desalination has emerged as another promising application, addressing global freshwater scarcity through sustainable solar energy utilization. An innovative inverted U-shaped dual water transport structure was developed to overcome the limitations of conventional single-inlet systems, which often suffer from insufficient water supply and salt accumulation on the photothermal material (PTM) surface [301]. As shown in Fig. 16h, the system utilizes hydrophobic polyester nonwoven strips (PS) as the wicking material, which, despite the inherent hydrophobicity of polyester, enables efficient capillary water transport through its interconnected microfibrous structure. This design achieves a record-high evaporation rate of 4.1 kg m^−2^ h^−1^ under 1.0 sun irradiation while maintaining stable performance over three weeks without salt accumulation. The superior wicking capability of PS is demonstrated in Fig. 16i, where it outperforms both cotton strip (CS) and GF/C paper in capillary transport rate. The mechanism of water movement along PS fibers through adhesion and cohesion forces is illustrated in Fig. 16j, ensuring continuous water supply to the PTM-coated cotton surface while effectively directing salt away from the evaporation zone.

These diverse applications underscore the versatility of photothermal superhydrophobic textiles beyond conventional uses. The integration of EMI-shielding capabilities with self-cleaning properties opens new avenues for smart protective wearables. Simultaneously, the development of efficient oil–water separation and solar desalination systems highlights their potential contribution to environmental remediation and sustainable water management. The ability to tailor material compositions and surface architectures for specific functions, whether for signal management, selective filtration, or solar water purification, demonstrates the adaptable nature of this advanced textile platform, positioning it as a key enabling technology for future multi-functional material systems addressing critical global challenges.

The diverse applications discussed underscore photothermal superhydrophobic textiles as a versatile platform technology. Table 3 provides a consolidated overview, linking key material parameters (WCA, SA, substrate, synthesis method) to application performance. The data reveal critical design paradigms: for instance, in situ growth on robust fabrics suits protective gear, while spray-coated nanomaterials on porous substrates excel in solar evaporation due to enhanced light absorption and water transport [297, 302, 303]. The synthesis method (e.g., LbL assembly, covalent grafting) directly dictates functional durability and application efficacy. However, the table highlights a scarcity of cross-domain studies, indicating a key research gap: systematically evaluating a single optimized material across different applications—from wearables to environmental engineering—to establish universal design principles and accelerate commercial translation [236, 237, 304, 305].

**Table 3:** The summary of photothermal superhydrophobic textiles for broad applications

Fabric system (reference)	WCA (°)	SA (°)	Substrate	Synthetic method	Applications	Application Performance	Refs.
FAS/Ag/MXene@PLA	151.5	NA	PLA nonwoven fabric	Dip-coating	Anti/de-icing Antibacterial	Anti/de-icing: delay 11.2 min @ − 10 °C; deicing 40 s @ 10 μL ice/1.0 sun. Antibacterial: 99.99% @ 1.0 sun/20 min	[244]
PDMS/Fe_3_O_4_/PPy@PET	152	NA	PET	In situ growth Spray	Anti/de-icing	Anti/de-icing: delay 181 s @ − 10 °C; melting 841 s @ 3 mm ice/ − 10 °C/1.0 sun	[25]
ZIF-8/PPy@cotton	167	2	Cotton	In situ growth	Anti/de-icing	Anti/de-icing: delay 442.7 s @ 10 μL/ − 20 °C; delay 651.9 s @ 0.5 sun; deicing 250 s @ 10 μL ice/1.0 sun	[33]
HDTMS/HSiO_2_/MXene @ Cotton	154	5.2	Cotton	Dip-coating	Antibacterial	Antibacterial: > 99.8% against E. coli and S. aureus. Photothermal: 60.5 °C @ simulated solar irradiation	[139]
PDMS/ CuS/ PTA@Cotton	153	NA	Cotton	Salt templating In situ growth	Oil–water separation UV protection	Oil–water separation: > 98.5% for oil-in-water emulsions; > 98.7% for surfactant-stabilized water-in-oil emulsions. UV protection: UPF 394.74 after 5 washing cycles	[184]
PDMS/Ppy @Silk	155.5	4.2	Silk	Sequential coating	Antibacterial EMI shielding	Antibacterial: > 99% @ 808 nm NIR. EMI shielding: 25.4 dB single layer; 62.9 dB triple layer	[171]
Mx@SPF (asymmetric Janus PET)	160	NA	Polyester	Dip-coating	EMI shielding Thermal management	EMI shielding: 8–36 dB after 1–7 coating cycles; 42/53 dB for 2/3 layers. Anti/de-icing: deicing 566 s @ 1 cm^2^ × 2 mm ice/1.0 sun	[245]
PDMS/PPy/TiN@Cotton	156	NA	Cotton	Dip‐coating	Oil–water separation	Oil–water separation: > 98.0%; > 97.0% after 10 cycles; flux decline 8.0%	[306]
PDMS/Ag@Cotton	171.31	NA	Cotton	Dip‐coating	Antibacterial UV protection	Antibacterial: CFU reduction 86.11%. UV protection: UPF 215–219; UVA/UVB transmittance reduced by 93.83–95.56%/70.5–71.42%	[93]
PDMS/PPy/ MXene@Cotton	152	7	Cotton	Dip‐coating, in situ	Anti/de-icing	Anti/de-icing: ice removal 165 s @ 350 μL ice/sunlight	[137]
PDMS/Ag/GA/ PEI/PDA@ @Cotton	159.6	NA	Cotton	Dip‐coating	Antibacterial	Photothermal antibacterial: 70.4 °C @ 20 A + simulated solar irradiation	[242]
PDMS/Ag/PDA/CuS@Cotton	154.5	NA	Cotton	Dip‐coating	Antibacterial	Antibacterial: > 99.9% @ 0.5 W cm − 2 NIR/1 min	[191]
SH/CA-Fe@PET	156	3.3	PET	In situ growth	Oil–water separation UV protection	Oil–water separation: flux > 1200 L m^−2^ h ^−1^ after 30 cycles; efficiency > 95% after 30 cycles. UV protection: UPF 95.98	[307]
HDTMS/CA/Fe@PET	161.3	4	PET	Dip‐coating	Oil–water separation	Oil–water separation: efficiency > 95% after 10 cycles; high flux retention	[308]
APTES-ODA/PPy@PET	155.8	NA	PET	In situ growth	UV protection	UV protection: two absorption peaks at 200–350 nm; absorbance increased by 135% over 200–800 nm	[309]
PDMS/TiN@Cotton	159	NA	Cotton	Dip‐coating	Anti/de-icing	Anti/de-icing: ice removal ~ 50 s @ 25 °C/1.0 sun	[105]
PDMS/Ag-SMNPs@Cotton	156	3.3	Cotton	Dip‐coating	Antibacterial UV protection	Antibacterial: viability 1.94% for E. coli and 5.92% for S. aureus @ 10 min irradiation. UV protection: UPF > 50	[310]

## From Lab to Life: Translating Photothermal Superhydrophobic Textiles to Practical Applications

The transition of photothermal superhydrophobic textiles from academic research to real-world applications requires addressing fundamental challenges that extend beyond material performance. This section examines the key factors governing their successful integration into daily life, focusing on functional synergy, long-term durability, and environmental compatibility—all critical for gaining industry acceptance and consumer trust.

### Multifunctional Synergy: Beyond Single-Mode Performance

**Fig. 17:**
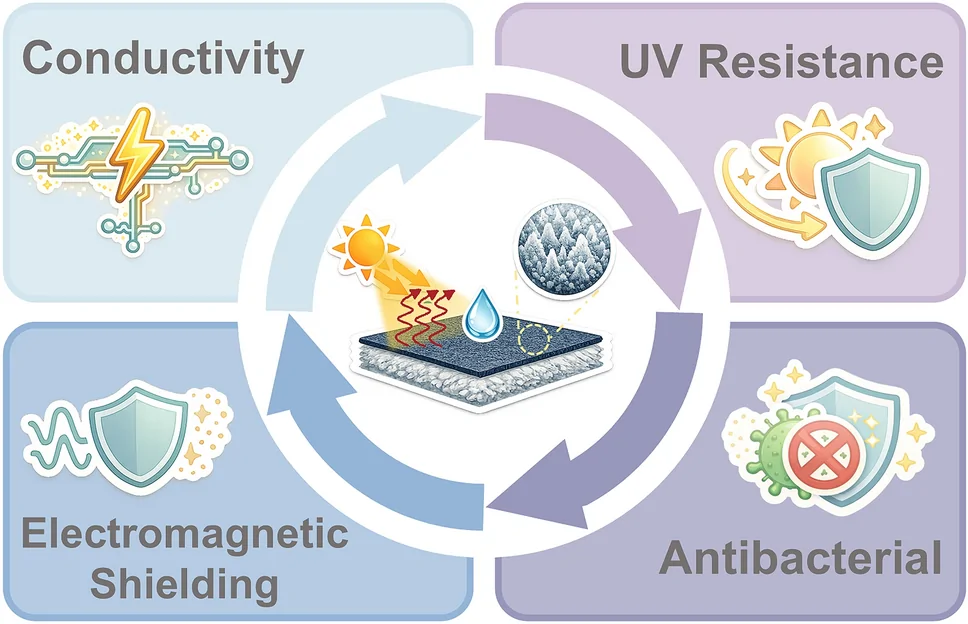
Multifunctional synergistic design of photothermal superhydrophobic textiles

While individual properties (e.g., superhydrophobicity, photothermal conversion) have been extensively optimized in the laboratory, the practical value of these textiles lies in their ability to integrate multiple functions without performance trade-offs. Figure 17 illustrates the core multifunctional synergies of modern photothermal superhydrophobic textiles, encompassing conductivity, UV resistance, electromagnetic interference (EMI) shielding, and antibacterial activity—all of which are critical for real-world applications ranging from wearable electronics to outdoor gear. These integrated functionalities are not merely additive but create emergent properties that address unmet needs in diverse sectors.

**Fig. 18:**
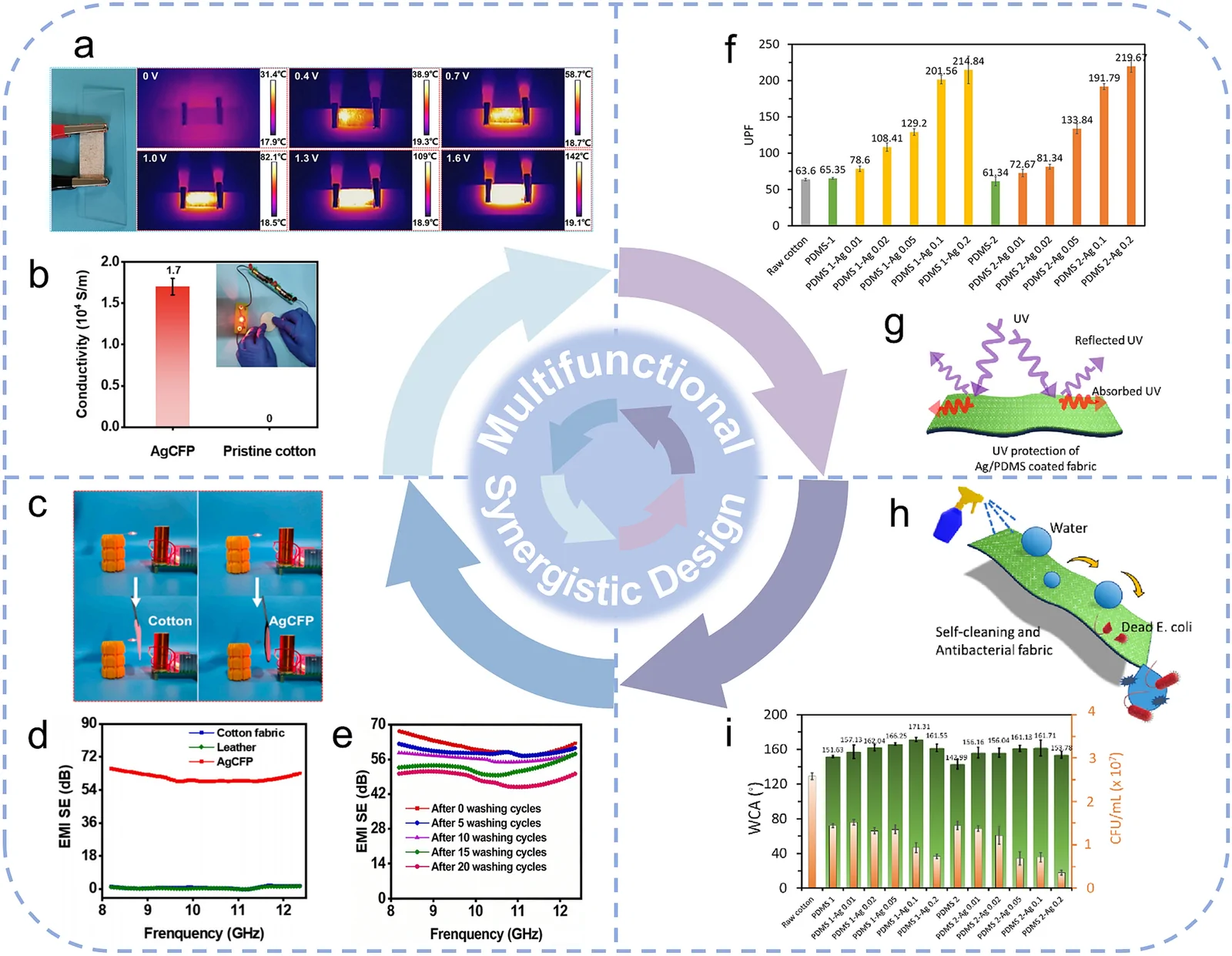
Representative research examples of multifunctional synergistic design of photothermal superhydrophobic textiles. **a** IR images of AgCFP under different driving voltage [222]. **b** Conductivity of AgCFP [222]. **c** Digital images of Tesla coil measurement for the EMI-shielding capacity of the pristine cotton and AgCFP [222]. **d** EMI SE of the pristine cotton, leather, and AgCFP [222]. **e** EMI SE of AgCFP after washing. Reproduced with permission [201]. Copyright 2026, Elsevier. **f** UPF level of raw and coated fabrics [93]. **g** Schematic illustration of UV protection of fabric coated with Ag/PDMS [93]. **h** Schematic illustration of mechanisms of superhydrophobic property and antibacterial activity on cotton fabric coated with Ag/PDMS [93]. **i** Variations of WCA and measured CFU levels. Reproduced with permission [93]. Copyright 2023, ACS

A prime example of this synergy is the AgCFP (silver nanoparticle-coated cotton fabric), which demonstrates exceptional multifunctional performance [222]. Figure 18b highlights its remarkable conductivity (≈1.7 × 10^4^ S m^−1^), significantly higher than pristine cotton, enabling it to function as a wire to illuminate an LED bulb—a critical attribute for wearable heating applications. The electric heating performance of AgCFP, governed by Joule’s law (*Q* = *U*^2^/*R* × *t*), is vividly depicted in Fig. 18a: under driving voltages from 0.4 to 1.6 V, the surface temperature ranges from 38.3 to 141.3 °C, with uniform heat distribution confirmed by infrared imaging. This allows AgCFP to provide continuous warmth for the human body, making it ideal for cold-weather apparel [222]. Beyond thermal management, AgCFP exhibits outstanding EMI-shielding capabilities, a growing concern with the proliferation of 5G/6G technology [222]. Figure 18d shows that AgCFP achieves an EMI-shielding effectiveness (SE) of ≈ 61 dB—far exceeding the commercial standard of 20 dB—effectively blocking 99.9999% of electromagnetic waves. A Tesla coil experiment (Fig. 18c) visually confirms this: placing AgCFP between a Tesla coil and an LED bulb extinguishes the bulb, whereas pristine cotton leaves it lit, demonstrating AgCFP’s ability to attenuate electromagnetic radiation through internal reflections and absorptions in its dense conductive network. Importantly, Fig. 18e shows that AgCFP retains its EMI SE after 20 washing cycles, underscoring its durability for long-term use.

Complementing AgCFP, Ag/PDMS-coated cotton fabrics exemplify the integration of superhydrophobicity with UV resistance and antibacterial activity [93]. Figure 18f reveals that Ag NPs content directly enhances the UV protection factor (UPF): raw cotton (UPF ≈ 63.6) is transformed into fabrics with UPF values up to ≈219, reducing UVA/UVB transmission by 95.56% and 71.42%, respectively. Figure 18g illustrates the mechanism: Ag NPs absorb incident UV light, while PDMS forms a protective layer, synergistically shielding wearers from harmful radiation. These examples indicate that multifunctional photothermal superhydrophobic textiles are best understood as integrated material systems rather than simple combinations of independent functions. In Ag- or PPy-based designs, metallic or conjugated components can simultaneously contribute to light absorption, electrical conductivity, UV attenuation, and antibacterial activity, while PDMS or other low-surface-energy coatings stabilize the water-repellent interface and protect the functional network from wetting-induced degradation. Such shared structural and chemical features explain why thermal management, antibacterial protection, EMI shielding, and UV resistance often emerge together in a single textile platform.

From an integrative design perspective, the multifunctionality of photothermal superhydrophobic textiles should be optimized according to application-specific trade-offs rather than maximized indiscriminately. Antibacterial textiles require sufficient localized heating or biocidal components while maintaining skin-compatible temperature windows and washing durability. EMI-shielding textiles rely on continuous conductive networks, but excessive conductive loading may compromise breathability, softness, and coating adhesion. Personal thermal management textiles require efficient light-to-heat or electrothermal conversion, yet overheating and poor moisture permeability must be avoided for wearable comfort. Therefore, the central design challenge is not simply to accumulate photothermal, antibacterial, EMI-shielding, UV-protective, and self-cleaning functions in one material, but to balance light absorption, electrical conductivity, surface adhesion, air/moisture permeability, mechanical compliance, and long-term durability for the targeted service environment.

### Durability: Sustaining Performance Under Real-World Stress

**Fig. 19:**
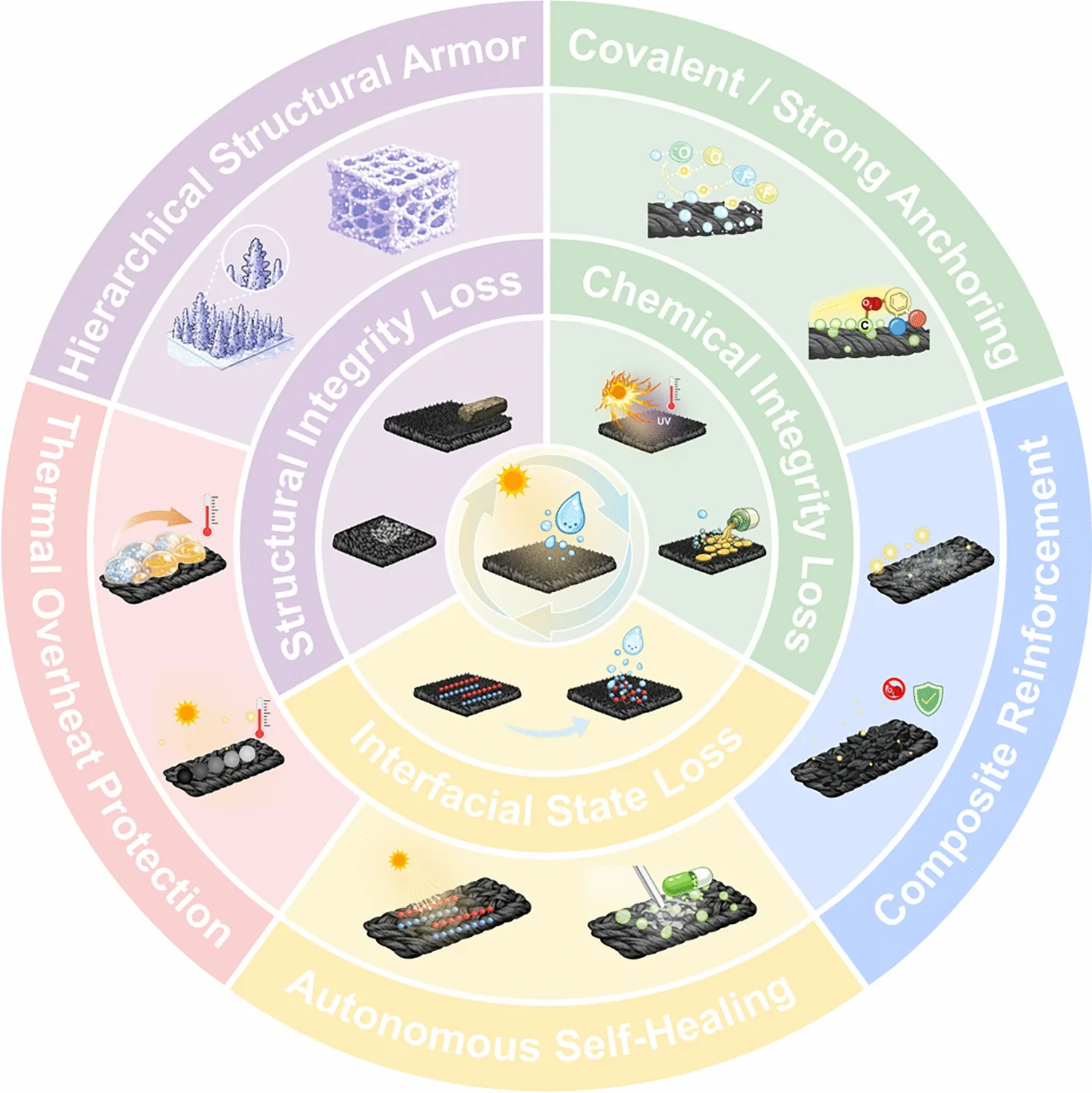
Durability-oriented design framework for photothermal superhydrophobic textiles: failure pathways and corresponding reinforcement strategies

The Achilles’ heel of many laboratory-developed superhydrophobic materials is their poor durability—surface treatments often degrade after mechanical abrasion, chemical exposure, or UV irradiation, rendering them ineffective in practical use. For photothermal superhydrophobic textiles, two critical durability metrics must be evaluated: mechanical robustness, defined as resistance to friction (e.g., washing, rubbing), stretching, and folding, and functional stability, which refers to the retention of superhydrophobicity (water contact angle > 150°, sliding angle < 10°) and photothermal conversion efficiency after prolonged exposure to environmental stressors. To systematically address these challenges, Fig. 19 presents a durability-oriented design framework that categorizes failure pathways into three core types: structural integrity loss (e.g., abrasion-induced damage to hierarchical roughness), chemical integrity loss (e.g., UV degradation of low-surface-energy coatings), and interfacial state loss (e.g., weak adhesion between coatings and substrates). Corresponding reinforcement strategies include hierarchical structural armor, covalent/strong anchoring, composite reinforcement, autonomous self-healing, and thermal overheat protection, which collectively enhance material longevity by targeting specific failure mechanisms.

**Fig. 20:**
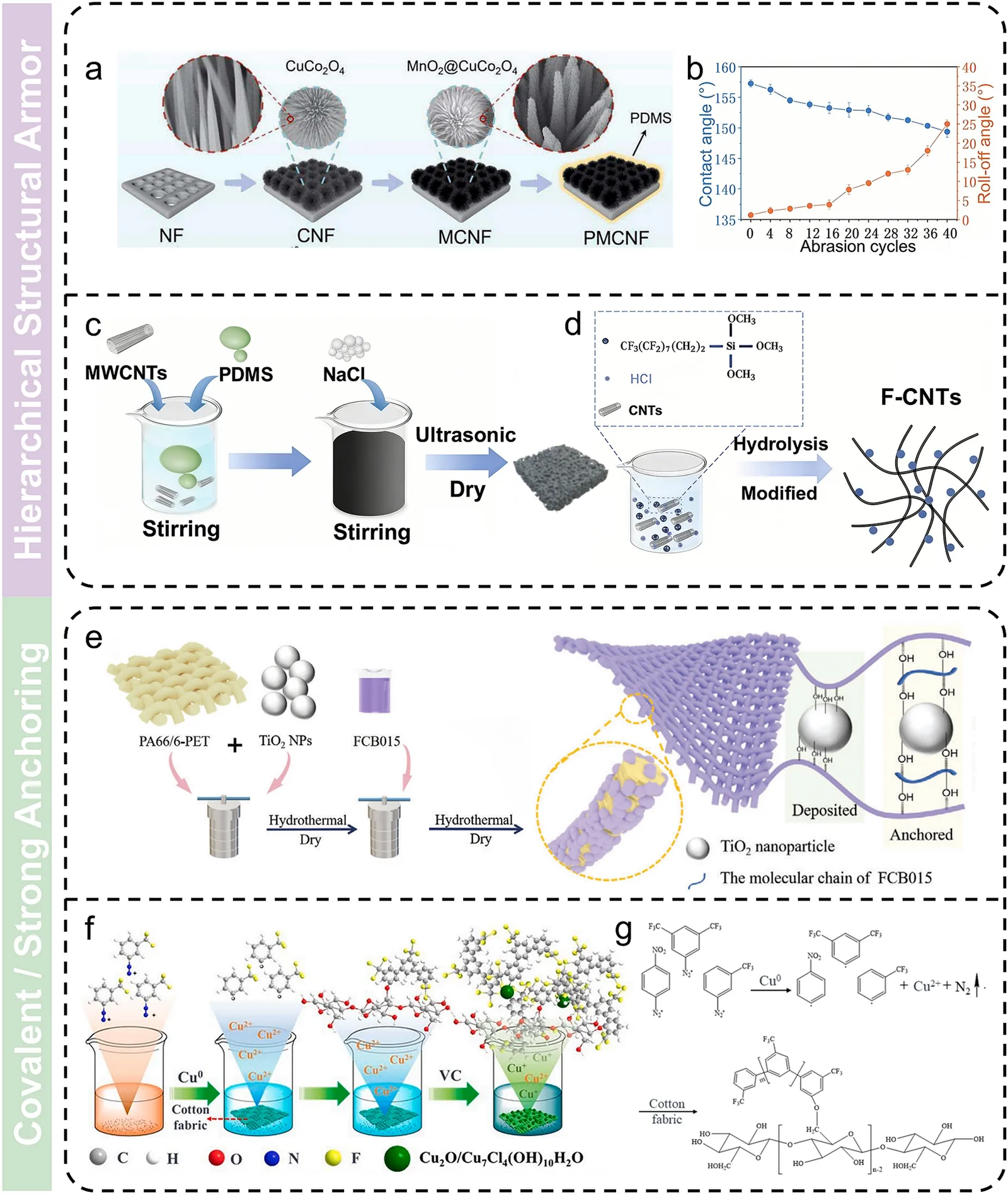
Representative research examples of hierarchical structural armor and covalent/strong anchoring. **a** A schematic illustration of the PMCNF preparation. The MnO_2_@CuCo_2_O_4_ composite exhibits a hierarchical nano-/microstructure [311]. **b** 40 cycles of sandpaper abrasion on PMCNF. Reproduced with permission [311]. Copyright 2025, Elsevier. Schematic diagram of preparation process of **c** porous PDMS/CNTs (PC); **d** FAS-modified CNTs. Reproduced with permission [129]. Copyright 2025, Elsevier. **e** Schematic diagram of the PA66/6-PET@Tico preparation process, including two-step hydrothermal treatment and intermolecular action mechanism diagram of coating. Reproduced with permission [312]. Copyright 2024, Wiley. **f** Schematic illustration of the fabrication of CF_3_phene-Cu_2_O/Cu_7_C_l4_(OH)_10_H_2_O@cotton fabric (CF_3_Ph-Cu_x_O@CF) [313]. **g** Radical mechanism for the grafting of nitrobenzene diazonium, 3-(trifluoromethyl) benzene diazonium or 3,5-di(trifluoromethyl)benzene diazonium salts on the surface of cotton fabrics via in situ diazonium radical polymerization. Reproduced with permission [313]. Copyright 2022, Elsevier

A primary failure pathway for superhydrophobic textiles is the loss of hierarchical roughness due to mechanical abrasion, which disrupts the Cassie-Baxter state critical for superhydrophobicity. Hierarchical structural armor—designing multi-scale (nano/micro) surface topographies—effectively mitigates this by distributing external forces across multiple structural levels, preserving critical roughness even after partial damage. A representative example is the PMCNF (MnO_2_@CuCo_2_O_4_/Ni foam) material, which features a dual-tier hierarchical structure: dandelion-like CuCo_2_O_4_ microstructures (≈20 μm diameter) decorated with MnO_2_ nanowires, coated with PDMS for low surface energy (Fig. 20a) [311]. To evaluate mechanical durability, PMCNF underwent 40 cycles of sandpaper abrasion (100 g weight, 10 cm displacement per cycle). As shown in Fig. 20b, the water contact angle (WCA) remained above 150° up to 36 cycles, and the roll-off angle (RA) stayed below 10° for 28 cycles—far exceeding typical lab-scale materials. Notably, the RA increased more rapidly than WCA decreased, attributed to modest solid–liquid interface expansion and surface homogeneity deterioration, highlighting the importance of structural uniformity in sustaining superhydrophobicity. Complementing PMCNF, porous PDMS/CNT (PC) materials use a template-based strategy to create robust hierarchical structures (Fig. 20c) [129]. By embedding CNTs into porous PDMS (prepared via NaCl template leaching), the material gains both mechanical resilience and photothermal activity. To prevent nanoparticle agglomeration, CNTs and SiO_2_ are modified with 1H,1H,2H,2H-perfluorodecyltrimethoxysilane (FAS) (Fig. 20d), ensuring uniform dispersion and long-term stability. This design not only enhances mechanical durability but also retains photothermal conversion efficiency by maintaining a continuous conductive network.

Another critical failure pathway is interfacial state loss—weak adhesion between coatings and substrates that leads to peeling or delamination, especially under repeated mechanical stress. Covalent/strong anchoring strategies address this by forming chemical bonds (e.g., covalent, hydrogen bonding) between functional layers, ensuring coatings remain intact under stress. The PA66/6-PET@Tico fabric exemplifies this approach, prepared via a two-step hydrothermal method (Fig. 20e) [312]. First, TiO_2_ nanoparticles are deposited on smooth fiber surfaces to form a base coating. Then, fluorinated acrylate polymer (FCB015) molecules—containing six CF_n_ units (environmentally friendly, non-toxic)—anchor to TiO_2_ via hydrogen bonding, promoting layer-by-layer growth of multi-tentacled papillary micro-/nanostructures. At a TiO_2_/FCB015 weight ratio of 1:10, the fabric achieves exceptional superhydrophobicity (WCA = 172.2°, oil contact angle (OCA) = 166.8°), outperforming previously reported superhydrophobic textiles. The strong interfacial bonding ensures the coating resists washing and abrasion, as hydrogen bonds tightly bind the hierarchical structure to the substrate. Similarly, CF_3_Ph-Cu_x_O@CF (cotton fabric) uses in situ diazonium radical polymerization to covalently graft fluorinated benzene radicals onto cotton fibers and Cu_*x*_O nanoparticles (Fig. 20f, g) [313]. The radical mechanism (Fig. 20g) involves reducing 3-(trifluoromethyl)benzene diazonium salt with Cu powder to form reactive radicals, which then covalently bond to the fabric surface. This covalent anchoring creates a robust, fluorinated coating that retains superhydrophobicity after 50 washing cycles, as the chemical bonds prevent coating detachment.

While individual strategies (e.g., hierarchical armor, covalent anchoring) enhance durability, their combination often yields synergistic effects. For example, the PMCNF material integrates hierarchical structural armor (MnO_2_@CuCo_2_O_4_) with PDMS coating (low surface energy and chemical protection), resisting both mechanical abrasion and UV degradation. Similarly, the PA66/6-PET@Tico fabric combines hierarchical papillary structures with hydrogen bonding, addressing both structural and interfacial failure pathways. These advancements address a key gap between laboratory performance and real-world requirements, where textiles must withstand years of use without losing functionality. By systematically targeting failure pathways and implementing multi-pronged reinforcement strategies, photothermal superhydrophobic textiles are becoming increasingly viable for long-term applications in harsh environments.

**Fig. 21:**
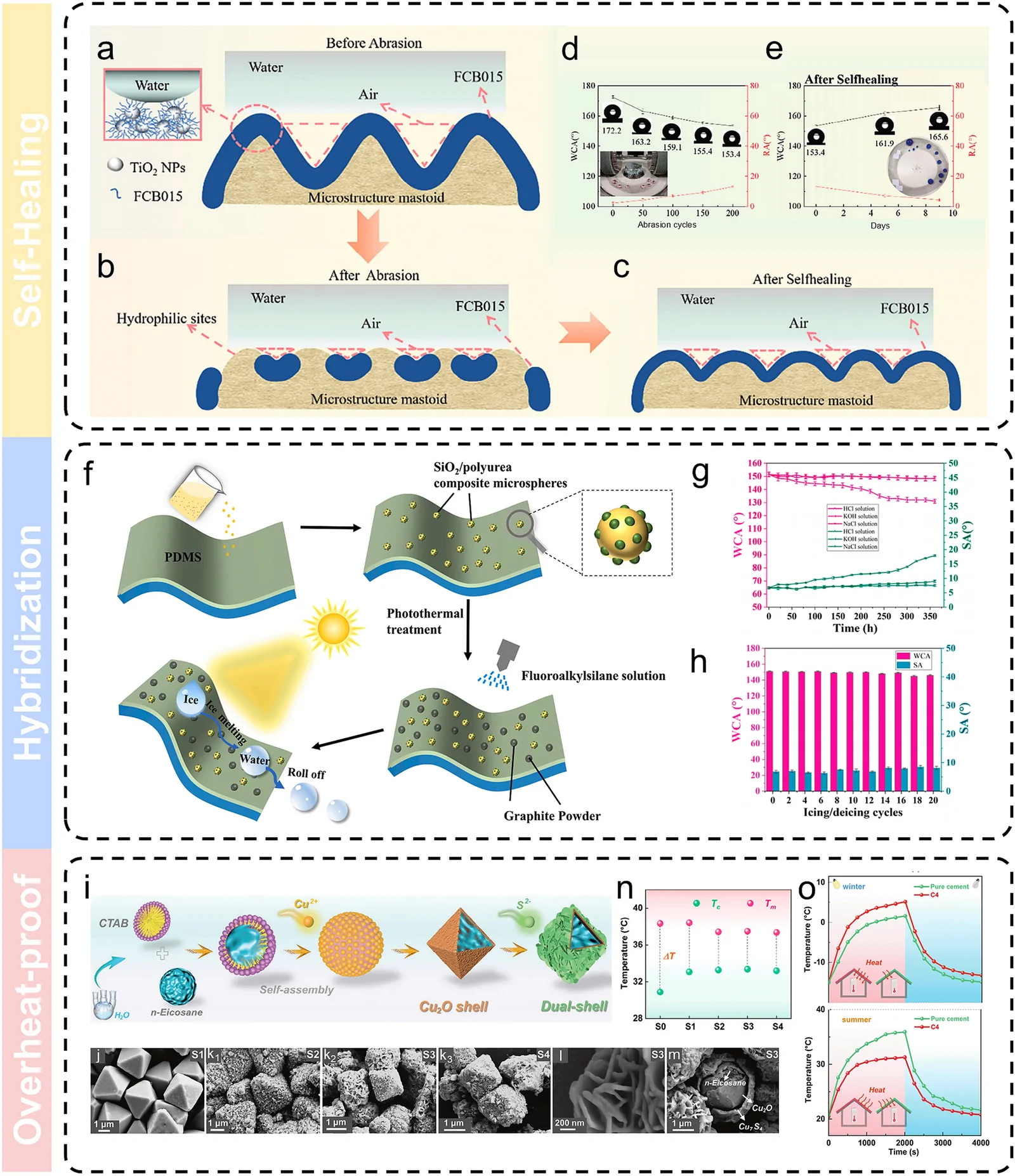
Representative research examples of autonomous self-healing, composite reinforcement, and overheat-proof. **a-c** Schematic diagram of the superhydrophobic self-healing mechanism after abrasion [312]. **d** WCA and RA change with Martindale abrasion cycles on the coated fabric, the inset shows the hydrophobic state of the fabric surface after 200 r of Martindale abrasion [312]. **e** Curve of WCA and RA versus room temperature self-healing after Martindale abrasion, the inset shows the hydrophobic state of the fabric surface after 9 days of self-healing at room temperature. Reproduced with permission [312]. Copyright 2024, Wiley. **f** Schematic illustration of the preparation process for ST@PUa/C photothermal superhydrophobic coating [315]. **g** WCA and SA of the superhydrophobic coating after immersing in the HCl (pH = 1), KOH (pH = 14) and NaCl (3.5 wt%) solution for 360 h [315]. **h** WCA and SA as a function of the icing-deicing cycles of ST@PUa/C. Reproduced with permission [315]. Copyright 2024, Elsevier. **i** Schematic diagram for preparing the dual-shell microcapsules [317]. **j** Morphologies of n-Eicosane@Cu_2_O (S1) [317]. **k**_**1**_**–k**_**3**_ Morphologies of S2, S3, and S4 [317]. **l** High-resolution nanostructures of S3 shell surface [317]. **m** SEM micrograph of the crushed S3 [317]. **n** Solidification (*T*_c_) and melting (*T*_m_) temperatures of S0–S4. The differences between *T*_m_ and *T*_c_ indicate the supercooling degrees (ΔT) [317]. **o** Indoor temperature changes of model houses in winter (− 15 °C) and summer (20 °C) after coating pure cement and a mixture of cement and 17% S3 (i.e., C4), respectively. Reproduced with permission [317]. Copyright 2024, Wiley

Beyond the fundamental reinforcement strategies, advanced functional designs are further enhancing the practical durability of photothermal superhydrophobic textiles, as exemplified by the innovative approaches summarized in Fig. 21. These strategies move beyond passive damage resistance to incorporate active recovery mechanisms and multi-functional protection. A particularly promising direction is autonomous self-healing, which enables materials to recover their superhydrophobicity after mechanical damage. Research has demonstrated at least two effective mechanisms. The first, illustrated in Fig. 21a–c, utilizes a bio-inspired system where TiO_2_ papillae and a fluorinated acrylate polymer (FCB015) coating enable remarkable room-temperature self-healing capability [312]. After Martindale abrasion tests (Fig. 21d), the WCA decreases from 172.2° to approximately 155.4°, but recovers to 165.6° after 9 days at room temperature (Fig. 21e). This recovery is driven by the molecular chain motility of the thermoplastic elastomer FCB015, where the minimization of surface free energy prompts the migration of fluorinated alkyl chains to reconstruct the low-surface-energy layer at damaged sites [312].

Additionally, an alternative self-healing approach employs pre-embedded low-surface-energy capsules, as demonstrated by fluorinated silica nanoparticle capsules dispersed in a ceramic binder matrix [314]. When mechanical abrasion occurs, these particle capsules release their payload of fluorinated nanoparticles to the surface, instantaneously regenerating superamphiphobicity. This design enables the coating to maintain excellent repellency even after 500 Taber abrasion cycles under 250 g load, demonstrating exceptional durability against combined physical and chemical challenges including oil immersion and sand-water erosion.

For enhanced resilience in chemically harsh environments, composite reinforcement strategies offer robust solutions through multiple material combinations. The ST@PUa/C coating system (Fig. 21f) exemplifies this approach through a multi-component design: polyurea-silica composite particles provide mechanical reinforcement through high-hardness silica components, while graphite powder contributes photothermal stability [315]. This composite architecture maintains superhydrophobicity (WCA > 150°) after 360 h of immersion in extreme pH solutions (pH = 1 and 14) and salt corrosion (Fig. 21g), and withstands 25 icing–deicing cycles with minimal performance degradation (Fig. 21h). The incorporation of silica significantly enhances abrasion resistance, as evidenced by the coating maintaining a WCA above 143° after 50 cycles of sand impact tests, while the graphite ensures stable photothermal performance under prolonged environmental exposure.

Furthermore, another composite reinforcement strategy focuses on embedding stable, reductant photothermal components to prevent oxidation-induced performance degradation in outdoor environments. The fluorinated graphene-Ecoflex elastic coating demonstrates this approach, where modified graphene is partially embedded into an Ecoflex elastomer matrix [316]. This design maintains superhydrophobicity even under 300% strain due to the excellent resilience of Ecoflex, while the graphene provides outstanding electrothermal performance-melting a 2-mm-thick ice layer within 115 s at 20 V. More importantly, the embedded graphene exhibits superior oxidation resistance, ensuring long-term photothermal stability that is critical for outdoor applications.

Addressing thermal management challenges, overheat-proof protection represents another critical advancement with diverse implementation pathways [315]. The dual-shell microcapsule system n-Eicosane@Cu_2_O (Fig. 21i–m) incorporates phase-change materials (PCMs) for intelligent thermal regulation [317]. As detailed in Fig. 21i, the innovative dual-shell design features Cu_2_O as the inner layer and 3D Cu_2−*x*_S as the outer layer, achieving full-spectrum high absorption with a remarkable photothermal conversion efficiency of 96.1%. The morphological evolution from S1 to S4 (Fig. 21j–k3) demonstrates controlled growth of Cu_2−x_S nanocrystals, with S3 (Fig. 21l) showing optimal flower-like nanostructures. The crushed microcapsule (Fig. 21m) clearly reveals the dual-shell structure with Kirkendall voids. As shown in Fig. 21n, the optimized S3 microcapsules exhibit suitable phase transition temperatures (*T*_m_ ≈ 28 °C) and significantly reduced supercooling degrees (Δ*T*), enabling effective heat storage and release. The phase-change enthalpy remains consistent after 200 cycles, demonstrating excellent thermal cycling stability. When integrated into building materials (Fig. 21o), this system maintains indoor temperatures within a comfortable range despite external temperature fluctuations, showing both passive heating in winter conditions and cooling effects in summer scenarios, with the S3-containing composite (C4) maintaining temperatures around 32 °C in high-temperature environments.

Moreover, an innovative thermochromic approach provides active thermal regulation through reversible color changes [318]. The thermochromic phase transition heat storage coating (TCHSC) contains microcapsules that reversibly transition between black (high photothermal efficiency) and white (low efficiency) states based on ambient temperature. This intelligent system prevents midday overheating during non-icing periods while maintaining effective anti-icing performance in cold conditions. The discoloration mechanism involves the formation of unconjugated structures in thermochromic dyes, simultaneously storing heat during phase transition to modulate temperature dynamics. Critically, the transition temperature can be tuned by adjusting the dye’s melting point, enabling customized thermal management for specific climatic conditions.

Collectively, these advanced reinforcement strategies—spanning hierarchical structural armor, covalent/strong anchoring, composite reinforcement, autonomous self-healing, and thermal overheat protection—form a comprehensive multi-level defense system that fundamentally redefines durability in photothermal superhydrophobic textiles. The integration of passive structural designs (hierarchical and covalent anchoring) with active functional materials (self-healing polymers, composite matrices, and intelligent thermal regulators) creates a synergistic ecosystem where each component addresses specific failure modes while collectively enhancing overall resilience. This evolutionary progression from mere damage resistance to adaptive functionality and intelligent self-regulation represents a paradigm shift in material design philosophy. By enabling textiles to actively maintain their performance under real-world stresses, whether through molecular-level chain migration, capsule-based repair mechanisms, oxidation-resistant composites, or smart thermal modulation, these strategies collectively pave the way for the development of next-generation photothermal superhydrophobic materials that balance technological sophistication with practical viability for sustainable applications.

### Toxicity and Environmental Impact

The widespread adoption of photothermal superhydrophobic textiles hinges on their safety for human use and environmental sustainability, as many lab-scale materials rely on components that raise concerns about biocompatibility and ecological footprint. A frequently overlooked environmental hazard in textile development is the toxic solvents used in solution-based fabrication methods—including halogenated solvents (e.g., chloroform, dichloromethane) and aromatic hydrocarbons (e.g., toluene)—which are often volatile, persistent, and toxic to both humans and ecosystems [319]. These solvents are typically used for dissolving low-surface-energy polymers or dispersing nanoparticles, yet their release during production (via evaporation) or disposal (via wastewater) is rarely fully accounted for in lab-scale studies, contributing to air and water pollution [320].

**Fig. 22:**
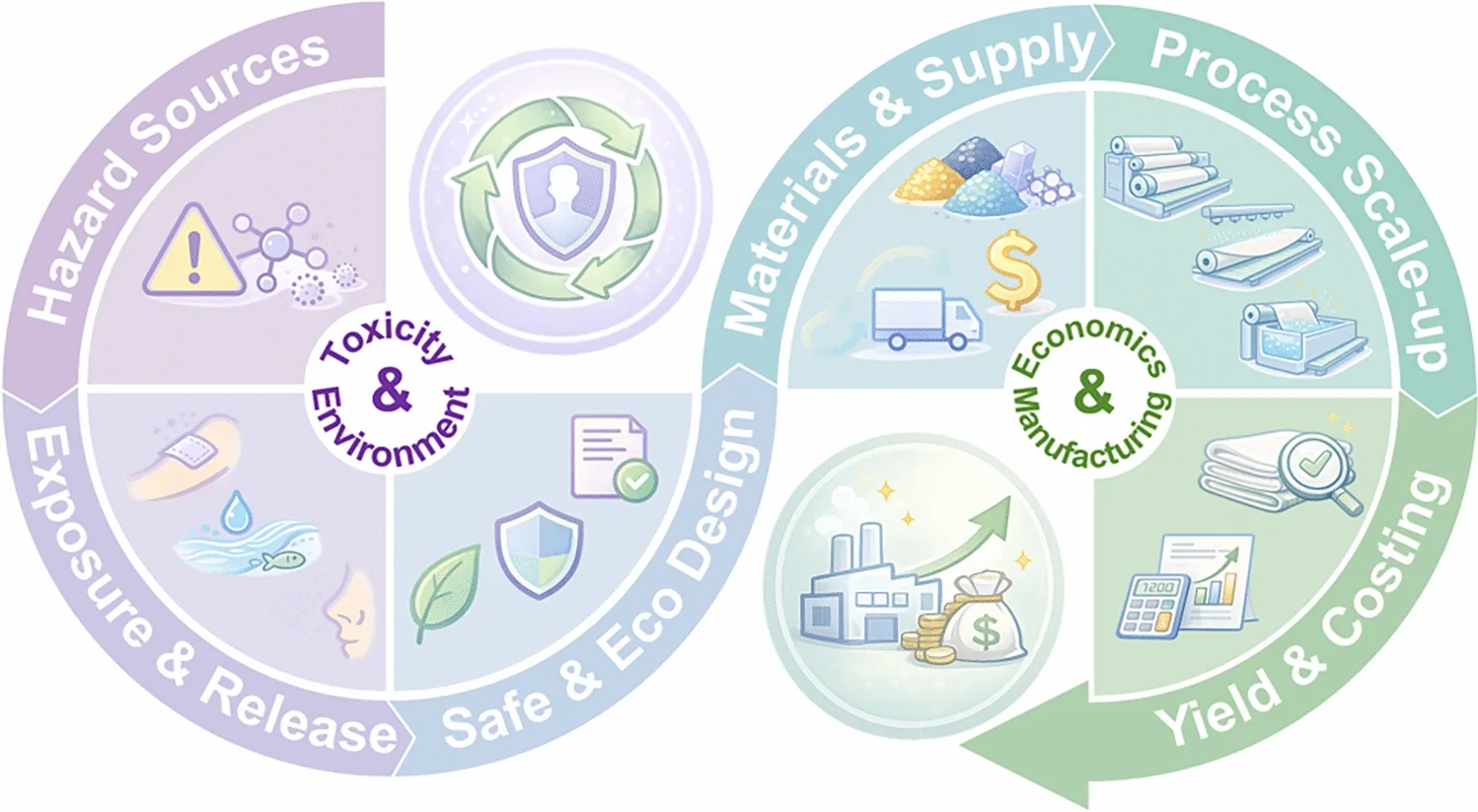
Dual-loop “from lab-to-life” roadmap for photothermal superhydrophobic textiles. The toxicity and environment loop addresses risk mitigation by systematically covering hazard identification, exposure and release assessment, and safe and eco-friendly design, including regulatory compliance, fluorine-free material substitution, and encapsulation or anchoring strategies. In parallel, the economics and manufacturing loop supports commercialization through coordinated materials and supply selection, process scale-up based on continuous textile production lines, and yield-driven costing combined with techno-economic analysis to evaluate feasibility

Figure 22 (left section) outlines a dual-loop “from lab to life” roadmap that addresses these challenges, with the toxicity and environment loop systematically closing risks through three core stages: hazard identification, exposure/release assessment, and safe and eco-design. Hazard identification focuses on pinpointing potential toxic components in photothermal superhydrophobic textiles, such as toxic small molecules (e.g., residual monomers from polymerization) and low-surface-energy organics (e.g., fluorinated compounds like PTFE or perfluorooctanoic acid, PFOA) [321]. These substances are of particular concern due to their persistence in ecosystems and potential to bioaccumulate, as highlighted by studies linking fluorinated compounds to endocrine disruption and developmental toxicity in aquatic organisms [320, 322, 323]. Exposure/release assessment then evaluates how these hazards might enter the environment or contact humans. Key pathways include direct contact (skin absorption of nanoparticles or volatile organic compounds), waterborne release (leaching of fluorinated coatings into wastewater during washing), and aerosol propagation (dust generation of loose nanoparticles during textile manufacturing or disposal) [324, 325]. For example, MXene nanoparticles—commonly used for photothermal activity—have been shown to release toxic metal ions (e.g., titanium, aluminum) when degraded by UV light, posing risks to both human health and aquatic ecosystems [326].

Another important but underexamined release pathway is microfiber and microplastic generation during washing, abrasion, wearing, and disposal. This issue is particularly relevant for photothermal superhydrophobic textiles, which often combine synthetic or semi-synthetic substrates (PET, PP, PLA, nylon, or coated cotton) with polymer binders and functional nano-/microfillers. Household washing is a major route for textile microplastic release, and release behavior depends not only on fiber type (synthetic vs. natural) but also on yarn construction, fabric structure, finishing treatments, and washing conditions [327–329].

For photothermal superhydrophobic textiles, the concern extends beyond ordinary microfiber shedding. Released fragments may become “functionalized textile microplastics” carrying hydrophobic coatings, photothermal fillers, metal nanoparticles, or residual organic modifiers [330]. These fragments pose combined risks of microplastic pollution, nanoparticle release, and additive leaching. Therefore, even when natural or biodegradable substrates (e.g., cotton, PLA) are used, the environmental profile should be evaluated at the whole-composite level rather than inferred from the substrate alone, especially since separating the coating from the fiber matrix remains difficult after repeated use or at end-of-life.

Future studies should incorporate microfiber-release and end-of-life assessments into environmental evaluations. Quantitative testing should examine wash-induced fiber release, abrasion debris, coating detachment, and changes in particle composition after aging. At the design stage, circular principles should be considered: mixed-fiber fabrics, multilayer structures, and strongly adhered finishes can complicate sorting, fiber recovery, and chemical recycling [331]. Sustainable photothermal superhydrophobic textiles should minimize microfiber generation, immobilize functional fillers, simplify material composition, and improve compatibility with recycling or safe disposal pathways, in addition to reducing toxic solvents and fluorinated modifiers.

To mitigate these risks, the safe and eco-design stage prioritizes strategies aligned with global environmental regulations (e.g., the EU’s REACH directive, which restricts PFOA) and sustainability goals. A primary approach is fluorine-free substitution, replacing persistent fluorinated compounds with biodegradable alternatives like PDMS or natural waxes [305, 332]. Another strategy is encapsulation/anchoring, which immobilizes nanoparticles (e.g., Ag NPs, MXene) within crosslinked polymer matrices to prevent leaching [93]. For instance, the CFC/MnO_2_/PLL system (Fig. 15) uses poly-L-lysine—a natural, biocompatible polymer—to anchor MnO_2_ nanoparticles, eliminating the need for fluorinated coatings while maintaining antibacterial activity [291]. Recent research has also shifted toward green materials and biodegradable substrates to reduce environmental impact. The FAMP textile (Fig. 13) uses PLA—a biodegradable polymer derived from corn starch—as its base, replacing petroleum-based fibers like PET [244]. Life-cycle assessments (LCAs) further confirm that PLA-based textiles decompose within 1–2 years in composting conditions, compared to PET’s over 400 years of persistence [333].

By integrating hazard identification, exposure assessment, and eco-friendly design, photothermal superhydrophobic textiles are transitioning from lab-scale innovations to sustainable solutions that comply with global environmental standards. This holistic approach not only ensures safety for human use but also minimizes ecological harm, paving the way for widespread commercial adoption.

### Economics and Manufacturing Scalability

For photothermal superhydrophobic textiles to compete with conventional materials and achieve mass-market adoption, they must be cost-effective to produce at scale—a critical gap between lab-scale innovation and real-world deployment. Figure 22 (right section) outlines the economics and manufacturing loop of the dual-loop “from lab to life” roadmap, which enables commercialization through three interconnected stages: materials and supply selection, process scale-up, and yield-driven costing/techno-economic analysis (TEA) feasibility.

The first stage, materials and supply selection, focuses on balancing performance with cost and supply chain viability. Lab-scale studies often prioritize high-performance but expensive materials (e.g., specialty MXene flakes, rare metal nanoparticles), which are impractical for mass production due to limited availability and volatile pricing [334, 335]. Instead, commercialization requires shifting to abundant, low-cost raw materials—such as cotton, PET, or PLA (biodegradable) as substrates, and PDMS or natural waxes as low-surface-energy coatings—while ensuring consistent supply chains to avoid production bottlenecks [336]. For example, replacing scarce MXene with CNTs or graphene oxide (GO) (both produced at industrial scales) can reduce raw material costs by up to 60% without significant loss of photothermal activity [337, 338].

The second stage, process scale-up, addresses the transition from batch laboratory techniques (e.g., dip-coating, spray-coating) to continuous textile manufacturing lines (e.g., roll-to-roll coating, pad-dry-cure processes) that are standard in the textile industry. Batch methods typically have low throughput (10–100 m^2^ day^−1^) and high labor costs, whereas continuous lines can produce up to 10,000 m^2^ day^−1^ with minimal human intervention [339, 340]. A key challenge here is adapting laboratory protocols to existing industrial equipment: for instance, the PDMS/Fe_3_O_4_/PPy@PET fabric (Fig. 12) was initially developed via batch chemical oxidation polymerization, but researchers later optimized it for roll-to-roll processing by simplifying the coating sequence and using water-based dispersions instead of toxic solvents [25]. This adaptation reduced production time by 70% and increased throughput by an order of magnitude.

The final stage, yield-driven costing/TEA feasibility, involves optimizing production yields and calculating profitability to ensure commercial viability. Lab-scale processes often have low yields (50%–70%) due to inconsistent coating uniformity or nanoparticle agglomeration, but industrial-scale optimization can boost yields to over 90% [341]. For example, the AgCFP textile (Fig. 18) initially had a yield of 65% in laboratory trials, but by automating the silver nanoparticle deposition step and using inline quality control (e.g., real-time WCA measurement), yields improved to 92%, reducing per-unit costs by 35% [222]. TEA further evaluates factors like capital expenditure (CAPEX), operational expenditure (OPEX), and break-even points: a recent TEA of fluorine-free superhydrophobic textiles found that a production line with 10,000 m^2^ day^−1^ capacity could achieve break even in 2.5 years, assuming a 20% profit margin and a market price of $50 m^−2^ [342, 343].

By systematically addressing materials selection, process scalability, and economic feasibility, the economics and manufacturing loop bridges the gap between laboratory innovation and commercial success. This holistic approach ensures that photothermal superhydrophobic textiles are not only technically advanced but also economically viable for widespread use.

In addition, interfacial adhesion should be treated as a manufacturing quality control parameter during scale-up. For continuous processing, coating uniformity and adhesion retention should be statistically evaluated along the fabric length and width, because local particle shedding or binder failure can simultaneously reduce superhydrophobicity, photothermal stability, and washing durability.

### Critical Gaps in Standardized Performance Evaluation and Sustainable Design

Despite rapid progress, most studies on photothermal superhydrophobic textiles emphasize successful demonstrations rather than systematic identification of limitations. Evaluation methods, reporting formats, and sustainability considerations remain highly inconsistent, hindering rigorous comparison and practical translation.

A primary issue is insufficient reporting of photothermal parameters. Many studies report only maximum surface temperature or heating curves, while quantitative photothermal conversion efficiency (η), solar absorptivity, and heat-loss pathways are often omitted [235–240]. This omission is particularly problematic for porous textiles, where measured temperature is strongly affected by fabric thickness, pore structure, coating loading, and testing conditions. Future studies should report irradiation intensity, illuminated area, solar absorptivity, *η*, cooling behavior, and post-durability retention, enabling true distinction between efficient materials and those yielding high surface temperature under favorable conditions.

The second gap is the lack of standardized durability evaluation. Mechanical abrasion, washing, UV aging, thermal cycling, and chemical exposure are relevant for real textile service, but current protocols vary widely [119, 126, 138, 169, 201–207]. For instance, abrasion tests differ in abrasive paper type, load, and cycle number; washing tests may involve laundering cycles, ultrasonic treatment, or simple rinsing. Such inconsistency makes it difficult to judge true durability. Future work should provide post-treatment values of WCA, SA, CAH, *T*_max_, *η*, air permeability, and mechanical strength after standardized aging.

Dynamic wetting indicators are also insufficiently reported. While WCA is commonly used, it cannot fully describe liquid repellency. For self-cleaning, anti-icing, and wearable protection, SA, CAH, roll-off behavior, and droplet bouncing are often more relevant [196–205]. A textile with WCA > 150° may still exhibit high droplet adhesion due to contact line pinning. Therefore, dynamic parameters should be reported under defined droplet volume, inclination, temperature, and humidity.

Sustainable design remains another challenge. Many systems still rely on fluorinated silanes, PDMS, metal nanoparticles, or organic-solvent-assisted coating processes [14, 119, 129, 139, 184], raising concerns about environmental persistence, microplastic generation, and end-of-life treatment. The sustainability of the final composite—not just the original substrate—must be reassessed once natural fibers are coated with synthetic polymers or functional fillers.

The failure of superhydrophobic coatings on textiles further highlights interfacial design. Coatings relying on physical adsorption or simple spray deposition are vulnerable to delamination and particle shedding [129, 311–313]. Stronger anchoring strategies—covalent bonding, hydrogen bonding, polymer crosslinking, or bioinspired adhesion—are essential for long-term stability [311–313], but should be evaluated alongside breathability, softness, and recyclability.

Overall, future research should shift from performance demonstration to standardized, durability-oriented, and sustainability-aware evaluation. Reports should disclose testing conditions, report static and dynamic wetting parameters, quantify η, evaluate performance retention after realistic aging, and assess environmental implications of coatings, solvents, fillers, and disposal. Such systematic reporting is essential for advancing toward reliable, comparable, and scalable textile technologies.

## Concluding Remarks, Challenges, and Outlook

### Concluding Remarks

This review provides a unified framework that bridges material design, performance evaluation, and real-world deployment of photothermal superhydrophobic textiles—an integrative perspective largely absent from prior literature. Its core academic value lies in three contributions. First, by systematically decoupling the mutually reinforcing synergy between superhydrophobicity and photothermal conversion (Fig. 5), this work resolves a longstanding conceptual gap: how water repellency preserves photothermal efficiency, and how photothermal heating in turn stabilizes the non-wetting state. This mechanistic insight offers a foundational design principle for future multifunctional textiles. Second, the review introduces a holistic “lab-to-life” roadmap (Fig. 22) that explicitly connects material durability, environmental safety, and economic scalability—dimensions typically treated in isolation. Beyond simply cataloging performance metrics, we adopt a critical lens to evaluate the reproducibility, reliability, and practical validity of commonly reported characterization methods, urging the field to move toward standardized testing protocols. This framework provides actionable guidance for transitioning laboratory innovations into practical products. Third, by critically assessing solvent toxicity and advocating for green synthesis, this work advances a sustainability-oriented paradigm that aligns material development with global environmental regulations. Importantly, we extend this environmental analysis to the emerging concern of microplastic pollution from textile degradation and highlight the need for biodegradable or recyclable material choices as an integral part of responsible design. Together, these contributions establish a comprehensive reference that not only consolidates current knowledge but also defines clear pathways for future research—from fundamental mechanism studies to scalable, eco-friendly manufacturing—thereby accelerating the field toward technological maturity and real-world impact.

### Challenges and Current limitations

**Fig. 23:**
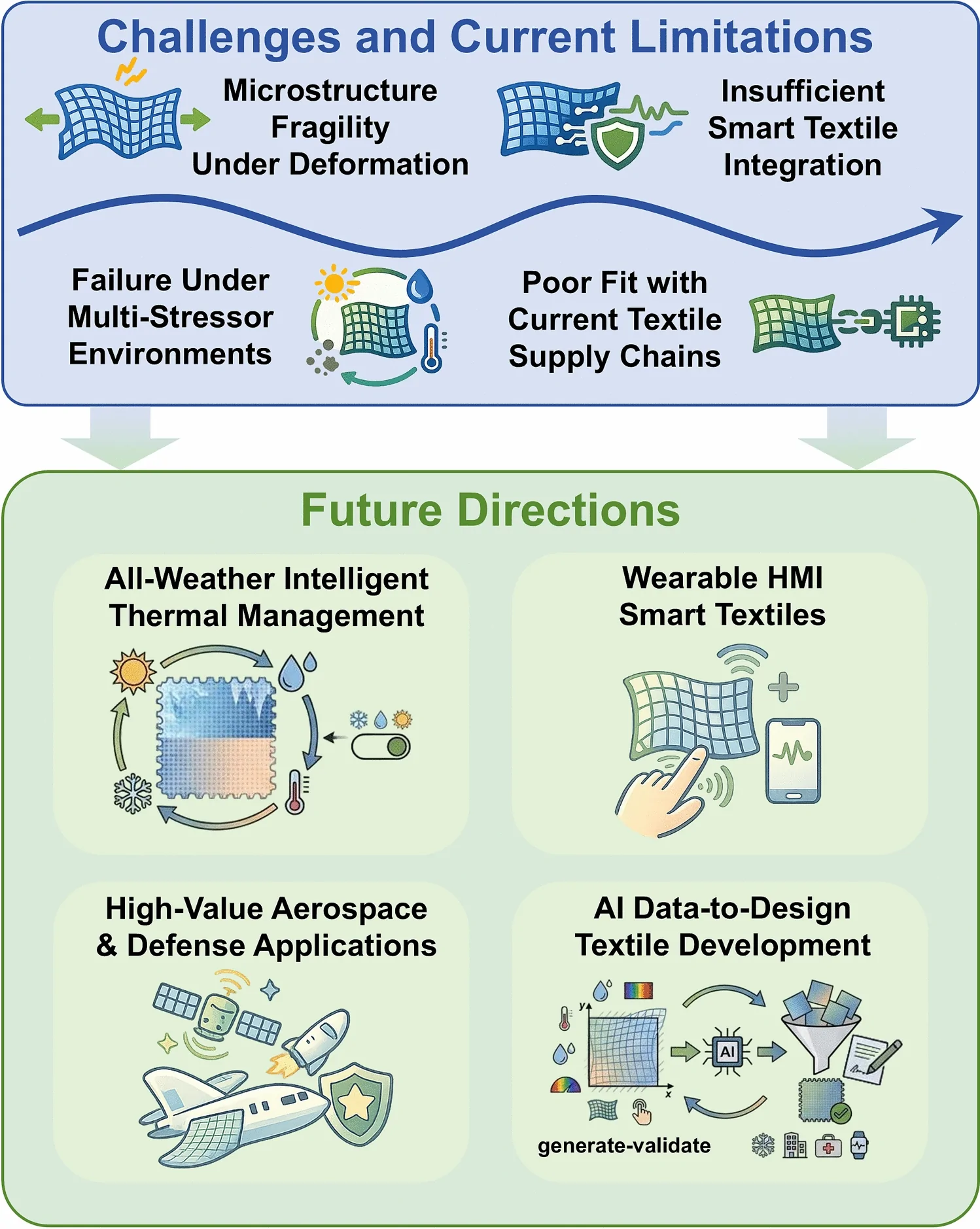
Schematic summary of key challenges and future directions for photothermal superhydrophobic textiles toward practical deployment. The upper panel highlights four major challenges, and the lower panel outlines corresponding future directions

Despite significant progress in photothermal superhydrophobic textiles, critical barriers remain to their widespread practical deployment, as synthesized in the upper panel of Fig. 23, which identifies four core challenges: microstructure fragility under deformation, failure under multi-stressor environments, insufficient smart textile integration, and poor fit with current textile supply chains. These challenges are interconnected, often amplifying each other to hinder real-world adoption, even for materials with exceptional lab-scale performance.

The first and most pervasive challenge is microstructure fragility under deformation, where the hierarchical nano-/microstructures critical for superhydrophobicity and photothermal activity degrade under mechanical stress (e.g., stretching, folding, or abrasion). Lab-scale materials like PMCNF (MnO_2_@CuCo_2_O_4_/Ni foam) exhibit impressive durability under controlled sandpaper abrasion [311], but real-world use, such as repeated bending of wearable textiles or stretching of outdoor gear, induces localized structural damage that disrupts the Cassie-Baxter state. For example, MXene-coated cotton fabrics lose 30% of their photothermal conversion efficiency after 50 folding cycles, as the brittle MXene flakes crack and detach from the flexible fiber substrate [139]. This fragility stems from a mismatch between rigid functional coatings and soft textile substrates, a gap rarely addressed in laboratory studies that prioritize static performance over dynamic mechanical resilience.

Compounding this is the second challenge: failure under multi-stressor environments, where textiles are exposed to combined hazards (e.g., UV irradiation, saltwater and mechanical abrasion) rather than single stressors. Lab-scale durability tests typically evaluate one stressor at a time (e.g., only washing or only UV exposure), but real-world applications, such as marine anti-icing coatings or desert outdoor apparel, subject materials to simultaneous UV degradation, chemical corrosion, and physical wear. A recent study found that superhydrophobic photothermal textiles designed for offshore wind turbines lost 60% of their superhydrophobicity after 30 days of exposure to salt spray and UV radiation, as the salt crystals eroded the hierarchical roughness and UV light degraded the low-surface-energy PDMS coating [126, 220, 344]. This multi-stressor failure is often overlooked in controlled laboratory settings, leading to overoptimistic performance claims that do not translate to field use.

The third challenge is insufficient smart textile integration, where photothermal superhydrophobic textiles lack compatibility with emerging smart functionalities (e.g., sensors, energy harvesting, or responsive actuation). While lab-scale materials often demonstrate standalone performance (e.g., self-cleaning and photothermal deicing), integrating them with electronic components—such as flexible temperature sensors for wearable heating, requires balancing functional layers without compromising either superhydrophobicity or electronic conductivity. For instance, coating a conductive textile with a superhydrophobic layer can block electrical signals by creating an insulating barrier, while embedding sensors into the textile can disrupt the hierarchical roughness needed for water repellency [345–347]. This integration gap limits the textiles’ utility in smart wearables or internet of things (IoT)-enabled infrastructure, where multi-functionality is non-negotiable.

Finally, poor fit with current textile supply chains hinders industrial scalability. Lab-scale fabrication relies on custom batch processes (e.g., dip-coating with toxic solvents) that are incompatible with continuous industrial lines (e.g., roll-to-roll coating or pad-dry-cure systems). For example, the in situ diazonium polymerization used to prepare CF_3_Ph-Cu_x_O@CF fabrics requires precise temperature control and hazardous solvents, making it impossible to adapt to standard textile manufacturing equipment [313]. Additionally, many functional materials (e.g., specialty MXene flakes) are not produced at industrial scales, leading to supply chain bottlenecks and volatile pricing that deter manufacturers from adopting these innovations [348–350].

Beyond these technical barriers, environmental concerns related to microfiber and microplastic release during washing and end-of-life disposal represent an increasingly critical yet underexplored challenge. As discussed in Sect. 9.3, functional coatings and synthetic substrates in photothermal superhydrophobic textiles may generate “functionalized textile microplastics” carrying hydrophobic polymers, photothermal fillers, or metal nanoparticles, posing combined risks of microplastic pollution and additive leaching [330, 331].

Together, these challenges highlight that while photothermal superhydrophobic textiles hold great promise, their transition from lab to life requires addressing not just material performance, but also dynamic resilience, multi-stressor durability, smart integration, and supply chain compatibility—gaps that must be prioritized in future research.

### Outlook and Future Directions

While the challenges outlined in Sect. 10.2 underscore the gaps between laboratory innovation and real-world deployment, they also point to transformative opportunities for photothermal superhydrophobic textiles—opportunities that align with the four future directions highlighted in the lower panel of Fig. 23. These directions not only address existing limitations but also unlock high-value applications that could redefine the textile industry, making them critical priorities for researchers and industry alike.

The first future direction is all-weather intelligent thermal management, which aims to develop textiles that dynamically adapt to extreme environmental conditions (e.g., cold, heat, humidity) by integrating photothermal superhydrophobicity with responsive thermal regulation. Unlike current static materials that only heat or repel water, all-weather textiles would switch between modes: absorbing solar energy for heating in cold environments, reflecting sunlight for cooling in hot climates, and repelling water/ice year-round. For example, a textile coated with thermochromic photothermal nanoparticles could adjust its light absorption based on temperature—absorbing 90% of sunlight below 10 °C for heating and reflecting 80% above 30 °C for cooling, while maintaining superhydrophobicity to prevent rain or sweat accumulation [139, 271, 351]. This direction addresses the multi-stressor limitation by designing materials that thrive in variable conditions, with potential applications in outdoor apparel, building insulation, and agricultural covers.

The second direction is wearable human–machine interface (HMI) Smart Textiles, which merges photothermal superhydrophobicity with flexible electronics to create interactive textiles for healthcare, fitness, and augmented reality. Current wearable devices often suffer from water damage or poor comfort, but photothermal superhydrophobic textiles could solve both: the superhydrophobic layer protects electronic components from sweat or rain, while the photothermal effect provides localized heating for therapeutic purposes (e.g., muscle relaxation). For instance, a smart textile sleeve could integrate pressure sensors to track movement, a photothermal layer to soothe sore muscles, and a superhydrophobic coating to repel sweat—all while remaining breathable and washable [352–354]. This direction resolves the smart integration gap by designing functional layers that complement rather than compete with electronics, opening new avenues for personalized healthcare and human–machine interaction.

The third direction is high-value aerospace and defense applications, where photothermal superhydrophobic textiles can address unmet needs in extreme environments (e.g., aircraft anti-icing, space suit thermal regulation). Aerospace surfaces are prone to ice accumulation and UV degradation, but photothermal superhydrophobic textiles could provide passive deicing (via solar heat) and self-cleaning (via water repellency) without heavy mechanical systems. For example, a textile coating for aircraft wings could melt ice within 5 min of solar exposure while repelling rain to prevent re-icing, reducing fuel consumption by up to 15% compared to traditional deicing methods [355–357]. This direction leverages the multi-stressor resilience challenge by designing materials for the harshest conditions, with potential to disrupt aerospace and defense industries.

The fourth direction is AI-assisted data-to-design development, which aims to accelerate the rational design of photothermal superhydrophobic textiles. Unlike conventional trial-and-error approaches, AI can extract quantitative relationships from high-dimensional variables—including fiber type, photothermal filler composition and loading, coating thickness, surface roughness, pore structure, WCA, SA, photothermal conversion efficiency, air permeability, mechanical durability, cost, and environmental safety—making it particularly suited for performance prediction, formulation screening, and multi-objective optimization. A first practical role lies in standardizing characterization: machine learning models have achieved accurate hydrophobic contact angle measurements from large droplet-profile datasets [358], which is highly relevant because the fibrous, rough nature of fabrics often makes wetting measurements less reproducible than on flat substrates, and AI-assisted image analysis could reduce operator bias and improve comparability across laboratories. Building on this, a second role is predicting coating stability and durability—machine learning has been used to analyze the stability of eco-friendly superhydrophobic graphene-based coatings [359], and similar models for photothermal superhydrophobic textiles could be trained using descriptors such as coating chemistry, roughness scale, abrasion load, washing cycles, and UV exposure to identify combinations likely to maintain performance under long-term stress. In addition to stability prediction, a third role is optimizing functional textile processing: machine learning-assisted development of PPy-grafted yarns for e-textiles has shown that carefully selected experiments can identify optimal conditions and reduce experimental burden [360], a strategy directly applicable to photothermal superhydrophobic textiles where in situ polymerization, spray-coating, and dip-coating involve many coupled parameters that AI-guided design could optimize. Beyond process optimization, a fourth role is photothermal material screening—machine learning-guided exploration of carbon-based photothermal materials for solar evaporation—has demonstrated accelerated identification of high-performance light-to-heat conversion materials [361], and similar strategies could screen carbon materials, MXenes, conductive polymers, and plasmonic nanoparticles based on absorptance, efficiency, stability, and coating compatibility. Finally, generative models may further expand the design space: recent studies on GANs and diffusion models show that generative AI can propose candidate structures beyond simple database screening [362], and for photothermal superhydrophobic textiles, such models could suggest hierarchical surface architectures or multilayer structures balancing light trapping, air retention, and breathability. However, AI should be regarded as an auxiliary design tool rather than a replacement for experiments—because datasets in this field remain small and non-standardized, AI predictions must be validated by standardized wettability measurements, photothermal tests, and durability evaluation, and future progress requires open databases integrating material composition, fabrication parameters, wetting behavior, photothermal metrics, and durability results to allow AI to become a practical tool for accelerating textile development [363, 364].

As we look to the future, photothermal superhydrophobic textiles stand at the cusp of revolutionizing industries from healthcare to aerospace. While the path to deployment is fraught with challenges, these challenges are not barriers—they are invitations to innovate, to push the boundaries of material science, and to create textiles that are not just functional, but intelligent, sustainable, and life-changing. Imagine a world where your jacket heats you in the cold, cools you in the heat, repels rain, and monitors your health; where aircraft wings deice themselves with sunlight; where agricultural covers boost crop yields by regulating temperature and repelling pests. This is not a distant dream—it is a future within reach, waiting for researchers to seize the opportunities, overcome the challenges, and turn possibility into reality. The horizon for photothermal superhydrophobic textiles is bright, and the journey ahead promises to be as rewarding as the destination.

## References

[1] J. Dong, B. Zhou, G. Han, J. Gao, X. Liu et al., A multi-mode thermoregulating fabric with integrated passive/active temperature control capabilities. Nano Res. **18**(10), 94907680 (2025). 10.26599/NR.2025.94907680

[2] G.H. Yang, J. Lin, H. Cheung, G. Rui, Y. Zhao et al., Single layer silk and cotton woven fabrics for acoustic emission and active sound suppression. Adv. Mater. **36**(28), 2313328 (2024). 10.1002/adma.202313328 38561634

[3] G.-X. Li, T. Dong, L. Zhu, T. Cui, S. Chen, Microfluidic-blow-spinning fabricated sandwiched structural fabrics for all-season personal thermal management. Chem. Eng. J. **453**, 139763 (2023). 10.1016/j.cej.2022.139763

[4] P. Alarcon-Espejo, R. Sarabia-Riquelme, G.M. Matrone, M. Shahi, S. Mahmoudi et al., High-hole-mobility fiber organic electrochemical transistors for next-generation adaptive neuromorphic bio-hybrid technologies. Adv. Mater. **36**(11), 2305371 (2024). 10.1002/adma.202305371 37824715

[5] Q. Pan, Y. Bai, X. Xu, X. Fang, F. Li, Active waste heat recovery with dual-functional CoS_2_/MoS_2_ fabric for efficient photo/electro-thermal evaporation. Chem. Eng. J. **529**, 173134 (2026). 10.1016/j.cej.2026.173134

[6] L. Liu, Z. Ma, M. Zhu, L. Liu, J. Dai et al., Superhydrophobic self-extinguishing cotton fabrics for electromagnetic interference shielding and human motion detection. J. Mater. Sci. Technol. **132**, 59–68 (2023). 10.1016/j.jmst.2022.05.036

[7] M. Xu, J. Li, J. Ren, J. Wang, L. Xu et al., Superhydrophobic and recyclable passive daytime radiative cooling fabric prepared via electrospinning. Chem. Eng. J. **509**, 161274 (2025). 10.1016/j.cej.2025.161274

[8] Z. Zhang, Y. Meng, X. Fang, Q. Wang, X. Wang et al., Robust, flexible, and superhydrophobic fabrics for high-efficiency and ultrawide-band microwave absorption. Engineering **41**, 161–171 (2024). 10.1016/j.eng.2024.03.009

[9] K. Chen, Y. Li, G. Yang, S. Hu, Z. Shi et al., Fabric-based TENG woven with bio-fabricated superhydrophobic bacterial cellulose fiber for energy harvesting and motion detection. Adv. Funct. Mater. **33**(45), 2304809 (2023). 10.1002/adfm.202304809

[10] X. Feng, X. Guo, K. Chen, S. Qian, J. Sun et al., Breathable, self-healable, durable superhydrophobic and UV-blocking cotton fabrics. Chem. Eng. J. **492**, 152420 (2024). 10.1016/j.cej.2024.152420

[11] J. Liu, J. Zhao, Z. Lu, G. Tian, H. Zhang et al., Durable and superhydrophobic cellulose-containing fabrics for self-cleaning and oil-water separation. Chem. Eng. J. **525**, 170129 (2025). 10.1016/j.cej.2025.170129

[12] T. Ali, N. Ahmad, W. Ahmad, H.-M. Xiao, S.M. Jawad et al., Engineering multifunctional fire proof superhydrophobic cotton fabric via sequential coating of flower-like PPPA/BMOF and PDMS. Chem. Eng. J. **526**, 171467 (2025). 10.1016/j.cej.2025.171467

[13] Y. Meng, Z. Zhang, X. Wang, X. Hou, T. Wang et al., Flexible, superhydrophobic, and self-cleaning rGO/LDH/PPy-modified fabric for full X-band electromagnetic wave absorption. Compos. Part B Eng. **282**, 111572 (2024). 10.1016/j.compositesb.2024.111572

[14] X. Sha, L. Chen, Y. Jia, H. Zhao, S. Zuo et al., Preparation and properties of sustainable superhydrophobic cotton fabrics modified with lignin nanoparticles, tannic acid and methyltrimethoxysilane. Chem. Eng. J. **499**, 155797 (2024). 10.1016/j.cej.2024.155797

[15] S. Yang, H. Li, S. Liu, S. Wang, H. Li et al., Wodyetia bifurcate structured carbon fabrics with durable superhydrophobicity for high-efficiency oil-water separation. J. Hazard. Mater. **439**, 129688 (2022). 10.1016/j.jhazmat.2022.129688 36104914

[16] L. Dai, X. Fan, Reversible simultaneous switching between bacteria-killing/superhydrophobicity and bacteria-releasing/superhydrophilicity cotton fabric for potential oil/water separation application. J. Clean. Prod. **442**, 141004 (2024). 10.1016/j.jclepro.2024.141004

[17] J. Kang, F. Xiang, X. He, Z. Li, Preparation of robust silicone superhydrophobic and antibacterial textiles using the Pickering emulsion method. Carbohydr. Polym. **323**, 121419 (2024). 10.1016/j.carbpol.2023.121419 37940251

[18] T. Ali, N. Ahmad, M.I. Nabeel, H.-M. Xiao, D. Hussain, Facile and scalable growth of bimetallic 3D Ag/MIL125-NH_2_ on cotton fabric for multifaceted anti-wetting and self-healing characteristics. Carbon **228**, 119395 (2024). 10.1016/j.carbon.2024.119395

[19] S.K. Park, S.Y. Lee, S.B. Kim, I.H. Kim, D.U. Lee et al., Dual-action antibacterial nanoblades for rapid inactivation of bioaerosols in personal protective equipment. Adv. Funct. Mater. **35**(30), 2421728 (2025). 10.1002/adfm.202421728

[20] C.-B. Li, F. Wang, R.-Y. Sun, W.-C. Nie, F. Song et al., A multifunctional coating towards superhydrophobicity, flame retardancy and antibacterial performances. Chem. Eng. J. **450**, 138031 (2022). 10.1016/j.cej.2022.138031

[21] R.-Y. Sun, F. Wang, Y. Tan, J.-L. Li, Z.-S. Jiang et al., Breathable nanorod-embedded hierarchical photothermal coatings with anti-soiling and safe thermal regulation for efficient anti-icing and de-icing. Small **21**(35), 2506234 (2025). 10.1002/smll.202506234 40643018

[22] Z. Chen, X. Xu, W. Li, L. Feng, K. Wang et al., Crisscross metal–organic frameworks superhydrophobic structure for high efficient emulsion separation and anti-icing. Chem. Eng. J. **509**, 161228 (2025). 10.1016/j.cej.2025.161228

[23] J.-L. Li, F. Wang, C.-B. Li, R.-Y. Sun, M.-L. Guo et al., A top-down and in situ strategy for intrinsic acicular nanostructure enhanced ultradurable superhydrophobic and anti-icing aramid fabric. Chem. Eng. J. **484**, 149569 (2024). 10.1016/j.cej.2024.149569

[24] S. Yang, L. Chen, S. Wang, S. Liu, Q. Xu et al., Honeycomb-like cobalt hydroxide nanosheets induced basalt fiber fabrics with robust and durable superhydrophobicity for anti-icing and oil-water separation. J. Hazard. Mater. **429**, 128284 (2022). 10.1016/j.jhazmat.2022.128284 35066220

[25] G. Li, X. Wang, D. Zhang, H. Fu, X. Qian et al., Superhydrophobic magnetically responsive fabric heater with triple thermal conversion for all-weather anti-icing/deicing. Chem. Eng. J. **500**, 156674 (2024). 10.1016/j.cej.2024.156674

[26] S. Yang, J. Liu, M.J. Hoque, A. Huang, Y. Chen et al., A critical perspective on photothermal de‐icing. Adv. Mater. **37**(7), 2415237 (2025). 10.1002/adma.202415237 PMC11837899 39711482

[27] F. Chu, Z. Hu, Y. Feng, N. Lai, X. Wu et al., Advanced anti‐icing strategies and technologies by macrostructured photothermal storage superhydrophobic surfaces. Adv. Mater. **36**(31), 2402897 (2024). 10.1002/adma.202402897 38801015

[28] H. Chu, Q. Xu, Z. Liu, N. Xu, H. Zhang, Eco-friendly photothermal superhydrophobic coatings: recently advances in icephobic mechanisms, efficient photothermal conversion, and sustainable anti/de-icing technologies. Adv. Colloid Interface Sci. **343**, 103565 (2025). 10.1016/j.cis.2025.103565 40499411

[29] K. Zhang, J. Huang, S. Lu, Y. Hu, W. Pan et al., Multi-scale biomimetic strategy: robust woven wires with photo-thermal de-icing and spontaneous de-wetting. Adv. Funct. Mater. **35**(47), 2423043 (2025). 10.1002/adfm.202423043

[30] H. Xie, W.-H. Xu, Y. Du, J. Gong, R. Niu et al., Cost-effective fabrication of micro-nanostructured superhydrophobic polyethylene/Graphene foam with self-floating, optical trapping, acid-/alkali resistance for efficient photothermal deicing and interfacial evaporation. Small **18**(17), 2200175 (2022). 10.1002/smll.202200175 35307967

[31] G. Graeber, K. Regulagadda, P. Hodel, C. Küttel, D. Landolf et al., Leidenfrost droplet trampolining. Nat. Commun. **12**(1), 1727 (2021). 10.1038/s41467-021-21981-z PMC7979863 33741968

[32] H. Gu, B. Ji, J. Zhang, Y. Zhu, R. Tao et al., Leidenfrost effect on engineered surfaces. Adv. Funct. Mater. **36**(6), 2423686 (2026). 10.1002/adfm.202423686

[33] H.-B. Yuan, L. Wei, J. Wang, M. Zhao, G. Chen et al., Robust and breathable electronic textiles with anti-icing and photothermal de-icing properties modified by ZIF-8/polypyrrole. Chem. Eng. J. **509**, 161569 (2025). 10.1016/j.cej.2025.161569

[34] F. Meng, W. Hao, S. Yu, R. Feng, Y. Liu et al., Vapor-enabled propulsion for plasmonic photothermal motor at the liquid/air interface. J. Am. Chem. Soc. **139**(36), 12362–12365 (2017). 10.1021/jacs.7b06036 28837327

[35] Y. Xu, Y. Tang, L. Liu, Y. Zhang, C. Zhu et al., Water lily-inspired CNT@cotton towel solar evaporator with Marangoni effect and optimal water supply for achieving zero liquid discharge desalination and irrigation. Chem. Eng. J. **497**, 154827 (2024). 10.1016/j.cej.2024.154827

[36] D.T. Wasan, A.D. Nikolov, H. Brenner, Droplets speeding on surfaces. Science **291**(5504), 605–606 (2001). 10.1126/science.105846 11158664

[37] J. Liu, K. Pei, Y. Zhou, S. Fu, S. Ai et al., Bioinspired ultrasmall-bandgap MOF-integrated superhydrophobic textiles via in situ self-assembly: enabling next-generation multifunctional smart textiles. Adv. Funct. Mater. **36**(11), 13624 (2026). 10.1002/adfm.202513624

[38] F. Liang, W. Li, K. Zheng, K. Huang, S. Cheng et al., Graphene-skinned alumina fiber fabric for diverse electrothermal and electromagnetic compatibility and its mass production. Adv. Mater. **37**(28), 2501226 (2025). 10.1002/adma.202501226 40326189

[39] L. Wu, X. Bao, Z. Li, Y. Yu, Y. Liu et al., Rapid photothermal antibacterial and antifungal textiles through dynamic disulfide bond-assisted *in-situ* deposition of SeNPs. Chem. Eng. J. **479**, 147772 (2024). 10.1016/j.cej.2023.147772

[40] L. Wu, Z. Li, X. Bao, X. Cheng, C. Deng et al., Enzymatic redox-mediated fabrication of textiles with multimode synergistic antimicrobial activity through embedding nanosilver in dynamic polydisulfide networks. Adv. Funct. Mater. **35**(19), 2420046 (2025). 10.1002/adfm.202420046

[41] X. Bao, L. Wu, Y. Yu, B. Xu, L. Cui et al., A photothermal cellulose-based material for rapid anti-bacteria and HCHO removal via sequential formation of MnO_2_ coatings and amine-quinone networks. Chem. Eng. J. **468**, 143584 (2023). 10.1016/j.cej.2023.143584

[42] F. Wang, C.-B. Li, R.-Y. Sun, F. Song, W. Yang et al., A four-tier hierarchical architecture for superamphiphobic and flame-retardant fabrics with enhanced self-buoyancy and anti-icing properties. Chem. Eng. J. **488**, 151084 (2024). 10.1016/j.cej.2024.151084

[43] C. Xiang, C. Shen, K. Liang, Q. Gan, Y. Gao et al., Asymmetric wettability-photothermal fabric with fog capture and solar interfacial evaporation for switchable all-weather freshwater harvesting. Chem. Eng. J. **527**, 171335 (2026). 10.1016/j.cej.2025.171335

[44] D. Zhang, Z. Bai, J. Jiang, C. Ma, J. Guo et al., Biomimetic design of capillary-driven photothermal fabric for efficient interface evaporation. Small **21**(35), 2504092 (2025). 10.1002/smll.202504092 40613430

[45] Y. Peng, Y. Cui, Advanced textiles for personal thermal management and energy. Joule **4**(4), 724–742 (2020). 10.1016/j.joule.2020.02.011

[46] S. Hao, H. Han, Z. Yang, M. Chen, Y. Jiang et al., Recent advancements on photothermal conversion and antibacterial applications over MXenes-based materials. Nano-Micro Lett. **14**(1), 178 (2022). 10.1007/s40820-022-00901-w PMC9402885 36001173

[47] H. Zhou, Q. Li, Z. Zhang, X. Wang, H. Niu, Recent advances in superhydrophobic and antibacterial cellulose-based fibers and fabrics: bio-inspiration, strategies, and applications. Adv. Fiber Mater. **5**(5), 1555–1591 (2023). 10.1007/s42765-023-00297-1 PMC10201051 37361104

[48] R. Ma, D. Li, C. Xu, J. Yang, J. Huang et al., Fabricated advanced textile for personal thermal management, intelligent health monitoring and energy harvesting. Adv. Colloid Interface Sci. **332**, 103252 (2024). 10.1016/j.cis.2024.103252 39053159

[49] Y. Peng, Y. Cui, Thermal management with innovative fibers and textiles: manipulating heat transport, storage and conversion. Natl. Sci. Rev. **11**(10), nwae295 (2024). 10.1093/nsr/nwae295 PMC11879428 40041618

[50] Y. Si, D. Wang, Y. Jiang, H. Zhu, S. Shi et al., Bionic intelligent clothing. Adv. Mater. **38**(5), e14621 (2026). 10.1002/adma.202514621 41178143

[51] Q. Liu, Z. Zhu, H. Zhang, L. Zhou, H. Su et al., Advanced warming textiles for personalized thermal management through radiative, insulating, and electrothermal strategies. Mater. Today Energy **56**, 102192 (2026). 10.1016/j.mtener.2026.102192

[52] T. Xue, X. Zhang, C. Guo, R. Ma, W. Tao et al., Emerging thermoregulating textile for energy exchange and personal thermal management. Mater. Today Phys. **62**, 102048 (2026). 10.1016/j.mtphys.2026.102048

[53] Q. Ge, J. Chu, W. Cao, F. Yi, Z. Ran et al., Graphene‐based textiles for thermal management and flame retardancy. Adv. Funct. Mater. **32**(42), 2205934 (2022). 10.1002/adfm.202205934

[54] R. Liu, K. Xia, T. Yu, F. Gao, Q. Zhang et al., Multifunctional smart fabrics with integration of self-cleaning, energy harvesting, and thermal management properties. ACS Nano **18**(45), 31085–31097 (2024). 10.1021/acsnano.4c08324 39480157

[55] L. Feng, K. Wang, A. Xi, Z. Guo, X. Wang et al., Wearable radiative cooling fabrics for personal thermal management. Adv. Mater. Technol. (2025). 10.1002/admt.202501351

[56] H. Zheng, H. Xue, C. Xiao, J. Zhao, Y. Ding, Applications of passive radiative cooling technology in textile materials. Sol. Energy Mater. Sol. Cells **304**, 114418 (2026). 10.1016/j.solmat.2026.114418

[57] A.K. Kota, G. Kwon, A. Tuteja, The design and applications of superomniphobic surfaces. NPG Asia Mater. **6**(7), e109–e109 (2014). 10.1038/am.2014.34

[58] R.N. Wenzel, Surface roughness and contact angle. J. Phys. Colloid Chem. **53**(9), 1466–1467 (1949). 10.1021/j150474a015

[59] R.N. Wenzel, Resistance of solid surfaces to wetting by water. Ind. Eng. Chem. **28**(8), 988–994 (1936). 10.1021/ie50320a024

[60] H.J. Ensikat, P. Ditsche-Kuru, C. Neinhuis, W. Barthlott, Superhydrophobicity in perfection: the outstanding properties of the lotus leaf. Beilstein J. Nanotechnol. **2**(1), 152–161 (2011). 10.3762/bjnano.2.19 PMC3148040 21977427

[61] J. Ju, H. Bai, Y. Zheng, T. Zhao, R. Fang et al., A multi-structural and multi-functional integrated fog collection system in cactus. Nat. Commun. **3**(1), 1247 (2012). 10.1038/ncomms2253 PMC3535335 23212376

[62] L. Zhai, M.C. Berg, F.Ç. Cebeci, Y. Kim, J.M. Milwid et al., Patterned superhydrophobic surfaces: toward a synthetic mimic of the Namib desert beetle. Nano Lett. **6**(6), 1213–1217 (2006). 10.1021/nl060644q 16771582

[63] X. Gao, X. Yan, X. Yao, L. Xu, K. Zhang et al., The dry-style antifogging properties of mosquito compound eyes and artificial analogues prepared by soft lithography. Adv. Mater. **19**(17), 2213–2217 (2007). 10.1002/adma.200601946

[64] Z.-G. Guo, W.-M. Liu, B.-L. Su, A stable lotus-leaf-like water-repellent copper. Appl. Phys. Lett. **92**(6), 063104 (2008). 10.1063/1.2841666

[65] A.B.D. Cassie, S. Baxter, Wettability of porous surfaces. Trans. Faraday Soc. **40**, 546 (1944). 10.1039/tf9444000546

[66] W. Choi, A. Tuteja, S. Chhatre, J.M. Mabry, R.E. Cohen et al., Fabrics with tunable oleophobicity. Adv. Mater. **21**(21), 2190–2195 (2009). 10.1002/adma.200802502

[67] J. Lim, N. Powell, H. Lee, S. Michielsen, Integration of yarn compression in modeling structural geometry of liquid resistant–repellent fabric surfaces and its impact on liquid behavior. J. Mater. Sci. **51**(15), 7199–7210 (2016). 10.1007/s10853-016-0001-x

[68] J.A. Kleingartner, S. Srinivasan, Q.T. Truong, M. Sieber, R.E. Cohen et al., Designing robust hierarchically textured oleophobic fabrics. Langmuir **31**(48), 13201–13213 (2015). 10.1021/acs.langmuir.5b03000 26473386

[69] S. Shukla, Y. Wen, I. Szenti, A. Kukovecz, T.C. Sykes et al., Synergizing weave-architected wettability with hierarchical fibrous assemblies to design hydrophobic woven fabrics. Mater. Des. **258**, 114519 (2025). 10.1016/j.matdes.2025.114519

[70] E. Bormashenko, Wetting transitions on biomimetic surfaces. Philos. Trans. R. Soc. Math. Phys. Eng. Sci. **368**(1929), 4695–4711 (2010). 10.1098/rsta.2010.0121 20855316

[71] E.R. Jerison, Y. Xu, L.A. Wilen, E.R. Dufresne, Deformation of an elastic substrate by a three-phase contact line. Phys. Rev. Lett. **106**(18), 186103 (2011). 10.1103/PhysRevLett.106.186103 21635105

[72] A. Marchand, S. Das, J.H. Snoeijer, B. Andreotti, Contact angles on a soft solid: from Young’s law to Neumann’s law. Phys. Rev. Lett. **109**(23), 236101 (2012). 10.1103/PhysRevLett.109.236101 23368226

[73] P. Qi, J. Xie, G. Xia, Y. Wang, J.H. Xin, Advanced bionic textile materials: from principles to functional applications. Adv. Mater. **37**(48), 02118 (2025). 10.1002/adma.202502118 40708313

[74] G. Zheng, Z. Zhao, A. Wang, H. Cheng, X. Bao et al., Near infrared laser precisely controlled ultrafast superhydrophilicity-to-hydrophobicity transition of copper sulfide nanosphere coating on cellulose fiber fabric. Int. J. Biol. Macromol. **290**, 138854 (2025). 10.1016/j.ijbiomac.2024.138854 39694363

[75] R.W. Style, E.R. Dufresne, Static wetting on deformable substrates, from liquids to soft solids. Soft Matter **8**(27), 7177 (2012). 10.1039/c2sm25540e

[76] R.W. Style, A. Jagota, C.-Y. Hui, E.R. Dufresne, Elastocapillarity: surface tension and the mechanics of soft solids. Annu. Rev. Condens. Matter Phys. **8**, 99–118 (2017). 10.1146/annurev-conmatphys-031016-025326

[77] E.W. Washburn, The dynamics of capillary flow. Phys. Rev. **17**(3), 273–283 (1921). 10.1103/PhysRev.17.273

[78] R.G. Picknett, R. Bexon, The evaporation of sessile or pendant drops in still air. J. Colloid Interface Sci. **61**(2), 336–350 (1977). 10.1016/0021-9797(77)90396-4

[79] H. Hu, R.G. Larson, Evaporation of a sessile droplet on a substrate. J. Phys. Chem. B **106**(6), 1334–1344 (2002). 10.1021/jp0118322

[80] X. Xie, X. Chen, P.A. Levkin, W. Feng, A reactive superhydrophobic platform for living photolithography. Adv. Mater. **34**(36), 2203619 (2022). 10.1002/adma.202203619 35839120

[81] N.J. Shirtcliffe, G. McHale, S. Atherton, M.I. Newton, An introduction to superhydrophobicity. Adv. Colloid Interface Sci. **161**(1–2), 124–138 (2010). 10.1016/j.cis.2009.11.001 19944399

[82] S. Wang, K. Liu, X. Yao, L. Jiang, Bioinspired surfaces with superwettability: new insight on theory, design, and applications. Chem. Rev. **115**(16), 8230–8293 (2015). 10.1021/cr400083y 26244444

[83] P. Shi, C. Chen, Z. Wang, W. Hong, S. Duan et al., Air plastron–enabled heat management for enhanced photothermal actuation in underwater soft robots. Sci. Adv. **11**(33), eadx7189 (2025). 10.1126/sciadv.adx7189 PMC12356254 40815658

[84] Y. Hang, A. Wang, N. Wu, Plasmonic silver and gold nanoparticles: shape- and structure-modulated plasmonic functionality for point-of-caring sensing, bio-imaging and medical therapy. Chem. Soc. Rev. **53**(6), 2932–2971 (2024). 10.1039/D3CS00793F PMC11849058 38380656

[85] D. Mateo, J.L. Cerrillo, S. Durini, J. Gascon, Fundamentals and applications of photo-thermal catalysis. Chem. Soc. Rev. **50**(3), 2173–2210 (2021). 10.1039/D0CS00357C 33336654

[86] G. Mie, Beiträge zur Optik trüber Medien, speziell kolloidaler Metallösungen. Ann. Phys. **330**(3), 377–445 (1908). 10.1002/andp.19083300302

[87] K.L. Kelly, E. Coronado, L.L. Zhao, G.C. Schatz, The optical properties of metal nanoparticles: the influence of size, shape, and dielectric environment. J. Phys. Chem. B **107**(3), 668–677 (2003). 10.1021/jp026731y

[88] S. Link, M.A. El-Sayed, Shape and size dependence of radiative, non-radiative and photothermal properties of gold nanocrystals. Int. Rev. Phys. Chem. **19**(3), 409–453 (2000). 10.1080/01442350050034180

[89] B. Nikoobakht, M.A. El-Sayed, Preparation and growth mechanism of gold nanorods (NRs) using seed-mediated growth method. Chem. Mater. **15**(10), 1957–1962 (2003). 10.1021/CM020732L

[90] J. Hodak, I. Martini, G.V. Hartland, Ultrafast study of electron–phonon coupling in colloidal gold particles. Chem. Phys. Lett. **284**(1), 135–141 (1998). 10.1016/S0009-2614(97)01369-9

[91] G. Baffou, R. Quidant, Thermo‐plasmonics: using metallic nanostructures as nano‐sources of heat. Laser Photon. Rev. **7**(2), 171–187 (2013). 10.1002/lpor.201200003

[92] G.V. Hartland, Optical studies of dynamics in noble metal nanostructures. Chem. Rev. **111**(6), 3858–3887 (2011). 10.1021/cr1002547 21434614

[93] E. Pakdel, J. Sharp, S. Kashi, W. Bai, M.P. Gashti et al., Antibacterial superhydrophobic cotton fabric with photothermal, self-cleaning, and ultraviolet protection functionalities. ACS Appl. Mater. Interfaces **15**(28), 34031–34043 (2023). 10.1021/acsami.3c04598 37399520

[94] N. Hu, Z. Zhu, X. Cai, P. Müller-Buschbaum, Q. Zhong, Enhanced anti-bacterial properties and thermal regulation via photothermal conversion with localized surface plasmon resonance effect in cotton fabrics. J. Colloid Interface Sci. **681**, 25–34 (2025). 10.1016/j.jcis.2024.11.138 39591852

[95] C.-C. Chuang, H.-C. Chu, S.-B. Huang, W.-S. Chang, H.-Y. Tuan, Laser-induced plasmonic heating in copper nanowire fabric as a photothermal catalytic reactor. Chem. Eng. J. **379**, 122285 (2020). 10.1016/j.cej.2019.122285

[96] H. Li, S. Tang, W. Chen, X. Yang, S. Dong et al., Robust multifunctional superhydrophobic, photocatalytic and conductive fabrics with electro-/photo-thermal self-healing ability. J. Colloid Interface Sci. **614**, 1–11 (2022). 10.1016/j.jcis.2022.01.090 35078081

[97] Q. Liu, C. Sun, Q. He, A. Khalil, T. Xiang et al., Stable metallic 1T-WS_2_ ultrathin nanosheets as a promising agent for near-infrared photothermal ablation cancer therapy. Nano Res. **8**(12), 3982–3991 (2015). 10.1007/s12274-015-0901-0

[98] B.T. Diroll, A. Brumberg, A.A. Leonard, S. Panuganti, N.E. Watkins et al., Photothermal behaviour of titanium nitride nanoparticles evaluated by transient X-ray diffraction. Nanoscale **13**(4), 2658–2664 (2021). 10.1039/D0NR08202C 33496308

[99] C. Alvarez, C. Berrospe-Rodriguez, C. Wu, J. Pasek-Allen, K. Khosla et al., Photothermal heating of titanium nitride nanomaterials for fast and uniform laser warming of cryopreserved biomaterials. Front. Bioeng. Biotechnol. **10**, 957481 (2022). 10.3389/fbioe.2022.957481 PMC9455577 36091458

[100] C.H. Henry, D.V. Lang, Nonradiative capture and recombination by multiphonon emission in GaAs and GaP. Phys. Rev. B **15**(2), 989–1016 (1977). 10.1103/PhysRevB.15.989

[101] R.N. Hall, Electron-hole recombination in germanium. Phys. Rev. **87**(2), 387–387 (1952). 10.1103/PhysRev.87.387

[102] W. Shockley, W.T. Read, Statistics of the recombinations of holes and electrons. Phys. Rev. **87**(5), 835–842 (1952). 10.1103/PhysRev.87.835

[103] P. Makuła, M. Pacia, W. Macyk, How to correctly determine the band gap energy of modified semiconductor photocatalysts based on UV–Vis spectra. J. Phys. Chem. Lett. **9**(23), 6814–6817 (2018). 10.1021/acs.jpclett.8b02892 30990726

[104] H. Li, Z. Sha, Y. Song, J. Fan, J. Wei et al., Developing wearable photothermal and antibacterial fabrics: in-situ polymerization of MoS_2_-hybridized PET fibers. Compos. Sci. Technol. **270**, 111291 (2025). 10.1016/j.compscitech.2025.111291

[105] L. Zhang, X. Qi, H.-Y. Yu, T.-T. Li, H.-Y. Wang et al., TiN-based photothermal fabric with a superhydrophobic surface for synergistic anti-/deicing. Langmuir **41**(35), 23888–23898 (2025). 10.1021/acs.langmuir.5c03265 40851375

[106] Y. Zhao, W. Zhang, H. Cai, Y. Xu, X. Han, One-step lignin-mediated co-assembly of graphene oxide on cotton fabrics for durable conductive and photothermal applications. Chem. Eng. J. **527**, 172229 (2026). 10.1016/j.cej.2025.172229

[107] L. Hu, X. Li, L. Ding, L. Chen, X. Zhu et al., Flexible textiles with polypyrrole deposited phase change microcapsules for efficient photothermal energy conversion and storage. Sol. Energy Mater. Sol. Cells **224**, 110985 (2021). 10.1016/j.solmat.2021.110985

[108] N.W.S. Kam, M. O’Connell, J.A. Wisdom, H. Dai, Carbon nanotubes as multifunctional biological transporters and near-infrared agents for selective cancer cell destruction. Proc. Natl. Acad. Sci. U. S. A. **102**(33), 11600–11605 (2005). 10.1073/pnas.0502680102 PMC1187972 16087878

[109] R.R. Nair, P. Blake, A.N. Grigorenko, K.S. Novoselov, T.J. Booth et al., Fine structure constant defines visual transparency of graphene. Science **320**(5881), 1308–1308 (2008). 10.1126/science.1156965 18388259

[110] M. Chen, X. Fang, S. Tang, N. Zheng, Polypyrrole nanoparticles for high-performance in vivo near-infrared photothermal cancer therapy. Chem. Commun. **48**(71), 8934–8936 (2012). 10.1039/C2CC34463G 22847451

[111] J.M. André, D.P. Vercauteren, G.B. Street, J.L. Brédas, Electronic properties of polypyrrole: an ab initio Hartree–Fock study. J. Chem. Phys. **80**(11), 5643–5648 (1984). 10.1063/1.446630

[112] J.L. Brédas, B. Thémans, J.M. André, Valence effective hamiltonian technique for nitrogen containing polymers: electronic structure of polypyrrole, pyrolized polyacrylonitrile, paracyanogen, polymethineimine, and derivatives. J. Chem. Phys. **78**(10), 6137–6148 (1983). 10.1063/1.444576

[113] H. Wang, J.H. Strait, P.A. George, S. Shivaraman, V.B. Shields et al., Ultrafast relaxation dynamics of hot optical phonons in graphene. Appl. Phys. Lett. **96**(8), 081917 (2010). 10.1063/1.3291615

[114] D. Sun, Z.-K. Wu, C. Divin, X. Li, C. Berger et al., Ultrafast relaxation of excited dirac fermions in epitaxial graphene using optical differential transmission spectroscopy. Phys. Rev. Lett. **101**(15), 157402 (2008). 10.1103/PhysRevLett.101.157402 18999638

[115] J.M. Dawlaty, S. Shivaraman, M. Chandrashekhar, F. Rana, M.G. Spencer, Measurement of ultrafast carrier dynamics in epitaxial graphene. Appl. Phys. Lett. **92**(4), 042116 (2008). 10.1063/1.2837539 19367881

[116] H. Yang, X. Cheng, L. Zhao, X. Zhang, B. Lu et al., Highly efficient and stable all-weather moisture power generation via asymmetric structured color photothermal evaporation-induced cotton fabrics. Adv. Mater. **38**, e18736 (2025). 10.1002/adma.202518736 41454701

[117] Z. Lian, J. Zhou, Z. Liu, Y. Wan, R. Liu et al., Hierarchically structured superhydrophobic surfaces with photothermal conversion to avoid icing. Int. J. Mech. Sci. **275**, 109341 (2024). 10.1016/j.ijmecsci.2024.109341

[118] F. Yao, J. Wei, Y. Xu, H. Tu, M. Huang et al., Light-trapping superhydrophobic coatings with switchable wettability to solve low-temperature anti-icing/deicing and high-temperature overheating problems on surfaces. J. Colloid Interface Sci. **699**, 138275 (2025). 10.1016/j.jcis.2025.138275 40578085

[119] H. Lu, L. Chen, X. Sha, G. Chen, Multi-functional superhydrophobic photothermal cotton fabric inspired by mussels with anti-icing/de-icing and oil-water separation functions. Surf. Coat. Technol. **515**, 132667 (2025). 10.1016/j.surfcoat.2025.132667

[120] X. Wei, J. Wei, Y. Feng, J. Wang, Photothermal hydrophobic coating with light-trapping and thermal isolated effects for efficient photothermal anti-icing/de-icing. Prog. Org. Coat. **179**, 107550 (2023). 10.1016/j.porgcoat.2023.107550

[121] Z. Xie, H. Wang, M. Li, Y. Tian, Q. Deng et al., Photothermal trap with multi-scale micro-nano hierarchical structure enhances light absorption and promote photothermal anti-icing/deicing. Chem. Eng. J. **435**, 135025 (2022). 10.1016/j.cej.2022.135025

[122] E. Yablonovitch, Statistical ray optics. J. Opt. Soc. Am. **72**(7), 899 (1982). 10.1364/JOSA.72.000899

[123] C.F. Bohren, D.R. Huffman, *Absorption and Scattering of Light by Small Particles* (Wiley-CH Verlag GmbH & Co KGaA, New York, 2004)

[124] Z. Zhou, S. Tan, W. Sun, X. Guan, T. Xu et al., A cost-effective photothermal superhydrophobic coating with micro- and nano-graded structures for efficient solar energy harvesting. J. Mater. Sci. Technol. **198**, 158–165 (2024). 10.1016/j.jmst.2024.02.029

[125] H. Zhong, C. Xiang, Z. Hu, X. Yang, H. Liu et al., Plasmonic photothermal superhydrophobic surface with nanotubes thermal insulating blanket for anti-icing and anti-frosting under weak light illumination. Mater. Today Phys. **50**, 101625 (2025). 10.1016/j.mtphys.2024.101625

[126] M. Mao, J. Wei, B. Li, L. Li, X. Huang et al., Scalable robust photothermal superhydrophobic coatings for efficient anti-icing and de-icing in simulated/real environments. Nat. Commun. **15**(1), 9610 (2024). 10.1038/s41467-024-54058-8 PMC11541590 39505855

[127] S. Peng, X. Xiao, J. Wei, J. Wang, Superhydrophobic coating with electro-photo-thermal conversion properties for all-weather anti-icing. Sol. Energy **270**, 112384 (2024). 10.1016/j.solener.2024.112384

[128] X. Yang, Y. Liu, Y. Zhong, H. Chen, Ultra-durable photothermal anti-/de-icing superhydrophobic coating with water droplets freezing from the outside in. J. Colloid Interface Sci. **682**, 1127–1139 (2025). 10.1016/j.jcis.2024.12.044 39667332

[129] B. Luo, C. Dong, W. Lin, D. Zhang, Y. Zhang et al., Durable photothermal superhydrophobic coatings engineered on porous foam for efficient ice melting. Surf. Interfaces **78**, 108090 (2025). 10.1016/j.surfin.2025.108090

[130] X. Xiao, J. Wei, J. Wang, Photothermal superhydrophobic foam with ‘cage’ characteristic shows excellent weak-light deicing and durable superhydrophobicity. Prog. Org. Coat. **189**, 108304 (2024). 10.1016/j.porgcoat.2024.108304

[131] Q. Zhang, T. Shi, L. Zhu, N. Cao, X. Chen, SiO_2_@MXene-based fabric featuring low heat loss for high-efficiency solar-driven freshwater production. J. Environ. Chem. Eng. **12**(3), 112658 (2024). 10.1016/j.jece.2024.112658

[132] L. Wu, P. Liu, X. Hua, Z. Guo, W. Liu, Photothermal superhydrophobic membrane based on breath figure: anti-icing and deicing. Chem. Eng. J. **480**, 147553 (2024). 10.1016/j.cej.2023.147553

[133] H. He, W. Huang, Z. Guo, Superhydrophobic and photothermal SiC/TiN durable composite coatings for passive anti-icing/active de-icing and de-frosting. Mater. Today Phys. **30**, 100927 (2023). 10.1016/j.mtphys.2022.100927

[134] Y. Guo, H. Zhao, C. Zhang, G. Zhao, Super photothermal/electrothermal response and anti-icing/deicing capability of superhydrophobic multi-walled carbon nanotubes/epoxy coating. Chem. Eng. J. **497**, 154383 (2024). 10.1016/j.cej.2024.154383

[135] J. Zhu, W. Li, T. Wang, H. Feng, J. Cheng et al., Robust photothermal self-healing superhydrophobic composite coating for durable anti/de-icing applications. Chem. Eng. J. **505**, 159218 (2025). 10.1016/j.cej.2025.159218

[136] W. Sun, Y. Wei, Y. Feng, F. Chu, Anti-icing and deicing characteristics of photothermal superhydrophobic surfaces based on metal nanoparticles and carbon nanotube materials. Energy **286**, 129656 (2024). 10.1016/j.energy.2023.129656

[137] H. He, Z. Guo, Fabric-based superhydrophobic MXene@ polypyrrole heater with superior dual-driving energy conversion. J. Colloid Interface Sci. **629**, 508–521 (2023). 10.1016/j.jcis.2022.08.176 36088696

[138] Y. Wu, L. Dong, X. Shu, L. Chen, Y. Zhang et al., Fluorine-free photothermal superhydrophobic fabrics with self-healing and ice-resistant properties for cold-environment protection. Mater. Des. **256**, 114262 (2025). 10.1016/j.matdes.2025.114262

[139] X. Xie, C. Zhen, F. Xiu, T. Quan, G. Yao et al., Photothermal superhydrophobic textiles with antibacterial activity based on synergistic “repel-and-kill” mechanism. Mater. Today Nano **30**, 100622 (2025). 10.1016/j.mtnano.2025.100622

[140] S. Lee, M.J. Bae, E.J. Seo, J. Lyu, S.-H. Lee et al., Photothermally promoted recovery of a superhydrophobic surface with anti-icing and de-icing properties for outdoor applications. Prog. Org. Coat. **189**, 108298 (2024). 10.1016/j.porgcoat.2024.108298

[141] D. Tam, V. Von Arnim, G.H. McKINLEY, A.E. Hosoi, Marangoni convection in droplets on superhydrophobic surfaces. J. Fluid Mech. **624**, 101–123 (2009). 10.1017/S0022112008005053

[142] S. Semenov, V.M. Starov, R.G. Rubio, M.G. Velarde, Computer simulations of evaporation of pinned sessile droplets: influence of kinetic effects. Langmuir **28**(43), 15203–15211 (2012). 10.1021/la303916u 23046501

[143] A. Al-Sharafi, B.S. Yilbas, H. Al-Qahtani, Heating enhancement of a droplet on a superhydrophobic surface. Sci. Rep. **10**(1), 4594 (2020). 10.1038/s41598-020-61532-y PMC7067816 32165714

[144] A. Phadnis, K. Rykaczewski, The effect of Marangoni convection on heat transfer during dropwise condensation on hydrophobic and omniphobic surfaces. Int. J. Heat Mass Transf. **115**, 148–158 (2017). 10.1016/j.ijheatmasstransfer.2017.08.026

[145] A. Al-Sharafi, H. Ali, B.S. Yilbas, A.Z. Sahin, N. Al-Aqeeli et al., Internal flow and heat transfer in a droplet located on a superhydrophobic surface. Int. J. Therm. Sci. **121**, 213–227 (2017). 10.1016/j.ijthermalsci.2017.07.016

[146] A. Al-Sharafi, B.S. Yilbas, H. Ali, Droplet heat transfer on micro-post arrays: effect of droplet size on droplet thermal characteristics. Int. J. Heat Fluid Flow **68**, 62–78 (2017). 10.1016/j.ijheatfluidflow.2017.09.012

[147] M.J. Gibbons, P. Di Marco, A.J. Robinson, Local heat transfer to an evaporating superhydrophobic droplet. Int. J. Heat Mass Transf. **121**, 641–652 (2018). 10.1016/j.ijheatmasstransfer.2018.01.007

[148] J. Liu, W. Huang, G. Cui, X. Xing, J. Liu, NIR-responsive self-healing superhydrophobic coatings: enhanced, corrosion resistance, and mechanical stability. Process. Saf. Environ. Prot. **195**, 106759 (2025). 10.1016/j.psep.2025.01.013

[149] R. Chen, L. Jiao, X. Zhu, Q. Liao, D. Ye et al., Cassie-to-Wenzel transition of droplet on the superhydrophobic surface caused by light induced evaporation. Appl. Therm. Eng. **144**, 945–959 (2018). 10.1016/j.applthermaleng.2018.09.011

[150] S. Xiang, W. Liu, Self-healing superhydrophobic surfaces: healing principles and applications. Adv. Mater. Interfaces **8**(12), 2100247 (2021). 10.1002/admi.202100247

[151] P. Yu, Z. Yu, K. Liao, S. Xia, K. Li et al., Self-healing superhydrophobic anti-corrosion coating with dual photo-thermal and pH response for effective anti-icing and de-icing. Appl. Surf. Sci. **701**, 163295 (2025). 10.1016/j.apsusc.2025.163295

[152] M.L. Williams, R.F. Landel, J.D. Ferry, The temperature dependence of relaxation mechanisms in amorphous polymers and other glass-forming liquids. J. Am. Chem. Soc. **77**(14), 3701–3707 (1955). 10.1021/ja01619a008

[153] V.N. Novikov, A.P. Sokolov, Temperature dependence of structural relaxation in glass-forming liquids and polymers. Entropy **24**(8), 1101 (2022). 10.3390/e24081101 PMC9407199 36010765

[154] A. Duan, F. Gu, X. Jiang, J. Du, D. Shao et al., Washable superhydrophobic cotton fabric with photothermal self-healing performance based on nanocrystal-MXene. ACS Appl. Mater. Interfaces **17**(6), 9923–9936 (2025). 10.1021/acsami.4c21715 39874590

[155] M. Rubinstein, R.H. Colby, *Polymer Physics *(Oxford University Press, Oxford, 2023)

[156] Y. Wang, Y. Lian, M. Zhang, S. Zhao, Y. Li et al., Robust self-healing superhydrophobic coating for anti-icing. J. Colloid Interface Sci. **708**, 139836 (2026). 10.1016/j.jcis.2026.139836 41512524

[157] Y. Xue, P. Verdross, W. Liang, R.T. Woodward, A. Bismarck, Breaking the ice: applications of photothermal superhydrophobic materials for efficient deicing strategies. Adv. Colloid Interface Sci. **341**, 103489 (2025). 10.1016/j.cis.2025.103489 40168712

[158] T. Zhang, J. Deng, L.-Z. Zhang, A photothermal self-healing superhydrophobic coating with anti-frosting and anti-corrosion properties. Prog. Org. Coat. **180**, 107569 (2023). 10.1016/j.porgcoat.2023.107569

[159] H.-T. Ren, C.-C. Cai, W.-B. Cao, D.-S. Li, T.-T. Li et al., Superhydrophobic TiN-coated cotton fabrics with nanoscale roughness and photothermal self-healing properties for effective oil–water separation. ACS Appl. Nano Mater. **6**(13), 11925–11933 (2023). 10.1021/acsanm.3c01763

[160] C. Li, P. Wang, D. Zhang, S. Wang, Near-infrared responsive smart superhydrophobic coating with self-healing and robustness enhanced by disulfide-bonded polyurethane. ACS Appl. Mater. Interfaces **14**(40), 45988–46000 (2022). 10.1021/acsami.2c08496 36135324

[161] M. Gardette, A. Perthue, J.-L. Gardette, T. Janecska, E. Földes et al., Photo- and thermal-oxidation of polyethylene: comparison of mechanisms and influence of unsaturation content. Polym. Degrad. Stab. **98**(11), 2383–2390 (2013). 10.1016/j.polymdegradstab.2013.07.017

[162] J. Manna, S. Goswami, N. Shilpa, N. Sahu, R.K. Rana, Biomimetic method to assemble nanostructured Ag@ZnO on cotton fabrics: application as self-cleaning flexible materials with visible-light photocatalysis and antibacterial activities. ACS Appl. Mater. Interfaces **7**(15), 8076–8082 (2015). 10.1021/acsami.5b00633 25823715

[163] Y. Fu, B. Jin, Q. Zhang, X. Zhan, F. Chen, pH-induced switchable superwettability of efficient antibacterial fabrics for durable selective oil/water separation. ACS Appl. Mater. Interfaces **9**(35), 30161–30170 (2017). 10.1021/acsami.7b09159 28805055

[164] G. Li, G. Hong, D. Dong, W. Song, X. Zhang, Multiresponsive graphene‐aerogel‐directed phase‐change smart fibers. Adv. Mater. **30**(30), 1801754 (2018). 10.1002/adma.201801754 29904953

[165] S. Zhang, F. Zhang, Z. Zhang, G. Li, H. Fu et al., An electroless nickel plating fabric coated with photothermal Chinese ink for powerful passive anti-icing/icephobic and fast active deicing. Chem. Eng. J. **450**, 138328 (2022). 10.1016/j.cej.2022.138328

[166] J. Hu, H. Li, Z. Liu, G. Jiang, Functionalized superhydrophobic quartz fabric with electro-photo-thermal conversion performance: designed for low-cost and efficient self-heating deicing. Surf. Coat. Technol. **425**, 127646 (2021). 10.1016/j.surfcoat.2021.127646

[167] Y. Deng, F. Du, Z. Chen, P. Lv, Z. Yin et al., Functionalized superhydrophobic coatings with electro-photothermal effect for all-day durable anti-icing. Adv. Mater. Interfaces **11**(11), 2300869 (2024). 10.1002/admi.202300869

[168] J. Wang, P. Du, J. Zhang, L. Zeng, Y. Chen et al., Janus textile for multifunctional personal thermal management via dual photothermal–electrothermal conversion. ACS Appl. Mater. Interfaces **17**(23), 34569–34581 (2025). 10.1021/acsami.5c06576 40440443

[169] J. Luo, L. Huo, L. Wang, X. Huang, J. Li et al., Superhydrophobic and multi-responsive fabric composite with excellent electro-photo-thermal effect and electromagnetic interference shielding performance. Chem. Eng. J. **391**, 123537 (2020). 10.1016/j.cej.2019.123537

[170] M. Ju, K. Li, M. Wu, A. Hou, K. Xie et al., A flexible, visible-light-driven efficient photodynamic antibacterial and UV-protective silk protein material assembled by the cationic phthalocyanine nanodots photosensitizers. Chem. Eng. J. **525**, 170253 (2025). 10.1016/j.cej.2025.170253

[171] T. Wei, L. Xu, Q. Cheng, J. Cai, J. Yu et al., In situ grown polypyrrole and PDMS coatings enable superhydrophobic silk fabrics with photothermal antimicrobial activity and high-efficiency electromagnetic shielding. Adv. Compos. Hybrid Mater. **9**(1), 57 (2026). 10.1007/s42114-025-01594-6

[172] S. Hou, X. Wang, G. Li, D. Zhang, X. Qian et al., Lizard skin-inspired superhydrophobic photothermal fabric with hierarchical structure for anti/de-icing coverings. Colloids Surf A Physicochem Eng Asp **722**, 137238 (2025). 10.1016/j.colsurfa.2025.137238

[173] X. Zhang, N. Yu, Q. Ren, S. Niu, L. Zhu et al., Janus nanofiber membranes with photothermal‐enhanced biofluid drainage and sterilization for diabetic wounds. Adv. Funct. Mater. **34**(24), 2315020 (2024). 10.1002/adfm.202315020

[174] J. Ryu, K. Kim, J. Park, B.G. Hwang, Y. Ko et al., Nearly perfect durable superhydrophobic surfaces fabricated by a simple one-step plasma treatment. Sci. Rep. **7**(1), 1981 (2017). 10.1038/s41598-017-02108-1 PMC5434029 28512304

[175] W. Wang, L. Lu, D. Zhang, Y. Yao, Y. Xie, Experimental and modeling study of laser induced silicon carbide/graphene on cotton cloth for superhydrophobic applications. Opt. Laser Technol. **158**, 108782 (2023). 10.1016/j.optlastec.2022.108782

[176] H.-B. Yuan, M. Zhao, X. Zhu, D. Sha, G. Chen et al., Facile fabrication of durable and breathable superhydrophobic cotton fabric for self-cleaning, UV-blocking, anti-icing, and photothermal de-icing. Cellulose **31**(7), 4627–4644 (2024). 10.1007/s10570-024-05867-z

[177] A. Li, B. Chen, J. Wu, J. Gao, J. Zhang et al., Ultra-durable Janus fabric treated with femtosecond laser for personal anti-fouling and thermal management. Polym. Test. **147**, 108815 (2025). 10.1016/j.polymertesting.2025.108815

[178] H. Chen, W. Zhang, W. Gao, G. Zhang, Plasma-induced amino HBP/Ag nanoparticle-grafted PP melt-blown nonwoven fabric and its antibacterial performance. Coatings **15**(8), 947 (2025). 10.3390/coatings15080947

[179] S. Kim, J.-H. Oh, C. Park, Development of energy-efficient superhydrophobic polypropylene fabric by oxygen plasma etching and thermal aging. Polymers **12**, 2756 (2020). 10.3390/polym12112756 PMC7700148 33238417

[180] K. Yin, L. Wang, Q. Deng, Q. Huang, J. Jiang et al., Femtosecond laser thermal accumulation-triggered micro-/nanostructures with patternable and controllable wettability towards liquid manipulating. Nano-Micro Lett. **14**(1), 97 (2022). 10.1007/s40820-022-00840-6 PMC8993985 35394233

[181] E. Eslami, R. Jafari, G. Momen, A review of plasma-based superhydrophobic textiles: theoretical definitions, fabrication, and recent developments. J. Coat. Technol. Res. **18**(6), 1635–1658 (2021). 10.1007/s11998-021-00523-8

[182] S.S. Palaskar, Adhesion properties of polypropylene fabric treated with atmospheric pressure plasma and coated with polyurethane: studies on ageing process. Int. J. Adhes. Adhes. **125**, 103428 (2023). 10.1016/j.ijadhadh.2023.103428

[183] T.-B. Yang, J.-Y. Zong, D.-Z. Jia, L. Xu, Y.-Y. Wang et al., Superhydrophobic stretchable conductive composite textile with weft-knitted structure for excellent electromagnetic interference shielding and Joule heating performance. Chem. Eng. J. **489**, 151360 (2024). 10.1016/j.cej.2024.151360

[184] G. Zheng, Z. Jiang, Y. Cui, M. Zhou, Y. Yu et al., Photothermal, superhydrophobic, conductive, and anti-UV cotton fabric loaded with polydimethylsiloxane-encapsulated copper sulfide nanoflowers. Int. J. Biol. Macromol. **265**, 130650 (2024). 10.1016/j.ijbiomac.2024.130650 38462099

[185] Y. Guan, W. Zhang, B. Bi, S. Cao, Z. Wang et al., Polypyrrole-mediated construction of superhydrophobic photo/electro thermal composite fabric for efficient crude oil dehydration and recovery. Desalination **591**, 117978 (2024). 10.1016/j.desal.2024.117978

[186] Y. Tian, Y. Chen, S. Wang, X. Wang, J. Yu et al., Ultrathin aerogel-structured micro/nanofiber metafabric via dual air-gelation synthesis for self-sustainable heating. Nat. Commun. **15**(1), 6416 (2024). 10.1038/s41467-024-50654-w PMC11289394 39079966

[187] J. Ma, J. Cao, Y. Zhang, H. Song, Y. Qing, Mechanically durable superhydrophobic textile-based strain sensor based on synergistically coupled dual conductive networks for long-term underwater motion monitoring. Chem. Eng. J. **504**, 158955 (2025). 10.1016/j.cej.2024.158955

[188] S.-L. Li, M.-Q. Chen, M.-L. Wu, Y.-D. Li, J.-B. Zeng, Sustainable conductive coating methodology toward superhydrophobic elastic fabric sensors for human motion monitoring, underwater sensing and photothermal conversion. Chem. Eng. J. **522**, 167266 (2025). 10.1016/j.cej.2025.167266

[189] J. Fang, C. Zhao, C. Meng, G. Zhang, Preparation and properties of PA@PANI conductive and flame retardant functional polyester fabric without droplet. Mater. Today Commun. **30**, 103031 (2022). 10.1016/j.mtcomm.2021.103031

[190] Z. Guo, Y. Wang, J. Huang, S. Zhang, R. Zhang et al., Multi-functional and water-resistant conductive silver nanoparticle-decorated cotton textiles with excellent joule heating performances and human motion monitoring. Cellulose **28**(11), 7483–7495 (2021). 10.1007/s10570-021-03955-y

[191] Z. Wang, H. Cheng, R. Chen, M. Wang, N. Jiang et al., Omnipotent antibacterial cotton fabrics with superhydrophobic and photothermal properties. Int. J. Biol. Macromol. **290**, 138901 (2025). 10.1016/j.ijbiomac.2024.138901 39706440

[192] J. Zhang, L. Zhang, X. Gong, Design and fabrication of polydopamine based superhydrophobic fabrics for efficient oil–water separation. Soft Matter **17**(27), 6542–6551 (2021). 10.1039/d1sm00647a 34151338

[193] S. Liu, Y. Wang, X. Tang, Z. Xu, C. Peng, Fabrication of polydimethylsiloxane superhydrophobic coatings with self-healing properties using the template method. J. Mater. Eng. Perform. **33**(3), 1349–1357 (2023). 10.1007/s11665-023-08074-2

[194] W. Li, Z. Yu, Y. Zhang, C. Lv, X. He et al., Scalable multifunctional MOFs-textiles via diazonium chemistry. Nat. Commun. **15**(1), 5297 (2024). 10.1038/s41467-024-49636-9 PMC11192900 38906900

[195] W. Li, Y. Zhang, Z. Yu, T. Zhu, J. Kang et al., In situ growth of a stable metal–organic framework (MOF) on flexible fabric via a layer-by-layer strategy for versatile applications. ACS Nano **16**(9), 14779–14791 (2022). 10.1021/acsnano.2c05624 36103395

[196] H.-J. Butt, J. Liu, K. Koynov, B. Straub, C. Hinduja et al., Contact angle hysteresis. Curr. Opin. Colloid Interface Sci. **59**, 101574 (2022). 10.1016/j.cocis.2022.101574

[197] W. Barthlott, C. Neinhuis, Purity of the sacred lotus, or escape from contamination in biological surfaces. Planta **202**(1), 1–8 (1997). 10.1007/s004250050096

[198] D. Quéré, Rough ideas on wetting. Phys. Stat. Mech. Its Appl. **313**(1), 32–46 (2002). 10.1016/S0378-4371(02)01033-6

[199] T. Huhtamäki, X. Tian, J.T. Korhonen, R.H.A. Ras, Surface-wetting characterization using contact-angle measurements. Nat. Protoc. **13**(7), 1521–1538 (2018). 10.1038/s41596-018-0003-z 29988109

[200] J. Bico, U. Thiele, D. Quéré, Wetting of textured surfaces. Colloids Surf. Physicochem. Eng. Asp. **206**(1), 41–46 (2002). 10.1016/S0927-7757(02)00061-4

[201] C. Zhang, Y. Lei, K. Wang, B. Jiang, G. Ye et al., Durable fluorine-free multilayer superhydrophobic coatings for synergistic photothermal and electrothermal anti-icing protection. Prog. Org. Coat. **208**, 109433 (2025). 10.1016/j.porgcoat.2025.109433

[202] J. Wei, J. Zhang, X. Cao, J. Huo, X. Huang et al., Durable superhydrophobic coatings for prevention of rain attenuation of 5G/weather radomes. Nat. Commun. **14**(1), 2862 (2023). 10.1038/s41467-023-38678-0 PMC10198997 37208369

[203] C. Jia, J. Zhu, Y. Chen, K. Yang, J. He, Fe_3_O_4_/carbon black superhydrophbic coating exhibiting photothermal and efficient solar-assisted deicing properties. Surf. Coat. Technol. **516**, 132735 (2025). 10.1016/j.surfcoat.2025.132735

[204] H. Zhang, J. Ou, X. Fang, S. Lei, F. Wang et al., Robust superhydrophobic fabric via UV-accelerated atmospheric deposition of polydopamine and silver nanoparticles for solar evaporation and water/oil separation. Chem. Eng. J. **429**, 132539 (2022). 10.1016/j.cej.2021.132539

[205] B. Deng, R. Cai, Y. Yu, H. Jiang, C. Wang et al., Laundering durability of superhydrophobic cotton fabric. Adv. Mater. **22**(48), 5473–5477 (2010). 10.1002/adma.201002614 20941799

[206] X. Yang, H. Li, L. Li, M. Li, T. Xing et al., Fluorine-free, short-process and robust superhydrophobic cotton fabric and its oil-water separation ability. Prog. Org. Coat. **172**, 107122 (2022). 10.1016/j.porgcoat.2022.107122

[207] Y. Guo, C. Li, X. Li, H. Xu, W. Chen et al., Fabrication of superhydrophobic cotton fabric with multiple durability and wearing comfort via an environmentally friendly spraying method. Ind. Crops Prod. **194**, 116359 (2023). 10.1016/j.indcrop.2023.116359

[208] S. Zhu, T. Lu, Z. Wang, D. Peng, L. Wang et al., Durable superhydrophobic PDA-ZIF-8/PDMS composite fabric with photothermal antibacterial and self-cleaning properties. Mater. Lett. **360**, 135961 (2024). 10.1016/j.matlet.2024.135961

[209] D.K. Roper, W. Ahn, M. Hoepfner, Microscale heat transfer transduced by surface plasmon resonant gold nanoparticles. J. Phys. Chem. C **111**(9), 3636–3641 (2007). 10.1021/jp064341w PMC2583113 19011696

[210] C.A. Gueymard, D. Myers, K. Emery, Proposed reference irradiance spectra for solar energy systems testing. Sol. Energy **73**(6), 443–467 (2002). 10.1016/S0038-092X(03)00005-7

[211] S. Miao, Z. Xiong, J. Zhang, Y. Wu, X. Gong, Polydopamine/SiO_2_ hybrid structured superamphiphobic fabrics with good photothermal behavior. Langmuir **38**(30), 9431–9440 (2022). 10.1021/acs.langmuir.2c01629 35875891

[212] L. Zhang, Y. Luo, J. Xu, J. Liu, Bio-inspired photothermal superhydrophobic surface with good anti-/de-icing property and durability based on laser texturing. Surf. Coat. Technol. **512**, 132398 (2025). 10.1016/j.surfcoat.2025.132398

[213] M. Guo, Z. Li, Y. Lei, Y. Li, Y. Huang et al., Wearable heated fabrics with hierarchically self-assembled photothermal nanoparticles coatings for thermotherapy application. Fibers Polym. **24**(12), 4203–4212 (2023). 10.1007/s12221-023-00379-2

[214] R. Usamentiaga, P. Venegas, J. Guerediaga, L. Vega, J. Molleda et al., Infrared thermography for temperature measurement and non-destructive testing. Sensors **14**(7), 12305–12348 (2014). 10.3390/s140712305 PMC4168422 25014096

[215] D. Cheng, Y. Liu, Y. Zhang, J. Ran, S. Bi et al., Polydopamine-assisted deposition of CuS nanoparticles on cotton fabrics for photocatalytic and photothermal conversion performance. Cellulose **27**(14), 8443–8455 (2020). 10.1007/s10570-020-03358-5

[216] S. Liang, A.-Y. Duan, S.-K. Yan, Y.-R. Zhao, J. Zhang et al., Sunlight-activated photochromic-photothermal nanofibrous textiles for self-monitored thermal management. Chem. Eng. J. **522**, 167600 (2025). 10.1016/j.cej.2025.167600

[217] A.R. Bowman, J. Ma, F. Kiani, G. García Martínez, G. Tagliabue, Best practices in measuring absorption at the macro- and microscale. APL Photonics **9**(6), 061101 (2024). 10.1063/5.0210830

[218] L. Hanssen, Integrating-sphere system and method for absolute measurement of transmittance, reflectance, and absorptance of specular samples. Appl. Opt. **40**(19), 3196 (2001). 10.1364/AO.40.003196 11958259

[219] G.A. Zerlaut, T.E. Anderson, Multiple-integrating sphere spectrophotometer for measuring absolute spectral reflectance and transmittance. Appl. Opt. **20**(21), 3797–3804 (1981). 10.1364/AO.20.003797 20372262

[220] J. Peng, X. Wang, J. Zhou, Y. Zhang, H. Chen, Photothermal anti-icing/deicing superhydrophobic coatings: theory, mechanism and application. Nano Mater. Sci. **11**, 007 (2025). 10.1016/j.nanoms.2025.11.007

[221] M. Wang, J. Hu, M. Li, L. Zhang, M. Salimi et al., Bioinspired design of photothermal anti-fouling fabrics for solar-driven sustainable seawater desalination. Nano Energy **136**, 110726 (2025). 10.1016/j.nanoen.2025.110726

[222] L. Tang, B. Lyu, Y. Zhou, D. Gao, J. Ma, A moderately photothermal adjustable personal thermal management textile with integrated radiative and electric heating. Chem. Eng. Sci. **319**, 122344 (2026). 10.1016/j.ces.2025.122344

[223] Q. Zhang, H. Cheng, S. Zhang, Y. Li, Z. Li et al., Advancements and challenges in thermoregulating textiles: smart clothing for enhanced personal thermal management. Chem. Eng. J. **488**, 151040 (2024). 10.1016/j.cej.2024.151040

[224] W. Jiang, J.-Z. Liu, Z. Wang, T. Li, Y. Wang et al., Wearable passive thermal management functional textiles: recent advances in personal comfort and energy harvesting applications. Adv. Fiber Mater. **7**(6), 1677–1717 (2025). 10.1007/s42765-025-00581-2

[225] A. Ol, J.J. Savchuk, J. Carvajal, M. Massons, F. Aguiló, Díaz, Determination of photothermal conversion efficiency of graphene and graphene oxide through an integrating sphere method. Carbon **103**, 134–141 (2016). 10.1016/j.carbon.2016.02.075

[226] M. Braun, A. Waheed, K. von Figura, Lysosomal acid phosphatase is transported to lysosomes via the cell surface. EMBO J. **8**(12), 3633–3640 (1989). 10.1002/j.1460-2075.1989.tb08537.x PMC402045 2583113

[227] H. Ren, M. Tang, B. Guan, K. Wang, J. Yang et al., Hierarchical graphene foam for efficient omnidirectional solar–thermal energy conversion. Adv. Mater. **29**(38), 1702590 (2017). 10.1002/adma.201702590 28833544

[228] S. Li, Q. Deng, Y. Zhang, X. Li, G. Wen et al., Rational design of conjugated small molecules for superior photothermal theranostics in the NIR‐II biowindow. Adv. Mater. **32**(33), 2001146 (2020). 10.1002/adma.202001146 32627868

[229] Z. Xie, Y. Duo, Z. Lin, T. Fan, C. Xing et al., The rise of 2D photothermal materials beyond graphene for clean water production. Adv. Sci. **7**(5), 1902236 (2020). 10.1002/advs.201902236 PMC7055570 32154070

[230] Y. Zhou, K. Pei, Z. Zhou, L. Zhang, C. Tan et al., Synergistic solar-thermal icephobic armor: ultrasmall-bandgap MOFs drive efficient anti-/de-icing via superhydrophobic photothermal cooperation. eScience **6**, 100539 (2026). 10.1016/j.esci.2026.100539

[231] J. Li, D. Li, Y. Liu, L. Feng, X. Shi et al., Robust superhydrophobic fabrics: rapid preparation and outstanding photothermal-induced performance. Mater. Res. Bull. **178**, 112891 (2024). 10.1016/j.materresbull.2024.112891

[232] C.-H. Xue, M.-M. Du, X.-J. Guo, B.-Y. Liu, R.-X. Wei et al., Fabrication of superhydrophobic photothermal conversion fabric via layer-by-layer assembly of carbon nanotubes. Cellulose **28**(8), 5107–5121 (2021). 10.1007/s10570-021-03857-z

[233] Y. Liu, Q. Wang, T. Zhang, M. Hao, X. Hu et al., Fabrication of breathable superhydrophobic composite fabric with moisture permeability and weak-electric-field bactericidal ability designed for protective clothing applications. Mater. Des. **237**, 112612 (2024). 10.1016/j.matdes.2023.112612

[234] J. Zhang, P. Zhang, J. Liu, R. Zhang, W. Lin et al., Environmentally robust photothermal superhydrophobic coatings via a composition-interface synergistic reinforcement strategy for ultralow-temperature anti/de-icing. J. Mater. Sci. Technol. **263**, 117–128 (2026). 10.1016/j.jmst.2025.11.001

[235] K. Gu, H. Zhong, A general methodology to measure the light-to-heat conversion efficiency of solid materials. Light Sci. Appl. **12**(1), 120 (2023). 10.1038/s41377-023-01167-6 PMC10188530 37193685

[236] J. Luo, W. Sun, Z. Hu, Y. Feng, F. Chu et al., Photothermal superhydrophobic anti-icing surfaces necessitate dynamic thermal regulation. Matter **8**(10), 102389 (2025). 10.1016/j.matt.2025.102389

[237] S. Cheng, S.S. Latthe, K. Nakata, R. Xing, S. Liu et al., Recent advancements in design, development and demands of photothermal superhydrophobic materials. Mater. Today Chem. **35**, 101868 (2024). 10.1016/j.mtchem.2023.101868

[238] A. Paściak, A. Pilch-Wróbel, Ł Marciniak, P.J. Schuck, A. Bednarkiewicz, Standardization of methodology of light-to-heat conversion efficiency determination for colloidal nanoheaters. ACS Appl. Mater. Interfaces **13**(37), 44556–44567 (2021). 10.1021/acsami.1c12409 PMC8461604 34498862

[239] Y. Liu, Z. Lin, P. Wang, F. Huang, J.-L. Sun, Measurement of the photothermal conversion efficiency of CNT films utilizing a Raman spectrum. Nanomaterials **12**(7), 1101 (2022). 10.3390/nano12071101 PMC9000262 35407219

[240] Y. Xiao, H. Guo, M. Li, J. He, X. Xu et al., Strategies for enhancing the photothermal conversion efficiency of solar-driven interfacial evaporation. Coord. Chem. Rev. **527**, 216378 (2025). 10.1016/j.ccr.2024.216378

[241] Y. Yang, Y. Luo, B. Dong, B. Wu, Y. Zhou, A superhydrophobic photothermal cotton fabric for anti-icing/deicing and high-performance urea–formaldehyde foam. ACS Appl. Mater. Interfaces **17**(23), 34759–34770 (2025). 10.1021/acsami.5c04136 40434341

[242] W. Wu, Y. Zhang, S. Miao, Y. Wu, X. Gong, Photothermal superhydrophobic cotton fabric based on silver nanoparticles cross-linked by polydopamine and polyethylenimide. Langmuir **39**(42), 15131–15141 (2023). 10.1021/acs.langmuir.3c02269 37814887

[243] Q. Xue, J. Wu, Z. Lv, Y. Lei, X. Liu et al., Photothermal superhydrophobic chitosan-based cotton fabric for rapid deicing and oil/water separation. Langmuir **39**(28), 9912–9923 (2023). 10.1021/acs.langmuir.3c01144 37389997

[244] L. Zuo, C. Zhou, Z. Guo, An anti-icing textile employed in polar tent through thermal and electrical energy conversion. Chem. Eng. J. **521**, 166928 (2025). 10.1016/j.cej.2025.166928

[245] E. Li, Y. Pan, C. Wang, C. Liu, C. Shen et al., Asymmetric superhydrophobic textiles for electromagnetic interference shielding, photothermal conversion, and solar water evaporation. ACS Appl. Mater. Interfaces **13**(24), 28996–29007 (2021). 10.1021/acsami.1c07976 34101415

[246] C. Zhang, K. Pei, T. Sheng, M. Zhang, Z. Zhao et al., Metal oxides engineering: toward sustainable superhydrophobic surfaces. Adv. Funct. Mater. **36**(1), e12239 (2026). 10.1002/adfm.202512239

[247] X. Qi, J. Wu, L. He, W. Wei, J. Wang et al., Silane grafted graphene superhydrophobic coating coated non-woven fabric for photothermal driven high viscosity oil-water separation. Carbon **233**, 119886 (2025). 10.1016/j.carbon.2024.119886

[248] G. Qi, Y. Xiao, P. Zhang, X. Cui, H. Wang et al., A UV-curable photothermal superhydrophobic coating for efficient anti/de-icing and electricity generation. Prog. Org. Coat. **209**, 109558 (2025). 10.1016/j.porgcoat.2025.109558

[249] Y. Zhang, F. Yao, H. Tu, S. Peng, M. Huang et al., One-piece insulating superhydrophobic photothermal coating for suppression of icing and thermal aging of wind turbine blades. Prog. Org. Coat. **209**, 109630 (2025). 10.1016/j.porgcoat.2025.109630

[250] C. Liu, T. Lu, B. Cheng, R. Chen, Y. Huang et al., Preparation of a superhydrophobic GO/ZIF-67/PDMS composite fabric with high photothermal antibacterial performance and durability. Appl. Surf. Sci. **725**, 165862 (2026). 10.1016/j.apsusc.2026.165862

[251] X. Qin, Q. Cong, J. Xu, T. Chen, J. Jin et al., Photothermal superhydrophobic coupled functional surface with active anti/de-icing performance. Appl. Therm. Eng. **260**, 125031 (2025). 10.1016/j.applthermaleng.2024.125031

[252] J. Peng, X. Wang, J. Zhou, J. Wang, Y. Yang et al., Photothermal/electrothermal superhydrophobic composite coating for efficient anti-icing and de-icing. Compos. Part B Eng. **310**, 113144 (2026). 10.1016/j.compositesb.2025.113144

[253] Y. Wang, T. Jiang, J. Wu, S. Chen, M. Li et al., Superhydrophobic-photothermal-insulating synergistic CNT/COF composite coatings for solar-powered crude oil restoration. J. Hazard. Mater. **497**, 139690 (2025). 10.1016/j.jhazmat.2025.139690 40902533

[254] B. Zhang, X. Liu, M. Wang, B. Hou, TiN-loaded photothermal superhydrophobic coating with integrated passive anti-icing, active deicing, and anti-corrosion functions. Mater. Today Phys. **58**, 101857 (2025). 10.1016/j.mtphys.2025.101857

[255] X. Yang, Y. Liu, Y. Zhong, H. Chen, Anti/de-icing superhydrophobic coating with durability and self-healing by infiltrating photothermal self-stratifying organic layers into plasma-sprayed porous Al_2_O_3_–13%TiO_2_ underlayer. Surf. Interfaces **54**, 105305 (2024). 10.1016/j.surfin.2024.105305

[256] W. Qian, L. Zhou, A. Chen, Y. Liu, Designing multilayered C/PDMS/PolyF coatings with durable superhydrophobic and photothermal performances to address icing challenge in industrial environments. Appl. Surf. Sci. **710**, 163936 (2025). 10.1016/j.apsusc.2025.163936

[257] Q. Huang, Y. Chen, C. Jia, K. Yang, H. Wang et al., Epoxy resin/fluorinated multi-walled carbon nanotube composite photothermal superhydrophobic coatings for anti-icing applications. Prog. Org. Coat. **213**, 109912 (2026). 10.1016/j.porgcoat.2025.109912

[258] X. Zheng, H. Zhang, B. Chen, H. Shen, L. Li et al., Flexible and longterm superhydrophobic coating for anti-icing and photothermal de-icing. Prog. Org. Coat. **211**, 109729 (2026). 10.1016/j.porgcoat.2025.109729

[259] H. Ji, C. Yang, H. Su, Z. Qi, Y. Wang et al., Multifunctional superhydrophobic film with comprehensive icing monitoring, anti-icing, and photothermal/electrothermal deicing properties. Prog. Org. Coat. **206**, 109337 (2025). 10.1016/j.porgcoat.2025.109337

[260] T.N.H. Lo, I. Park, Photothermal superhydrophobic coatings based on wrinkled mesoporous carbon for efficient anti-icing and deicing. Carbon **243**, 120496 (2025). 10.1016/j.carbon.2025.120496

[261] L. Liming, C. Shenglong, P. Wei, Preparation and anti-icing performance of a photothermal self-healing superhydrophobic membrane. Acta Mater. Compos. Sin. **42**(3), 1436–1447 (2024). 10.13801/j.cnki.fhclxb.20240530.002

[262] L. Zhang, C. Gao, L. Zhong, L. Zhu, H. Chen et al., Robust photothermal superhydrophobic coatings with dual-size micro/nano structure enhance anti-/de-icing and chemical resistance properties. Chem. Eng. J. **446**, 137461 (2022). 10.1016/j.cej.2022.137461

[263] G. Zhang, C. Liang, A. Li, B. Lu, X. Li et al., Synergistic photothermal and superhydrophobic effects: design and molecular mechanism of anti-icing coatings based on MWCNTs-COOH/TiN@HDTMS. Appl. Surf. Sci. **720**, 165150 (2026). 10.1016/j.apsusc.2025.165150

[264] X. Song, K. Yin, X. Li, L. Wang, P. Yang et al., Efficient anti-icing/deicing via photothermal-wind synergistic effects on femtosecond laser-induced superhydrophobic graphene. J. Mater. Chem. A **13**(1), 205–213 (2025). 10.1039/D4TA06520D

[265] Y.-Y. Yan, C.-H. Xue, B.-Y. Liu, L. Wan, W.-J. Zhao et al., Robust photothermal superhydrophobic coating enabled by hybrid-dimensional fillers for effective anti-icing and de-icing. Colloids Surf. Physicochem. Eng. Asp. **734**, 139423 (2026). 10.1016/j.colsurfa.2025.139423

[266] Z. Liu, J. Hu, G. Jiang, Superhydrophobic and photothermal deicing composite coating with self-healing and anti-corrosion for anti-icing applications. Surf. Coat. Technol. **444**, 128668 (2022). 10.1016/j.surfcoat.2022.128668

[267] Y. Xue, Y. Wang, X. Sui, W. Liang, F. Wang, Superhydrophobic, photothermal, MXene/PEI composite film with anti-icing and deicing properties. Mater. Lett. **347**, 134638 (2023). 10.1016/j.matlet.2023.134638

[268] S. Sun, H.-Y. Feng, X. Shu, Q.-R. Xiao, A layer-by-layer coated highly conductive cotton fabric combining with superhydrophobicity, photothermal property, UV shielding, and temperature sensing. Prog. Org. Coat. **207**, 109387 (2025). 10.1016/j.porgcoat.2025.109387

[269] J. Peng, W. Han, T. Wu, L. Song, Y. Yin et al., A superhydrophobic e-fabric based on polydopamine template-assisted MXene/MWNTs with strain sensing, EMI shielding and electrothermal performance for health management. J. Alloys Compd. **990**, 174514 (2024). 10.1016/j.jallcom.2024.174514

[270] Q. Xu, J. Zhang, Y. Yang, P. Wang, Y. Zhang et al., Sub-zero temperature self-healing photothermal superhydrophobic coatings on PET fabric for multifunctional applications. Mater. Today Chem. **48**, 102919 (2025). 10.1016/j.mtchem.2025.102919

[271] N. Fu, H. Liu, J. Zhang, S. Wang, Y. Yang et al., Bioinspired multifunctional MXene-decorated textile for thermal management, durable self-cleaning, bio-protection and wearable strain sensor. Appl. Surf. Sci. **669**, 160607 (2024). 10.1016/j.apsusc.2024.160607

[272] X. Zong, C. Zhang, N. Zhang, Z. Wang, J. Wang, Breathable, superhydrophobic and multifunctional Janus nanofibers for dual-mode passive thermal management/facial expression recognition with deep learning. Chem. Eng. J. **505**, 159759 (2025). 10.1016/j.cej.2025.159759

[273] J. Du, Z. Guo, X. Yan, Y. Yao, R. Zhang et al., Flexible, stretchable multifunctional silver nanoparticles-decorated cotton textile based on amyloid-like protein aggregation for electrothermal and photothermal dual-driven wearable heater. Int. J. Biol. Macromol. **292**, 139124 (2025). 10.1016/j.ijbiomac.2024.139124 39722396

[274] K. Li, H.-N. Li, Y.-R. Xue, H.-C. Yang, C. Zhang et al., Photothermal Janus fabrics enabling persistent directional sweat-wicking in personal wet-thermal management. J. Colloid Interface Sci. **651**, 841–848 (2023). 10.1016/j.jcis.2023.08.044 37573730

[275] X. Yu, G. Xu, J. Huang, X. Zhao, J. Zhang, Scalable super-hydrophobic polyurethane/fluorinated polyurethane/SiO_2_ nanofibrous membranes for waterproof and breathable application. Fibers Polym. **26**(7), 2741–2749 (2025). 10.1007/s12221-025-00996-z

[276] S. Newby, W. Mirihanage, A. Fernando, Body heat energy driven knitted thermoelectric garments with personal cooling. Appl. Therm. Eng. **258**, 124546 (2025). 10.1016/j.applthermaleng.2024.124546

[277] G. Yu, G. Jiang, Fabrication of a superhydrophobic surface with photothermal electrical conversion performance for anti-icing. Surf. Interfaces **46**, 104089 (2024). 10.1016/j.surfin.2024.104089

[278] L. Noureen, Z. Xie, Y. Gao, M. Li, M. Hussain et al., Multifunctional Ag_3_PO_4_-rGO coated textiles for clean water production by solar-driven evaporation, photocatalysis, and disinfection. ACS Appl. Mater. Interfaces **12**, 6343–6350 (2020). 10.1021/acsami.9b16043 31939275

[279] X. He, J. Cai, M. Liu, X. Ni, W. Liu et al., Multifunctional, wearable, and wireless sensing system via thermoelectric fabrics. Engineering **35**, 158–167 (2024). 10.1016/j.eng.2023.05.026

[280] Y. Wang, H. Liu, S. Zhang, G. Tao, C. Liu et al., Radiation-modulated thermoelectric fabrics for wearable energy harvesting. Natl. Sci. Rev. **12**(7), nwaf215 (2025). 10.1093/nsr/nwaf215 PMC12236156 40630825

[281] H.M. Elmoughni, O. Atalay, K. Ozlem, A.K. Menon, Thermoelectric clothing for body heat harvesting and personal cooling: design and fabrication of a textile‐integrated flexible and vertical device. Energy Technol. **10**, 2200528 (2022). 10.1002/ente.202200528

[282] X. He, X.-L. Shi, X. Wu, C. Li, W.-D. Liu et al., Three-dimensional flexible thermoelectric fabrics for smart wearables. Nat. Commun. **16**(1), 2523 (2025). 10.1038/s41467-025-57889-1 PMC11906656 40082483

[283] M. Li, J. Chen, M. Luo, W. Zhong, W. Wang et al., Wearable thermoelectric 3D spacer fabric containing a photothermal ZrC layer with improved power generation efficiency. Energy Convers. Manag. **243**, 114432 (2021). 10.1016/j.enconman.2021.114432

[284] S. Newby, W. Mirihanage, A. Fernando, Wearable, knitted 3D spacer thermoelectric generator with detachable p-n junctions for body heat energy harvesting. Sensors **24**(16), 5140 (2024). 10.3390/s24165140 PMC11359260 39204837

[285] L. Yuan, S. Jia, S. Shao, H.M. Asfahan, X. Li, A self-cleaning Janus textile for highly efficient heating and cooling management. Nano Lett. **25**(19), 8019–8026 (2025). 10.1021/acs.nanolett.5c01738 40314160

[286] J. Chai, G. Wang, R. Shao, L. Ni, G. Zhao et al., Multifaceted Janus textile simultaneously achieving self-sustainable thermal management, perception, and protection. Nano-Micro Lett. **18**(1), 205 (2026). 10.1007/s40820-025-02030-6 PMC12799878 41528630

[287] A. Mortuza Saleque, A. Kumar Thakur, R. Saidur, M. Ismail Hossain, W. Qarony et al., RGO coated cotton fabric and thermoelectric module arrays for efficient solar desalination and electricity generation. J. Mater. Chem. A **12**, 405–418 (2023). 10.1039/D3TA04715F

[288] D. Ding, Q. Wu, J. Wang, Y. Chen, Q. Li et al., Superhydrophobic encapsulation of flexible Bi_2_Te_3_/CNT coated thermoelectric fabric via layer-by-layer assembly. Compos. Commun. **38**, 101509 (2023). 10.1016/j.coco.2023.101509

[289] X. Zhang, B. Shiu, T.-T. Li, X. Liu, H.-T. Ren et al., Synergistic work of photo-thermoelectric and hydroelectric effects of hierarchical structure photo-thermoelectric textile for solar energy harvesting and solar steam generation simultaneously. Chem. Eng. J. **426**, 131923 (2021). 10.1016/j.cej.2021.131923

[290] J. Li, Y. Wang, X. Fan, Y. Zhang, J.-H. Lin et al., Photothermal–thermoelectric synergistic enhancement of PEDOT/CNT/PPy flexible textile thermoelectric devices for waste heat and solar energy harvesting. ACS Appl. Energy Mater. **8**(19), 14477–14485 (2025). 10.1021/acsaem.5c02173

[291] X. Song, X. Li, B. Zhu, S. Sun, Z. Chen et al., MnO_2_/Poly-L-lysine co-decorated carbon fiber cloth with decreased evaporation enthalpy and enhanced photoabsorption/antibacterial performance for solar-enabled anti-fouling seawater desalination. Adv. Fiber Mater. **6**(5), 1569–1582 (2024). 10.1007/s42765-024-00437-1

[292] L. Wu, L. Wang, Z. Guo, J. Luo, H. Xue et al., Durable and multifunctional superhydrophobic coatings with excellent Joule heating and electromagnetic interference shielding performance for flexible sensing electronics. ACS Appl. Mater. Interfaces **11**(37), 34338–34347 (2019). 10.1021/acsami.9b11895 31441631

[293] J. Peng, H. Cheng, J. Liu, W. Han, T. Wu et al., Superhydrophobic MXene-based fabric with electromagnetic interference shielding and thermal management ability for flexible sensors. Adv. Fiber Mater. **5**(6), 2099–2113 (2023). 10.1007/s42765-023-00328-x

[294] P. Wang, J. Zhang, H. Wen, Z. Zhu, W. Huang et al., Photothermal conversion-assisted oil water separation by superhydrophobic cotton yarn prepared via the silver mirror reaction. Colloids Surf. Physicochem. Eng. Asp. **610**, 125684 (2021). 10.1016/j.colsurfa.2020.125684

[295] H. Zhang, Z. Guo, Robust MXene-based superhydrophobic fabrics for efficient separation of high-viscosity lubricating oil/water emulsions. New J. Chem. **49**(4), 1242–1250 (2025). 10.1039/D4NJ04989F

[296] S. Li, P. Xiao, W. Yang, C. Zhang, J. Gu et al., Hierarchically nanostructured Janus membranes toward sustainable and efficient solar-to-thermal management. Adv. Funct. Mater. **33**(18), 2209654 (2023). 10.1002/adfm.202209654

[297] Q. Zhang, M. Wang, H. Yu, N. Xu, X. Xiao et al., High-flux and anti-salt solar desalination via scalable photothermal textiles with vertically confined water layers. Device **3**(6), 100709 (2025). 10.1016/j.device.2025.100709

[298] J. Li, H. Qiao, Y. Chen, D. Xu, W. Li et al., Janus textile-based photo-electro-thermal evaporator for all-day sustainable desalination. Desalination **614**, 119203 (2025). 10.1016/j.desal.2025.119203

[299] W. Jia, L. Sun, X. Wang, B. Xiong, L. Wang et al., Janus-structured bilayer textile enabling high-efficiency salt-resistant solar desalination through directional water transport. Desalination **618**, 119460 (2026). 10.1016/j.desal.2025.119460

[300] C. Shi, Z. Wu, Y. Li, X. Zhang, Y. Xu et al., Superhydrophobic/superhydrophilic Janus evaporator for extreme high salt-resistance solar desalination by an integrated 3D printing method. ACS Appl. Mater. Interfaces **15**(19), 23971–23979 (2023). 10.1021/acsami.3c03320 37129548

[301] R.M. Irfan, S. Kim, J.Y. Lee, J. Jang, Scalable solar evaporator based on bandgap engineered CuMnCrO_4_ spinel oxide with salt‐resistant property for contaminated seawater. Adv. Mater. **38**, e17285 (2025). 10.1002/adma.202517285 PMC13003904 41400004

[302] D. Wang, X. Lin, Y. Wu, L. Li, W. Feng et al., Hanging photothermal fabric based on polyaniline/carbon nanotubes for efficient solar water evaporation. ACS Omega **8**(47), 44659–44666 (2023). 10.1021/acsomega.3c05332 PMC10688187 38046316

[303] R. Xu, H. Cui, N. Wei, Y. Yu, L. Dai et al., Biomimetic micro-nanostructured evaporator with dual-transition-metal MXene for efficient solar steam generation and multifunctional salt harvesting. Nano-Micro Lett. **17**(1), 102 (2025). 10.1007/s40820-024-01612-0 PMC11704123 39760777

[304] X. Cui, S. Dong, N. Cao, X. Zhang, J. Wang et al., Advanced solar-driven interfacial evaporation technology for resource and energy recovery. Energy Environ. Sci. **19**(2), 446–459 (2026). 10.1039/D5EE05041C

[305] G. Zhao, C. Wang, S. Qi, X. Zhang, X. Wang, Recent advances in fluorine-free superhydrophobic textiles: preparation, applications, and prospects. Prog. Org. Coat. **210**, 109693 (2026). 10.1016/j.porgcoat.2025.109693

[306] C.-C. Cai, J. Qin, Q.-K. Zhu, T.-T. Li, C.-W. Lou et al., All-weather self-healing superhydrophobic coating functionalized with the cauliflower-like PPy and TiN on cotton fabric for efficient oil-water separation. Prog. Org. Coat. **194**, 108566 (2024). 10.1016/j.porgcoat.2024.108566

[307] X. Lei, A. Xie, X. Yuan, X. Hou, J. Lu et al., Fabrication of superhydrophobic and light-absorbing polyester fabric based on caffeic acid. Polymers **14**(24), 5536 (2022). 10.3390/polym14245536 PMC9782021 36559903

[308] X. Chen, J. Wang, A. Xie, B. Wang, J. Wu et al., Fabrication of robust superhydrophobic polyester fabrics with photothermal conversion and oil–water separation performance through deposition of natural polyphenols. Langmuir **39**(44), 15817–15827 (2023). 10.1021/acs.langmuir.3c02508 37877472

[309] Z. Xiong, H. Yu, X. Gong, Designing photothermal superhydrophobic PET fabrics via in situ polymerization and 1,4-conjugation addition reaction. Langmuir **38**(28), 8708–8718 (2022). 10.1021/acs.langmuir.2c01366 35776847

[310] W. Liu, W. Cheng, M. Zhou, B. Xu, P. Wang et al., Construction of multifunctional UV-resistant, antibacterial and photothermal cotton fabric via silver/melanin-like nanoparticles. Cellulose **29**(13), 7477–7494 (2022). 10.1007/s10570-022-04740-1

[311] C. Zhang, K. Pei, J. Zhao, Y. Zhou, S. Zhang et al., Hierarchical dandelion-like superhydrophobic surfaces with excellent stability and photothermal performance for efficient anti-/deicing. Chem. Eng. J. **510**, 161582 (2025). 10.1016/j.cej.2025.161582

[312] J. Tao, Y. Liu, M. Li, Z. Li, Y. Zhang et al., Robust superhydrophobic composite fabric with self-healing and chemical durability. Small **20**(32), 2304894 (2024). 10.1002/smll.202304894 38546002

[313] W. Li, K. Liu, Y. Zhang, S. Guo, Z. Li et al., A facile strategy to prepare robust self-healable superhydrophobic fabrics with self-cleaning, anti-icing, UV resistance, and antibacterial properties. Chem. Eng. J. **446**, 137195 (2022). 10.1016/j.cej.2022.137195

[314] X. Jiao, M. Li, X. Yu, S. Yang, Y. Zhang, Mechanically robust superamphiphobic ceramic coatings with releasable nanoparticle-capsules. Chem. Eng. J. **446**, 137336 (2022). 10.1016/j.cej.2022.137336

[315] X. Li, X. Tan, T. Xiao, X. Li, L. Jiang et al., Superhydrophobic photothermal coatings based on polyurea for durable anti-icing and deicing. Surf. Interfaces **54**, 105120 (2024). 10.1016/j.surfin.2024.105120

[316] Y. Peng, J. Hu, Z. Fan, P. Xie, J. Wang et al., A stretchable superhydrophobic coating with electrothermal ability for anti-icing application. Mater. Res. Express **8**(4), 045009 (2021). 10.1088/2053-1591/abf475

[317] Hou M, Jiang Z, Sun W, Chen Z, Chu F et al (2024) Efficient photothermal anti‐/deicing enabled by 3D Cu_2__−__x_S encapsulated phase change materials mixed superhydrophobic coatings. Adv. Mater. 36(3):2310312. 10.1002/adma.202310312 37991469

[318] Y. Liu, Y. Wu, Y. Ma, P. Wang, B. Yu et al., Reversible thermochromic and heat storage coating for adaptive de/anti-icing and thermal regulation. Chem. Eng. J. **482**, 148837 (2024). 10.1016/j.cej.2024.148837

[319] F.P. Byrne, S. Jin, G. Paggiola, T.H.M. Petchey, J.H. Clark et al., Tools and techniques for solvent selection: green solvent selection guides. Sustain. Chem. Process. **4**(1), 7 (2016). 10.1186/s40508-016-0051-z

[320] G. Post, Development of health-based drinking water guidance for PFOA. Reprod. Toxicol. **27**, 423–423 (2009). 10.1016/j.reprotox.2008.11.075

[321] D. Prat, A. Wells, J. Hayler, H. Sneddon, C.R. McElroy et al., CHEM21 selection guide of classical- and less classical-solvents. Green Chem. **18**(1), 288–296 (2015). 10.1039/C5GC01008J

[322] M. Houde, J. Martin, R. Letcher, K. Solomon, D. Muir, Biological monitoring of polyfluoroalkyl substances: a review. Environ. Sci. Technol. **40**, 3463–3473 (2006). 10.1021/es052580b 16786681

[323] G.B. Post, P.D. Cohn, K.R. Cooper, Perfluorooctanoic acid (PFOA), an emerging drinking water contaminant: a critical review of recent literature. Environ. Res. **116**, 93–117 (2012). 10.1016/j.envres.2012.03.007 22560884

[324] T. Benn, B. Cavanagh, K. Hristovski, J. Posner, P. Westerhoff, The release of nanosilver from consumer products used in the home. J. Environ. Qual. **39**, 1875–1882 (2010). 10.2134/jeq2009.0363 PMC4773917 21284285

[325] L. Geranio, M. Heuberger, B. Nowack, The behavior of silver nanotextiles during washing. Environ. Sci. Technol. **43**(21), 8113–8118 (2009). 10.1021/es9018332 19924931

[326] M. Naguib, M. Kurtoglu, V. Presser, J. Lu, J. Niu et al., Two-dimensional nanocrystals: two-dimensional nanocrystals produced by exfoliation of Ti_3_AlC_2_. Adv. Mater. **23**(37), 4207–4207 (2011). 10.1002/adma.201190147 21861270

[327] S.H. Akyildiz, S. Fiore, M. Bruno, H. Sezgin, I. Yalcin-Enis et al., Release of microplastic fibers from synthetic textiles during household washing. Environ. Pollut. **357**, 124455 (2024). 10.1016/j.envpol.2024.124455 38942274

[328] U. Gliaudelytė, M. Persson, V. Daukantienė, Impact of textile composition, structure, and treatment on microplastic release during washing: a review. Text. Res. J. **95**(1–2), 220–232 (2025). 10.1177/00405175241260066

[329] B. Luzi, M. Carnevale Miino, E.C. Rada, R. Zullo, A.P.D. Baltrocchi et al., Critical review of microfiber release from textiles: results, comparative challenges, mitigation strategies, and legislative perspectives. Chemosphere **378**, 144394 (2025). 10.1016/j.chemosphere.2025.144394 40222242

[330] A.P. Periyasamy, Functionalized textile microplastics: a closer look at the issues, strategy, and legislation on the microplastic reduction. Kuwait J. Sci. **52**(3), 100395 (2025). 10.1016/j.kjs.2025.100395

[331] K. Parnell, A. Rolston, B. Hilton, A. Luccitti, Circular economy in the textile industry: a review of technology, practice, and opportunity. Recycling **10**(6), 225 (2025). 10.3390/recycling10060225

[332] H. Meng, Y. Zhou, S. Liu, Will the fluorine-free textiles cover us from the rain and dirt in the future? A review on current water repellent and stain resistant durable water repellents for textiles. Sci. Adv. Mater. **14**(11), 1654–1669 (2022). 10.1166/sam.2022.4274

[333] F. Gironi, V. Piemonte, Bioplastics and petroleum-based plastics: strengths and weaknesses. Energy Sources Part Recovery Util. Environ. Eff. **33**(21), 1949–1959 (2011). 10.1080/15567030903436830

[334] S. Alwarappan, N. Nesakumar, D. Sun, T.Y. Hu, C.-Z. Li, 2D metal carbides and nitrides (MXenes) for sensors and biosensors. Biosens. Bioelectron. **205**, 113943 (2022). 10.1016/j.bios.2021.113943 35219021

[335] Y. Gogotsi, B. Anasori, The rise of MXenes. ACS Nano **13**(8), 8491–8494 (2019). 10.1021/acsnano.9b06394 31454866

[336] H. Zhang, L. Zhang, B. Liu, Y. Zai, J. Gao et al., Progress of fluorine-free water repellents for textiles: a comprehensive review from industry and academia. Prog. Org. Coat. **213**, 109948 (2026). 10.1016/j.porgcoat.2026.109948

[337] D.R. Dreyer, S. Park, C.W. Bielawski, R.S. Ruoff, The chemistry of graphene oxide. Chem. Soc. Rev. **39**(1), 228–240 (2010). 10.1039/b917103g 20023850

[338] M.F.L.D. Volder, S.H. Tawfick, R.H. Baughman, A.J. Hart, Carbon nanotubes: present and future commercial applications. Science **339**(6119), 535–539 (2013). 10.1126/science.1222453 23372006

[339] J. Park, K. Shin, C. Lee, Roll-to-roll coating technology and its applications: a review. Int. J. Precis. Eng. Manuf. **17**(4), 537–550 (2016). 10.1007/s12541-016-0067-z

[340] T.H. Phung, A.N. Gafurov, I. Kim, S.Y. Kim, K.M. Kim et al., IoT device fabrication using roll-to-roll printing process. Sci. Rep. **11**(1), 19982 (2021). 10.1038/s41598-021-99436-0 PMC8497463 34620970

[341] R.R. Pingale, B.N. Altay, Reducing waste and increasing yield in roll-to-roll printing: a case study with global scalability. Int. J. Adv. Manuf. Technol. **141**(7–8), 4139–4153 (2025). 10.1007/s00170-025-16931-8

[342] M. Mohsin, S. Sardar, Development of sustainable and cost efficient textile foam-finishing and its comparison with conventional padding. Cellulose **27**(7), 4091–4107 (2020). 10.1007/s10570-020-03033-9

[343] A. Vyrkou, A. Angelis-Dimakis, T. Smith, P. Goswami, Environmental and economic impact assessment of hydrophobic treatment of cotton using low-pressure-low-temperature plasma. Clean. Eng. Technol. **22**, 100814 (2024). 10.1016/j.clet.2024.100814

[344] Z. Liu, Y. Zhang, Y. Li, Superhydrophobic coating for blade surface ice-phobic properties of wind turbines: a review. Prog. Org. Coat. **187**, 108145 (2024). 10.1016/j.porgcoat.2023.108145

[345] W. Bao, H. Shen, G. Zeng, Y. Zhang, Y. Wang et al., Engineering the next generation of MXenes: challenges and strategies for scalable production and enhanced performance. Nanoscale **17**(11), 6204–6265 (2025). 10.1039/d4nr04560b 39946163

[346] W. Zeng, X. Ye, Y. Dong, Y. Zhang, C. Sun et al., MXene for photocatalysis and photothermal conversion: synthesis, physicochemical properties, and applications. Coord. Chem. Rev. **508**, 215753 (2024). 10.1016/j.ccr.2024.215753

[347] J. Wang, M. Shen, Z. Liu, W. Wang, MXene materials for advanced thermal management and thermal energy utilization. Nano Energy **97**, 107177 (2022). 10.1016/j.nanoen.2022.107177

[348] P. Wang, X. Zhang, Z. Wang, T. Chen, H. Zhang et al., A robust superhydrophobic smart coating with reversible thermochromic and photochromic property. J. Bionic Eng. **19**(6), 1589–1600 (2022). 10.1007/s42235-022-00224-x

[349] H. Cheng, F. Wang, H. Liu, J. Ou, W. Li et al., Fabrication and properties of thermochromic superhydrophobic coatings. Adv. Eng. Mater. **24**(1), 2100647 (2022). 10.1002/adem.202100647

[350] X. Wang, Y. Peng, X. Wei, Y. Liang, J. Li et al., Robust multistage thermochromic superhydrophobic coating prepared by a facile one-step spray coating method. Prog. Org. Coat. **210**, 109625 (2026). 10.1016/j.porgcoat.2025.109625

[351] R. Zhang, Y. Zheng, C. Li, T. Zhang, J. Wang et al., Superhydrophobic and breathable nonwoven-based pressure–temperature bimodal tactile sensor without signal crosstalk. Fibers Polym. **26**(3), 1135–1146 (2025). 10.1007/s12221-025-00865-9

[352] M. Nazhipkyzy, Z.A. Mansurov, A. Amirfazli, A. Esbosin, T.S. Temirgaliyeva et al., Influence of superhydrophobic properties on deicing. J. Eng. Phys. Thermophys. **89**(6), 1476–1481 (2016). 10.1007/s10891-016-1516-3

[353] C. Ye, L. Zhao, S. Yang, X. Li, Recent research on preparation and application of smart joule heating fabrics. Small **20**(18), 2309027 (2024). 10.1002/smll.202309027 38072784

[354] X. Hao, L. Xu, Z. Cheng, Y. Guo, C. Yue et al., Construction of superhydrophobic surface for aircraft empennage applications: integrated photothermal de-icing, anti-corrosion and wear resistance functions. Aerosp. Sci. Technol. **174**, 111884 (2026). 10.1016/j.ast.2026.111884

[355] D. Wang, Q. Sun, M.J. Hokkanen, C. Zhang, F.-Y. Lin et al., Design of robust superhydrophobic surfaces. Nature **582**(7810), 55–59 (2020). 10.1038/s41586-020-2331-8 32494077

[356] Z. Zhang, Y. Liu, S. Chen, L. Liu, J. Liu et al., Design of superhydrophobic anti-icing coatings guided by full-process machine learning. Surf. Coat. Technol. **523**, 133235 (2026). 10.1016/j.surfcoat.2026.133235

[357] W. Huang, A. Samanta, Y. Chen, S. Baek, S.K. Shaw et al., Machine learning model for understanding laser superhydrophobic surface functionalization. J. Manuf. Process. **69**, 491–502 (2021). 10.1016/j.jmapro.2021.08.007

[358] D.G. Shaw, R. Liang, T. Zheng, J. Qi, J.D. Berry, Accurate and robust static hydrophobic contact angle measurements using machine learning. Langmuir **40**(32), 16757–16770 (2024). 10.1021/acs.langmuir.4c01050 39079054

[359] H.P. Mamgain, M.V. Diamanti, P.R. Pati, M.E. Mohamed, J.K. Pandey et al., Machine learning-driven stability analysis of eco-friendly superhydrophobic graphene-based coatings on copper substrate. Sci. Rep. **15**(1), 34602 (2025). 10.1038/s41598-025-18155-y PMC12494925 41044316

[360] M. Iannacchero, J. Löfgren, M. Mohan, P. Rinke, J. Vapaavuori, Machine learning-assisted development of polypyrrole-grafted yarns for e-textiles. Mater. Des. **249**, 113528 (2025). 10.1016/j.matdes.2024.113528

[361] G. Bai, Z. Wang, H. Wang, C. Wang, X. Liu et al., Machine learning-guided exploration of carbon-based photothermal materials for solar evaporation. Sep. Purif. Technol. **373**, 133627 (2025). 10.1016/j.seppur.2025.133627

[362] M. Alverson, S.G. Baird, R. Murdock, (Enoch)S. Ho, J. Johnson et al., Generative adversarial networks and diffusion models in material discovery. Digit. Discov. **3**(1), 62–80 (2024). 10.1039/D3DD00137G

[363] I. Malashin, D. Martysyuk, V. Tynchenko, A. Gantimurov, V. Nelyub et al., Machine learning in polymeric technical textiles: a review. Polymers **17**(9), 1172 (2025). 10.3390/polym17091172 PMC12073533 40362956

[364] Z. Liang, Y. Deng, Z. Shi, X. Liao, H. Zong et al., AI-driven design of powder-based nanomaterials for smart textiles: from data intelligence to system integration. Adv. Powder Mater. **5**(1), 100356 (2026). 10.1016/j.apmate.2025.100356

